# Programmable Carrier‐Free All‐Enzyme Beads for Modular Continuous‐Flow Biocatalysis

**DOI:** 10.1002/adma.75120

**Published:** 2026-09-24

**Authors:** Jennifer Kühne, Julian S. Hertel, André Delavault, Judith Felk, Martin Peng, Lara Reuber, Felix Ott, Kim Wanner, Marc F. Münker, Adrian Neukirch, Alexei Kiselev, Kersten S. Rabe, Christof M. Niemeyer

**Affiliations:** ^1^ Institute for Biological Interfaces (IBG1) Karlsruhe Institute of Technology (KIT) Eggenstein‐Leopoldshafen Germany; ^2^ Institute of Meteorology and Climate Research Department of Atmospheric Aerosol Research (IMK‐AAF) Karlsruhe Institute of Technology (KIT) Eggenstein‐Leopoldshafen Germany

**Keywords:** all‐enzyme materials, carrier‐free biocatalysis, continuous‐flow catalysis, modular catalytic architectures, multi‐enzyme cascades, programmable protein assemblies

## Abstract

Carrier‐free enzyme materials offer maximal catalytic density but are typically limited to monolithic or amorphous architectures with restricted process compatibility. Here, we introduce programmable carrier‐free all‐enzyme beads as structurally defined, porous biocatalytic particles for modular continuous‐flow operation. The beads are generated via droplet‐based self‐assembly of complementary enzyme building blocks, followed by cryogenic consolidation, yielding mechanically robust and monodisperse protein particles that retain full catalytic competence after drying and rehydration. Using mechanistically distinct model systems, including cofactor‐independent decarboxylation and metal‐dependent C─C bond formation, we demonstrate stable long‐term continuous‐flow operation exceeding 80 h and compatibility with biphasic solvent systems at elevated substrate concentrations. Beyond single‐enzyme catalysis, the bead architecture accommodates binary cofactor‐regenerating assemblies, integrated ternary cascades with dual cofactor recycling, and modular combinations of distinct bead populations enabling sequential nucleotide phosphorylation. Notably, flow‐induced restructuring leads to adaptive consolidation of packed beds, enhancing effective catalytic volume without compromising mass transport. By uniting structural programmability, high catalytic density, architectural modularity, and adaptive behavior under flow, carrier‐free all‐enzyme beads bridge molecular enzyme engineering and reactor design, expanding the materials toolbox for scalable and reconfigurable continuous biocatalysis.

## Graphical abstract

Genetically encoded enzyme building blocks self‐assemble into monodisperse, carrier‐free protein beads via a droplet‐based formulation strategy. These programmable catalytic particles enable modular continuous‐flow biocatalysis, from single‐enzyme reactions to multi‐enzyme cascades and bead–bead coupled reactor systems.

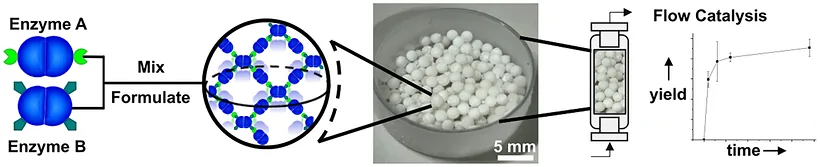

## 1 Introduction

Biocatalysis enables highly selective chemical transformations under mild reaction conditions and has become an important technology for fine chemical and pharmaceutical synthesis [1, 2, 3, 4, 5]. For implementation in continuous processes, however, enzymes must be organized in a format that ensures structural stability, high volumetric productivity, and efficient mass transport. Continuous‐flow biocatalysis provides defined residence times, improved process control, and compatibility with multistep cascades [6, 7, 8, 9, 10, 11], but its performance critically depends on the design of suitable immobilized enzyme materials.

Conventional immobilization approaches rely on the attachment of enzymes to inert carrier materials [12, 13, 14]. Although these systems facilitate catalyst recovery, the presence of non‐functional support phases reduces catalytic density and can impose mass‐transfer limitations, particularly in compact flow reactors where volumetric efficiency is decisive. Eliminating inert carrier material while maintaining structural integrity therefore represents a central challenge in the development of high‐performance enzyme materials.

Carrier‐free immobilization strategies address this issue by transforming enzymes into self‐supporting catalytic matter. Cross‐linked enzyme aggregates (CLEAs) [12, 15], catalytically active inclusion bodies (CatIBs) [16, 17], and genetically encoded protein crystals [18, 19] achieve high packing densities through chemical or biologically guided self‐assembly. However, these materials are typically amorphous, particulate powders, or crystalline solids with limited control over internal porosity and geometry, which can restrict mass transport and complicate their integration into structured reactor formats.

All‐enzyme hydrogels (AEHs) constitute a distinct class of carrier‐free materials in which complementary enzyme building blocks assemble into continuous, porous protein networks via genetically encoded and orthogonal interaction domains [20, 21, 22, 23, 24, 25]. This site‐defined assembly strategy avoids non‐specific chemical crosslinking and enables controlled network formation under mild aqueous conditions. In AEHs, catalytic and cofactor‐regenerating enzymes can be integrated within a single matrix, providing high catalytic density, tunable porosity, and efficient cofactor retention during flow operation. Despite these advantages, their monolithic hydrogel format offers limited geometric definition and is less suited for standardized packed‐bed configurations.

Translating all‐enzyme hydrogel architectures into discrete, structurally defined particles would combine carrier‐free catalytic density with geometric control and reactor compatibility. Shape‐ and size‐defined bead materials promise improved packing behavior, reproducible dosing, and scalable integration into continuous‐flow systems while preserving the absence of inert support phases.

Here, we introduce a droplet‐based formulation strategy that converts self‐assembling all‐enzyme hydrogels into monodisperse, carrier‐free protein beads (Figure 1). Complementary enzyme building blocks are combined within defined aqueous microdroplets and consolidated by controlled residence time and cryogenic solidification, yielding mechanically robust particles with tunable dimensions and preserved catalytic activity. We systematically analyze their structural and mechanical properties and demonstrate functional versatility across cofactor‐independent and cofactor‐dependent reactions, multi‐enzyme cascades, and modular bead–bead reactor configurations. By establishing programmable carrier‐free all‐enzyme beads as architected catalytic building blocks, this work advances the design of enzyme‐based materials toward modular and reconfigurable continuous‐flow biocatalysis.

**FIGURE 1:**
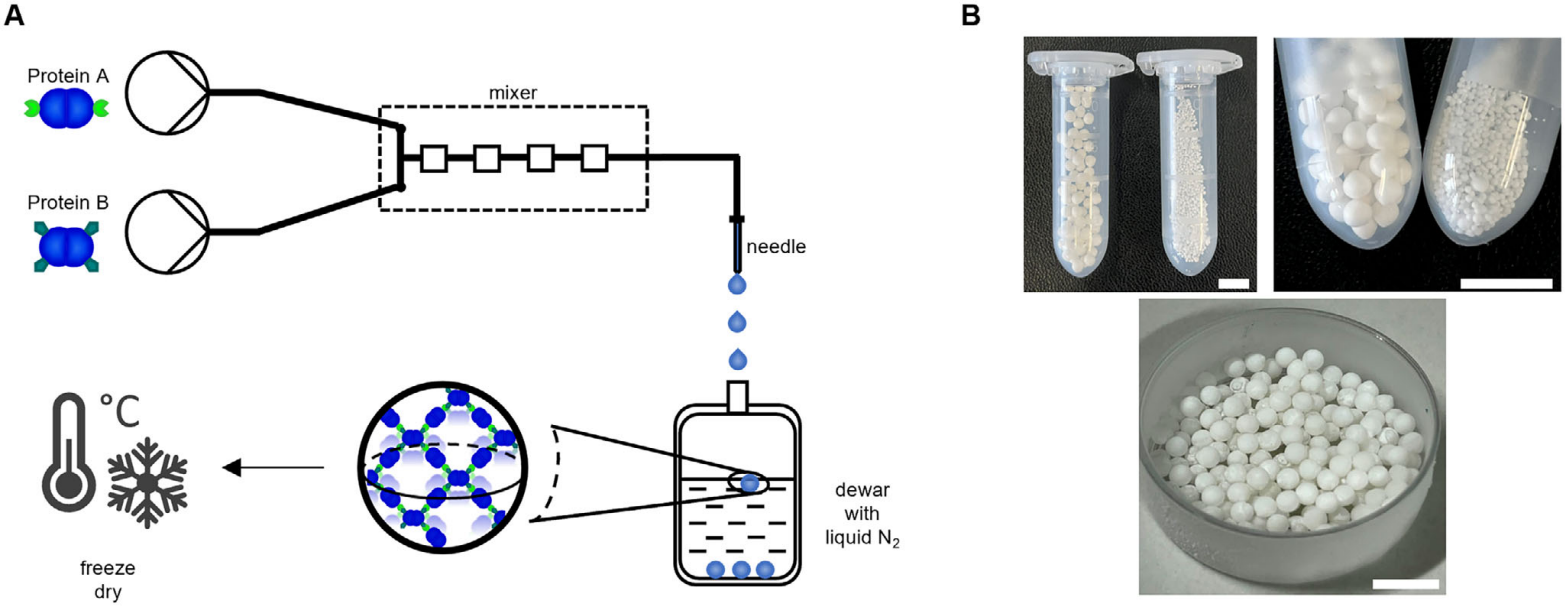
Formulation of monodisperse AEH beads. (A) Schematic representation of the formation of monodisperse protein beads through self‐assembly of complementary dimeric enzymes, protein A and protein B, combined with a droplet‐based formulation process. Aqueous enzyme solutions are supplied via two syringe pumps operated at equal flow rates and merged in a four‐chamber static mixer to ensure homogeneous blending. The resulting precursor mixture passes through a chemically inert tubing section, where the residence time defines the degree of crosslinking. Droplets are subsequently generated at a stainless‐steel needle and fall by gravity into liquid nitrogen, where they solidify into spherical particles via cryogenic curing. (B) Dried spherical beads with tunable size are obtained after freeze‐drying. The average particle diameter is independent of flow rate but determined by needle geometry, surface coating, and outlet diameter. Scale bars: 5 mm.

## 2 Results and Discussion

### 2.1 Fabrication and Structural Characterization of AEH Beads

To generate covalently cross‐linked monodisperse AEH beads, we employed the prokaryotic phenolic acid decarboxylase (EsPAD, EC 4.1.1.102) from *Enterobacter sp*., which has served as a well‐established model enzyme in our previous studies on all‐enzyme hydrogels and related immobilization systems [22, 25, 26, 27]. In these earlier works, EsPAD was successfully applied both in covalent hydrogel networks using the SpyTag/SpyCatcher (ST/SC) system [22] and in non‐covalent assemblies based on modular protein–protein interaction motifs [25], demonstrating its robustness and suitability for self‐assembling biocatalytic materials. Here, dimeric EsPAD was fused with either one SpyCatcher domain (EsPAD‐SC) or two SpyTag domains (ST‐EsPAD‐ST) to enable defined covalent crosslinking. Mixing these complementary fusion proteins in a 2:1 molar ratio of SC‐ to ST‐functionalized enzyme rapidly induces isopeptide bond formation between SC and ST domains, resulting in a densely crosslinked enzyme network that spontaneously forms monodisperse aggregates (Figure 2A). Deviations from this stoichiometric ratio lead to a lower degree of crosslinking and consequently reduced material stability [22].

**FIGURE 2:**
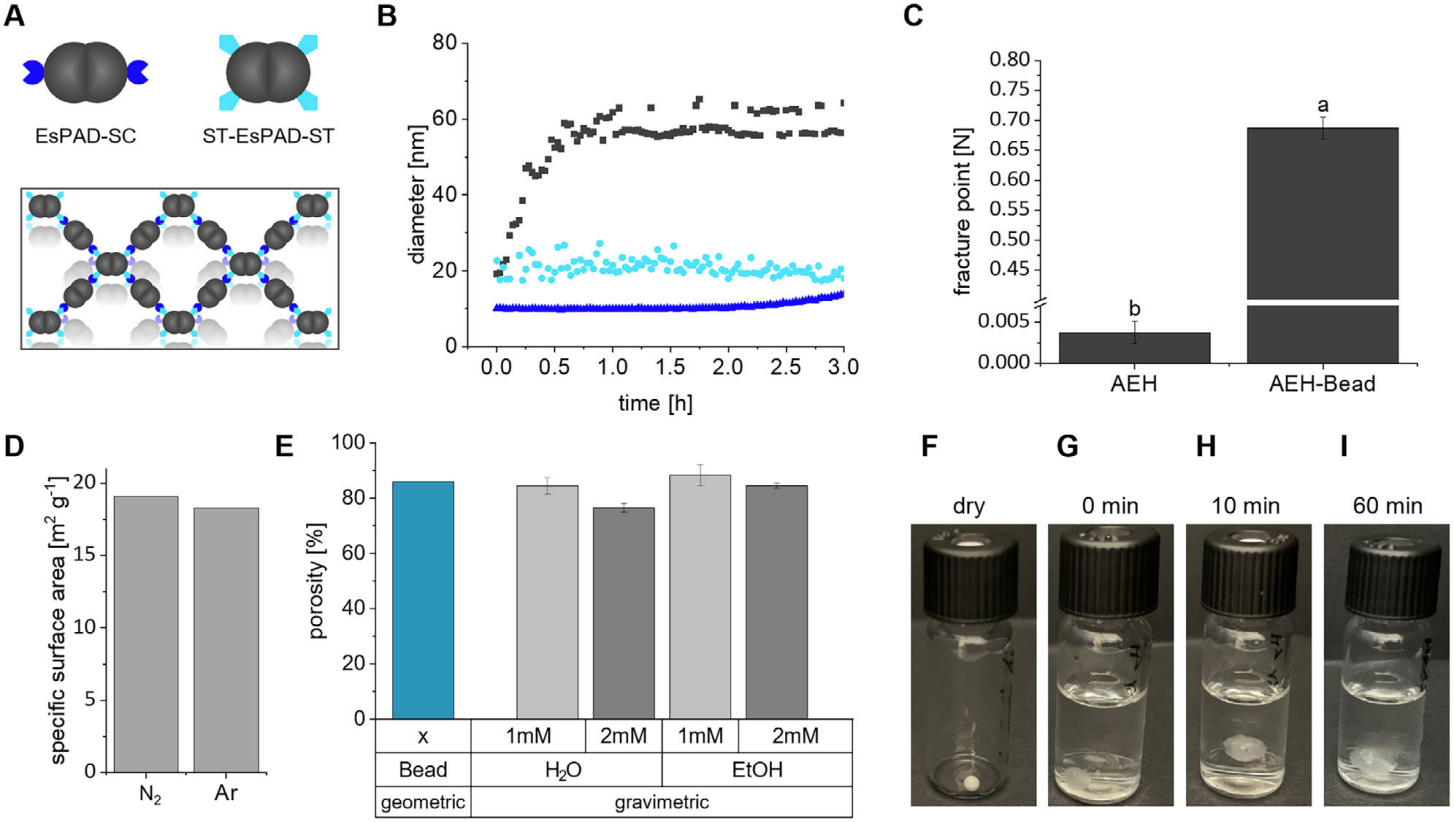
Characterization of AEH bead materials. (A) Schematic illustration of the formation of a three‐dimensional enzyme network through self‐assembly of dimeric EsPAD fusion proteins, EsPAD‐SC (blue) and ST‐EsPAD‐ST (turquoise). (B) Time‐dependent DLS analysis of nanogel formation at a total protein concentration of 1 mM, showing the hydrodynamic diameter measured every 100 s over 3 h at 25°C for an equimolar protein mixture (gray) and for the individual components EsPAD‐SC (blue) and ST‐EsPAD‐ST (turquoise). (C) Fracture point analysis of native AEH material and AEH beads using a microindentation platform; both samples were prepared from the same phenolic acid decarboxylase (EsPAD) carrying either SpyCatcher or SpyTag domains that covalently crosslink into a polymeric network. (D) Comparison of specific surface areas (SSA) obtained from N_2_ and Ar adsorption measurements. (E) Porosity of monodisperse AEH beads determined geometrically (based on ESEM) and gravimetrically using water and ethanol as probe solvents at total enzyme concentrations of 1 and 2 mM. (F–I) Visual evolution of 2 mM AEH bead integrity in aqueous buffer inside a 1 mL vial: dry state, 0, 10, and 60 min under shaking at 800 rpm, illustrating resistance to dissolution and long‐term shape retention. Unless otherwise noted, measurements were performed in triplicate (*n* = 3) and are presented as mean ± standard deviation.

We first investigated whether covalent coupling between the complementary EsPAD fusion enzymes promotes spontaneous self‐assembly of nanogel particles in solution. To this end, EsPAD‐SC and ST‐EsPAD‐ST were heterologously expressed in *E. coli*, purified via Ni–NTA affinity chromatography (Figure S1A, lanes 1 and 2; schematic representation in Figure S1B; sequence information for all enzymes in Table S1), and the expected SC/ST coupling was verified by SDS–PAGE analysis (Figure S2). Dynamic light scattering (DLS) was then employed to monitor time‐dependent changes in hydrodynamic radius for each purified protein and for equimolar mixtures of both components (Figure 2B). The isolated EsPAD‐SC (dark blue) and ST‐EsPAD‐ST (turquoise) displayed hydrodynamic diameters of approximately 10–20 nm, consistent with their expected dimeric state and indicating that no spontaneous aggregation occurs in the absence of the complementary binding partner. In contrast, equimolar mixtures of both proteins exhibited a time‐dependent increase in hydrodynamic diameter, indicating the formation of AEH nanogels via specific SC/ST‐mediated crosslinking. The EsPAD system showed rapid assembly kinetics, with an apparent growth rate of approximately 1.5 nm in diameter per minute and reaching an average particle size of ∼60 nm. These dimensions are in good agreement with previous DLS and atomic force microscopy analyses of related AEH nanogel systems, which typically display particle sizes in the range of 30–100 nm [20, 23, 24]. The observed size evolution thus confirms controlled nanoscale network formation prior to macroscopic bead consolidation.

To convert the self‐assembling enzyme networks into geometrically defined, flow‐compatible particles, we implemented a droplet‐based formulation strategy, yielding structures hereafter termed AEH beads. A continuous mixing platform (Figure 1A) was used to combine solutions of orthogonally tagged enzymes, followed by passage through a defined tubing segment (20 cm length, 0.38 mm inner diameter) that provided sufficient residence time for crosslinking prior to droplet formation. The residence time can be readily adapted to protein systems with differing assembly kinetics. The resulting droplets were generated at a stainless‐steel needle and collected in liquid nitrogen, where rapid cryogenic solidification preserved spherical geometry. A PTFE coating ensured controlled droplet detachment and prevented coalescence during gravity‐assisted formation (Figure S3). Subsequent lyophilization yielded mechanically stable, macroporous AEH beads with typical average diameters of 2.0 ± 0.1 mm (Figure S4A,B). Bead dimensions could be readily adjusted by reducing the initial droplet volume from 10 to 2 µL, yielding correspondingly smaller beads that remained catalytically active in batch and during continuous‐flow operation for up to 46 h (Figure S4C–E).

To assess the mechanical integrity of the freeze‐dried AEH beads, fracture point measurements were performed using a microindentation platform recently established for soft biopolymeric materials [28]. The cured beads exhibited markedly enhanced mechanical stability, with a fracture point of 0.69 ± 0.03 N compared to 0.003 ± 0.002 N for the uncured, native AEH material. This roughly 200‐fold increase in mechanical resistance demonstrates that the combined effects of covalent bioconjugation and physical cryogenic curing yield a structurally robust, self‐supporting enzymatic material (Figure 2C).

Complementary gas adsorption analyses yielded specific surface areas of 19.1 m^2 ^g^−1^ (N_2_) and 18.3 m^2^ g^−1^ (Ar) for the AEH beads (Figure 2D and Figure S5). The close agreement between the two probe gases, despite the quadrupolar nature of N_2_ and the non‐polar character of Ar, indicates homogeneous surface chemistry and uniform accessibility throughout the porous protein network. The absence of hysteresis in the desorption isotherms further demonstrates reversible adsorption behavior and structural stability of the macroporous architecture under measurement conditions. The minimal deviation (±1 m^2 ^g^−1^) between independent Brunauer–Emmett–Teller (BET) determinations underscores the robustness of the surface area analysis and confirms that the internal pore structure is consistently accessible, a prerequisite for efficient mass transport under continuous‐flow operation.

To quantify the accessible pore volume of the AEH beads, a straightforward gravimetric solvent‐uptake method was applied using water and ethanol as probe liquids (Figure 2E). The analysis revealed high porosity values between 76% and 88%, depending on protein concentration and solvent. At a total protein concentration of 1 mM, porosity reached 84% for water and 88% for ethanol, consistent with the enhanced wetting of ethanol due to its lower surface tension. Increasing the protein concentration to 2 mM reduced the porosity to 76% (water) and 84% (ethanol), indicating the formation of a denser crosslinked network with reduced pore accessibility. These values are in good agreement with geometric porosity estimates obtained from environmental scanning electron microscopy (ESEM) image analysis (≈85%), supporting the robustness of the structural characterization.

These data confirm that protein concentration directly controls the degree of crosslinking and, consequently, the internal architecture and mechanical performance of the AEH beads. The visual comparison in Figure 2F–I illustrates these differences, showing that denser formulations display enhanced rigidity and shape retention in aqueous environments. This ability to fine‐tune both biochemical and physical parameters demonstrates the versatility of AEH bead formation and highlights their potential as robust, carrier‐free enzyme materials for biocatalytic applications in both batch and continuous‐flow operation.

To gain insight into the internal architecture of the AEH beads, ESEM was performed (Figure 3). The images confirm the spherical morphology of the particles. Surface views (Figure 3D) reveal a continuous and largely uniform outer layer, whereas cross‐sectional images of the interior (Figure 3E–H) expose a heterogeneous macroporous network composed of interconnected voids and regions of varying structural density. Rather than displaying a single characteristic pore size, the internal morphology appears hierarchically organized, with larger cavities connected by smaller channels and a corrugated internal surface topology. This observation is consistent with the high gravimetric porosity of the material and the specific surface area determined from gas adsorption measurements. The rough internal topology suggests that the accessible surface area arises not only from large voids but also from a highly structured pore interface. The heterogeneous pore architecture likely originates from spatial differences in solvent removal during freeze‐drying: while dehydration proceeds rapidly at the particle surface, mass transport from the interior is slower, leading to local variations in pore formation and network consolidation.

**FIGURE 3:**
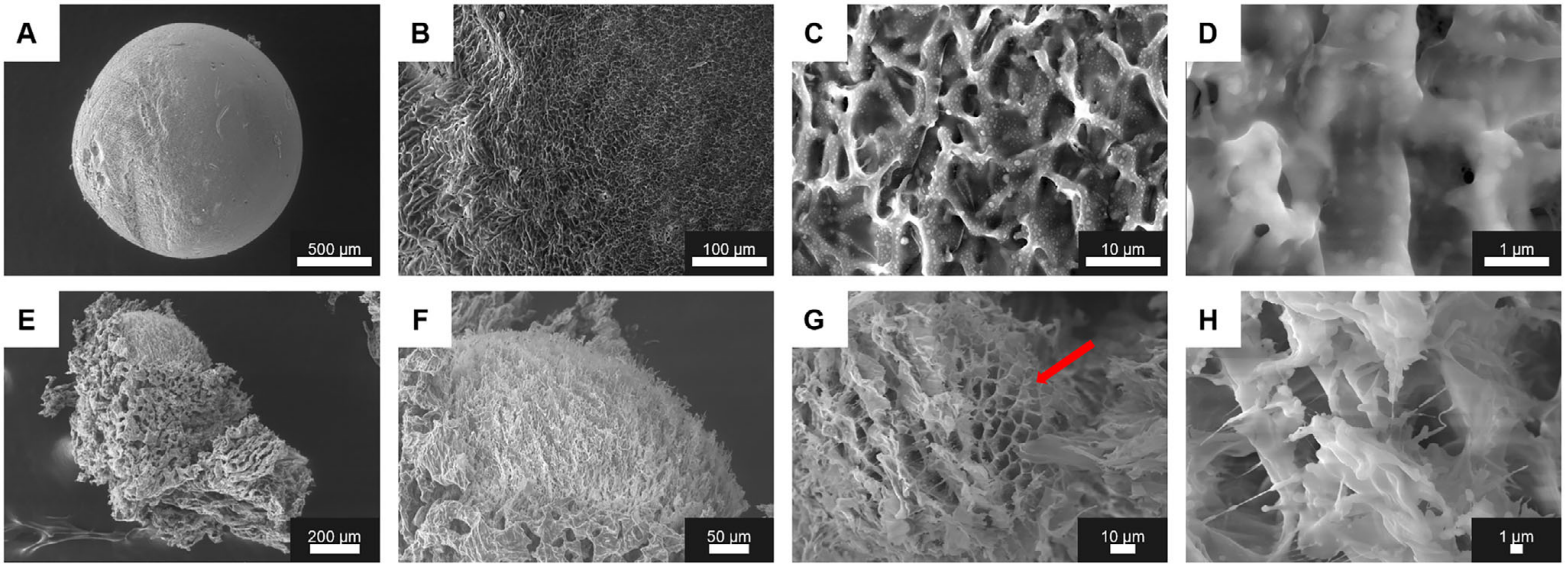
Environmental scanning electron microscopy (ESEM) analysis of AEH beads. (A–D) Surface morphology of an intact AEH bead showing a continuous and uniform outer layer. (E–H) Cross‐sectional views of a fractured bead revealing the heterogeneous internal architecture composed of interconnected macropores and regions of varying structural density. Scale bars: 500–1 µm.

A particularly notable feature is the presence of angular, occasionally polygonal motifs within the bead interior (Figure 3G, arrow). These faceted patterns resemble polyhedral cell boundaries commonly observed in foam‐like materials, where adjacent voids adopt multifaceted interfaces to minimize interfacial energy. Such structures likely arise during freezing and subsequent sublimation, when closely packed water‐filled domains within the protein network become immobilized and later leave behind interconnected cavities during lyophilization. The resulting pore boundaries therefore reflect the packing geometry of adjacent voids within the confined protein matrix. Similar polyhedral motifs have previously been observed in enzyme‐based foam materials reported by our group [24, 25], supporting the interpretation that local packing and interfacial tension within the hydrated network can template the final pore architecture.

To gain insight into the transition from nanoscale AEH assemblies to macroscopic beads, we monitored the thermal history and covalent crosslinking state throughout cryogenic processing and lyophilization (Figures S6 and S7). Temperature profiles revealed a prolonged warming and drying phase for the water‐containing bead preparation (Figure S6), consistent with the heterogeneous freeze‐concentrated domains expected upon freezing of aqueous solutions [29, 30]. SDS‐PAGE showed that SC/ST‐mediated crosslinking is initiated before exposure to liquid nitrogen and that covalently crosslinked material persists throughout lyophilization (Figure S7). Thus, initial network formation precedes freezing, while ice formation is expected to concentrate the protein building blocks within freeze‐concentrated domains, increasing their local concentration and encounter probability. Further network consolidation during the warming and drying phase is mechanistically plausible, as increasing temperature in the presence of residual hydration may enhance molecular mobility and productive SC/ST encounters [31]. Although freezing may concurrently perturb protein structure [32], bead formation proved robust toward variation of the lyophilization conditions, yielding intact and catalytically active materials (Figure S8). The resulting interconnected porous architecture observed by ESEM (Figure 3E–H) is consistent with removal of ice‐rich domains during sublimation, while fluorescence microscopy shows broad spatial co‐distribution of protein components within the investigated regions of the bead matrix (Figure S9). Together, these observations support a mechanism in which solution‐phase AEH assembly [33] is followed by freeze‐induced confinement, maintenance, and potential further consolidation of the covalent network, and ice‐templated consolidation into the macroscopic porous bead. A detailed mechanistic discussion is provided in the Supporting Information following Figure S9.

### 2.2 Catalytic Performance of AEH Beads

The catalytic performance of the monodisperse AEH beads was evaluated using the established EsPAD model reaction, that is, the decarboxylation of p‐coumaric acid to 4‐vinylphenol (Figure 4A), which previously served as a benchmark to compare different enzyme material formats and coupling strategies [25]. When comparing catalytic performance, the effects of genetic fusion and subsequent heterogenization need to be distinguished. The influence of genetic fusion on enzyme activity is generally difficult to predict, as both increases and decreases have been reported and may result from alterations in protein structure and dynamics [34, 35]. Accordingly, the native enzymes and their interaction‐domain fusion variants used here show construct‐dependent changes in specific activity, ranging from retained or moderately increased to reduced activity (Table S3). For example, specific activities of 30.95, 19.2, and 40.40 µmol min^−1^ mg^−1^ were determined for EsPAD‐His, EsPAD‐SC, and ST‐EsPAD‐ST, respectively. Based on the SC/ST composition used for bead formation, this corresponds to a theoretical specific activity of 26.27 µmol min^−1^ mg^−1^ for the soluble enzyme mixture, compared with an apparent mass‐specific activity of 0.73 µmol min^−1^ mg^−1^ for the resulting AEH beads, corresponding to an apparent activity retention of approximately 2.8%. Importantly, the activity of the macroscopic AEH beads additionally reflects substrate accessibility, mass transport, network architecture, and rehydration and therefore cannot be directly interpreted as intrinsic enzyme‐specific activity or quantitative molecular inactivation upon immobilization. Nevertheless, catalytic activity after the complete cryogenic fabrication and lyophilization process provides a functional measure of retained enzymatic competence. Since bead fabrication involves no intermediate separation or washing step, the total amount of protein introduced into each droplet is taken as the initial bead loading and is used for catalyst loading and benchmarking throughout this study; this initial loading should be distinguished from protein retention during subsequent catalysis, which can depend on network stability and bead swelling or partial dissolution.

**FIGURE 4:**
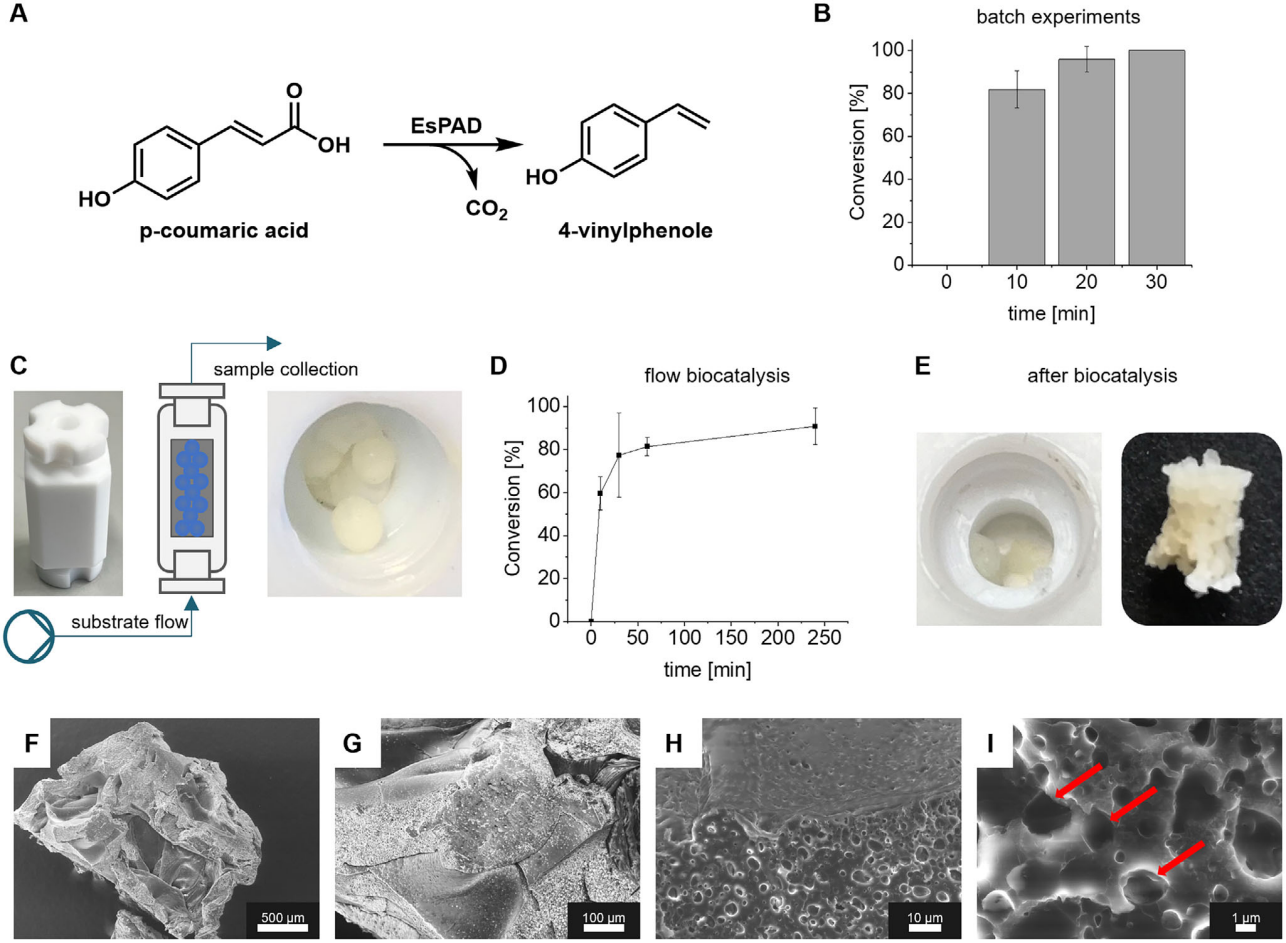
Catalytic performance of AEH bead reactors under batch and continuous‐flow operation. (A) Reaction scheme of the one‐step enzymatic decarboxylation of p‐coumaric acid (pCA) to 4‐vinylphenol (4VP) catalyzed by EsPAD. (B) Conversion of 5 mM pCA to 4VP in a 1 mL batch reactor within 30 min, confirming full enzymatic activity of the AEH beads. (C) Schematic representation of the continuous‐flow setup using a PTFE‐based tubular reactor packed with AEH beads. (D) Time‐dependent substrate conversion in the reactor operated at a constant flow rate of 15 µL min^−1^ over 250 min in a biphasic MTBE/water system (95:5 *v/v*), enabling dissolution of 100 mM p‐coumaric acid and demonstrating continuous catalytic operation under intensified biphasic reaction conditions. All measurements were performed in triplicate (*n* = 3) and are presented as mean ± standard deviation. (E) Visual inspection of bead integrity after continuous operation, showing no apparent structural degradation. (F–I) Environmental scanning electron microscopy (ESEM) images of a section of fused AEH beads after catalysis, displayed at increasing magnifications. Scale bars: 500–1 µm.

Under batch conditions, chromatographic analysis revealed quantitative conversion of 5 mM substrate within 30 min (Figure 4B), which was reproducible upon reuse of the same bead over multiple reaction cycles (Figure S10). Notably, progressive macroscopic morphological changes were observed during repeated use, while catalytic activity was retained, indicating that restructuring of the hydrated bead does not directly correspond to catalytic deactivation. In direct comparison to earlier hydrogel and non‐covalent material formulations, these results demonstrate that translating the nanoscale self‐assembly principle into a cryogenically consolidated bead architecture retains high catalytic performance. Instead, the AEH beads remain catalytically competent while providing a structurally reinforced, macroscopic format suitable for handling and reactor integration, thereby extending the materials platform toward process‐relevant geometries.

Following optimization of enzyme loading, substrate concentration, and residence time, the system was transferred to a continuous‐flow setup based on a PTFE tubular reactor [36]. Four monodisperse AEH beads (≈18 nmol immobilized enzyme) were packed into the reactor, forming a defined micro‐scale configuration (Figure 4C). At a constant flow rate of 15 µL min^−1^, high substrate conversion was achieved during continuous operation for more than 250 min (Figure 4D), demonstrating efficient continuous‐flow catalysis under these conditions. Notably, the reaction was conducted in a biphasic methyl tert‐butyl ether (MTBE)/water system (95:5 *v/v*), enabling dissolution of 100 mM p‐coumaric acid, approximately twenty‐fold higher than achievable under purely aqueous conditions [22]. The ability of the carrier‐free enzyme beads to operate efficiently at elevated substrate concentrations and in the presence of a predominantly organic solvent phase highlights the structural integrity and solvent tolerance of the crosslinked protein network. This combination of high volumetric activity, mechanical stability, and compatibility with non‐aqueous reaction environments underscores the suitability of the AEH bead architecture for intensified continuous‐flow biocatalysis and modular reactor integration [37].

Upon completion of the continuous‐flow experiment and subsequent disassembly of the reactor, a pronounced structural transformation of the AEH beads was observed (Figure 4E). While initially present as discrete spherical particles, the beads had swollen under operational conditions and reorganized into a cohesive, hydrogel‐like monolith. ESEM analysis (Figure 4F–I) revealed fusion of the individual particles into a continuous matrix while largely preserving the internal macroporous architecture. High‐magnification cross‐sectional images (Figure 4I) show channel‐like features spanning the merged material, suggesting that transport pathways remain accessible despite the macroscopic consolidation of the beads. This flow‐induced restructuring highlights the adaptive nature of the crosslinked protein network: under aqueous operation, the beads expand and progressively eliminate interparticle voids, thereby increasing the effective catalytic volume. Notably, sustained catalytic performance during continuous operation indicates that mass transport through the restructured matrix remains sufficient for substrate and product exchange. Such autonomous in situ consolidation represents an intrinsic process‐intensifying mechanism distinct from conventional carrier‐based systems and illustrates how AEH materials combine structural plasticity with functional stability under flow conditions.

### 2.3 Continuous‐Flow Synthesis of Pseudouridine‐5′‐monophosphate (PMP)

To demonstrate the broader applicability of the AEH bead platform and extend the proof‐of‐concept toward reactions of higher industrial relevance, we employed the beads in the same reactor configuration described above (Figure 4C) for the synthesis of the C‐nucleotide pseudouridine‐5′‐monophosphate (PMP). PMP represents a key intermediate in the production of next‐generation mRNA vaccines and cancer therapeutics, where it serves as a modified nucleotide that enhances mRNA stability and translational efficiency [38]. For a cost‐effective, one‐step biocatalytic route, we selected the trimeric pseudouridine‐5′‐phosphate glycosidase from *E. coli* (EcYeiN, EC 4.2.1.70), which catalyzes the condensation of uracil and ribose‐5‐phosphate to form PMP (Figure 5A) [39]. This enzyme has previously been applied in discontinuous multi‐enzyme cascades for the synthesis of pseudouridine derivatives [40, 41, 42], but its integration into a continuous flow system using carrier‐free immobilized enzymes has not yet been demonstrated.

**FIGURE 5:**
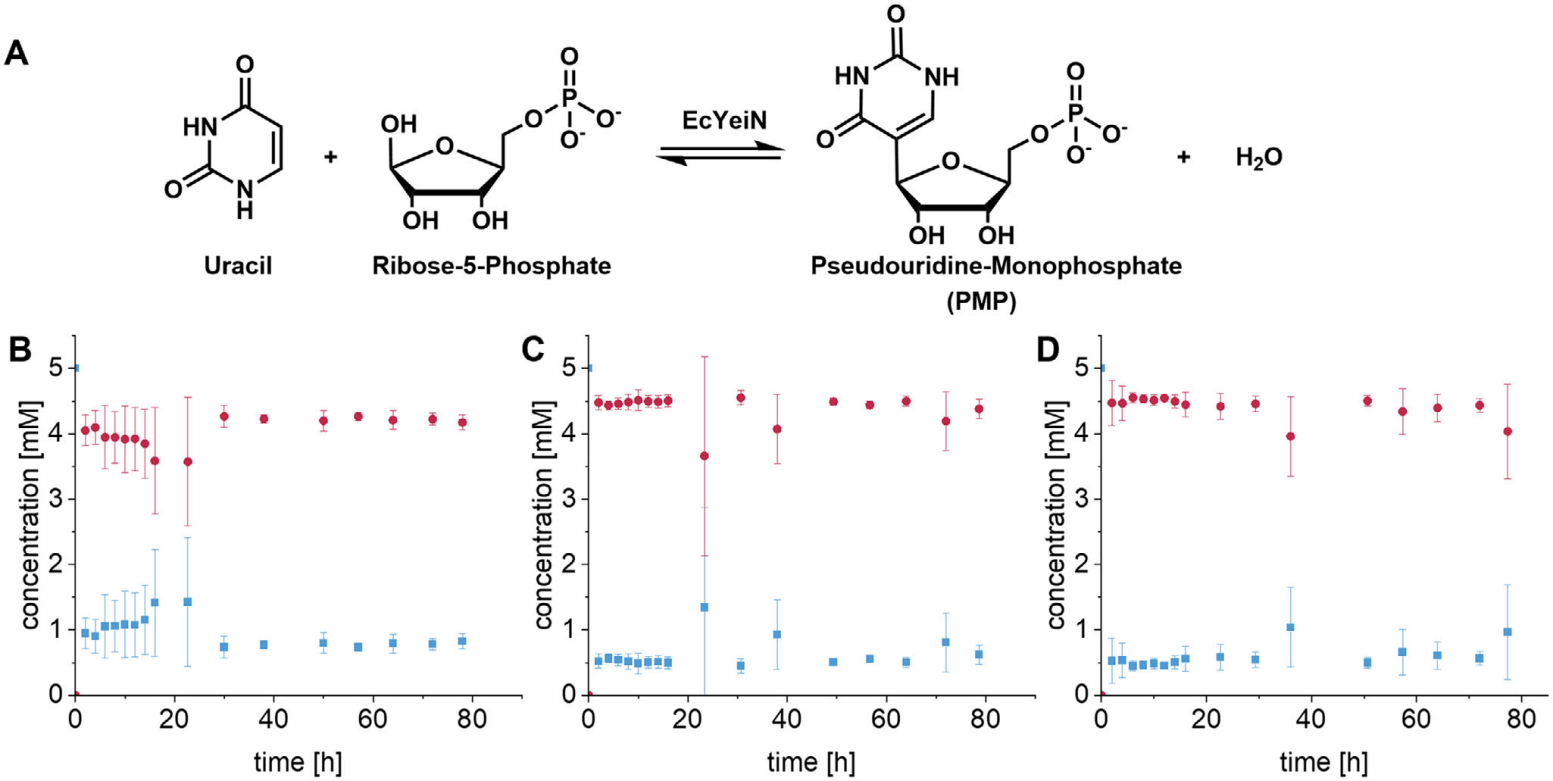
Continuous‐flow synthesis of pseudouridine‐5′‐monophosphate (PMP) using EcYeiN‐based AEH beads. (A) Reaction scheme for the enzymatic condensation of uracil and ribose‐5‐phosphate to pseudouridine‐5′‐monophosphate (PMP) catalyzed by the pseudouridine‐5′‐phosphate glycosidase EcYeiN from *E. coli*. (B–D) Continuous‐flow operation of 100 µL bead‐filled reactors loaded with four EcYeiN AEH beads, showing the concentrations of uracil (blue) and PMP (red) in the reactor outflow at flow rates of 2 (B), 5 (C), and 10 µL min^−1^ (D). Stable product formation and high conversion were maintained over extended operation times. Reaction conditions: 5 mM uracil and 7.5 mM ribose‐5‐phosphate in HEPES buffer (50 mM HEPES, 10 mM MnCl_2_, pH 7.0) at room temperature. All measurements were performed in at least triplicate (*n* ≥ 3) and are shown as mean ± standard deviation.

We first constructed an expression plasmid encoding a His_6_‐tagged variant of EcYeiN and produced the enzyme heterologously in *E. coli*. The protein was purified by Ni–NTA affinity chromatography. To confirm the reported catalytic activity, batch reactions were performed with 6 µM EcYeiN, 50 mM uracil, and 75 mM ribose‐5‐phosphate in HEPES buffer (50 mM HEPES, 10 mM MnCl_2_, pH 7.0) at 37°C for 2 h. Analysis by ^1^H and ^13^C NMR (Figure S11) confirmed the expected formation of pseudouridine‐5′‐monophosphate (PMP), verifying the suitability of EcYeiN for further immobilization experiments.

To enable all‐enzyme hydrogel (AEH) formation, EcYeiN was genetically fused with either an N‐terminal SpyCatcher (SC‐EcYeiN) or SpyTag (ST‐EcYeiN) domain. Both fusion proteins were expressed in *E. coli* and purified by Ni–NTA chromatography (Figure S1, lanes 3 and 4; Table S1). Enzymatic formation of the product PMP from uracil was monitored by HPLC (Figure S12). The catalytic activities of SC‐EcYeiN and ST‐EcYeiN were then compared with that of unmodified EcYeiN under identical reaction conditions (Figure S13). Both fusion variants retained full enzymatic activity and even exhibited slightly increased specific activities of 10.3 ± 1.2 and 9.6 ± 1.2 µmol min^−1 ^mg^−1^ for ST‐EcYeiN and SC‐EcYeiN, respectively, compared with 8.3 ± 1.0 µmol min^−1 ^mg^−1^ for the native enzyme. These results confirm that fusion of the SpyCatcher and SpyTag domains does not impair catalytic performance and may even enhance enzyme stability or substrate accessibility.

To further corroborate the functionality of the coupling domains and the ability of EcYeiN to undergo controlled self‐assembly, crosslinking kinetics between SC‐EcYeiN and ST‐EcYeiN were analyzed by SDS‐PAGE (Figure S14). Equimolar mixtures of both fusion proteins (0.5 mM each) were incubated at 25°C, and samples were collected over 2 h. Approximately 90% of ST‐EcYeiN was covalently linked within 30 min, with near‐complete crosslinking achieved after 1 h, consistent with previously reported kinetics of SpyCatcher/SpyTag‐mediated enzyme assembly [20, 22]. These results confirm efficient covalent ligation between the fusion proteins and demonstrate that EcYeiN retains its programmable self‐assembly capability, thereby providing the molecular basis for the formation of structurally defined AEH bead materials.

After validating the catalytic competence of the individual SC‐ and ST‐EcYeiN fusion proteins, monodisperse EcYeiN‐AEH beads were formulated using the established self‐assembly and droplet‐solidification workflow at a total protein concentration of 1 mM. The resulting dried particles exhibited uniform spherical morphology and narrow size distribution, comparable to the EsPAD‐based materials and other enzyme systems prepared within this study (Figure S15), underscoring the modularity and reproducibility of the bead fabrication strategy. Following lyophilization, four beads (18.2 nmol total enzyme) were packed into 100 µL tubular reactors (Figure 4C) and operated under continuous‐flow conditions at room temperature. The reactors were perfused with uracil and ribose‐5‐phosphate in buffered aqueous solution at flow rates of 2, 5, and 10 µL min^−1^, and product formation was quantified by HPLC analysis of the outflow. Across all tested conditions, the EcYeiN AEH beads maintained high catalytic efficiency, reaching up to 90% conversion of uracil (Figure 5B,C). At a flow rate of 10 µL·min^−1^, the system continuously produced 48 mL of 4.5 mM PMP over 78 h of operation while sustaining stable conversion levels of approximately 90% (Figure 5D). The enhanced conversion observed at higher flow rates is consistent with the reversible nature of the C‐glycosylation reaction: continuous product removal under flow effectively shifts the apparent steady‐state equilibrium toward PMP formation by minimizing product accumulation and suppressing the backward reaction. This behavior highlights how the AEH bead architecture enables stable long‐term operation while leveraging flow‐induced equilibrium displacement, thereby coupling material robustness with process‐level performance under intensified continuous conditions.

### 2.4 Expanding the Scope: Binary Systems, Ternary Cascades, and Modular Bead Coupling

Having established continuous‐flow operation for two mechanistically distinct single‐enzyme systems (EsPAD and EcYeiN), we next sought to explore the architectural versatility of the AEH bead platform beyond individual catalysts. Specifically, we investigated whether the carrier‐free bead format can accommodate increasing levels of enzymatic complexity, ranging from binary cofactor‐dependent assemblies to fully integrated multi‐enzyme cascades and modular combinations of distinct bead populations. As summarized in Figure 6, these studies demonstrate that AEH beads function not only as immobilized catalysts, but as programmable enzymatic building blocks that enable spatially organized cofactor management, cascade integration, and modular reactor design.

**FIGURE 6:**
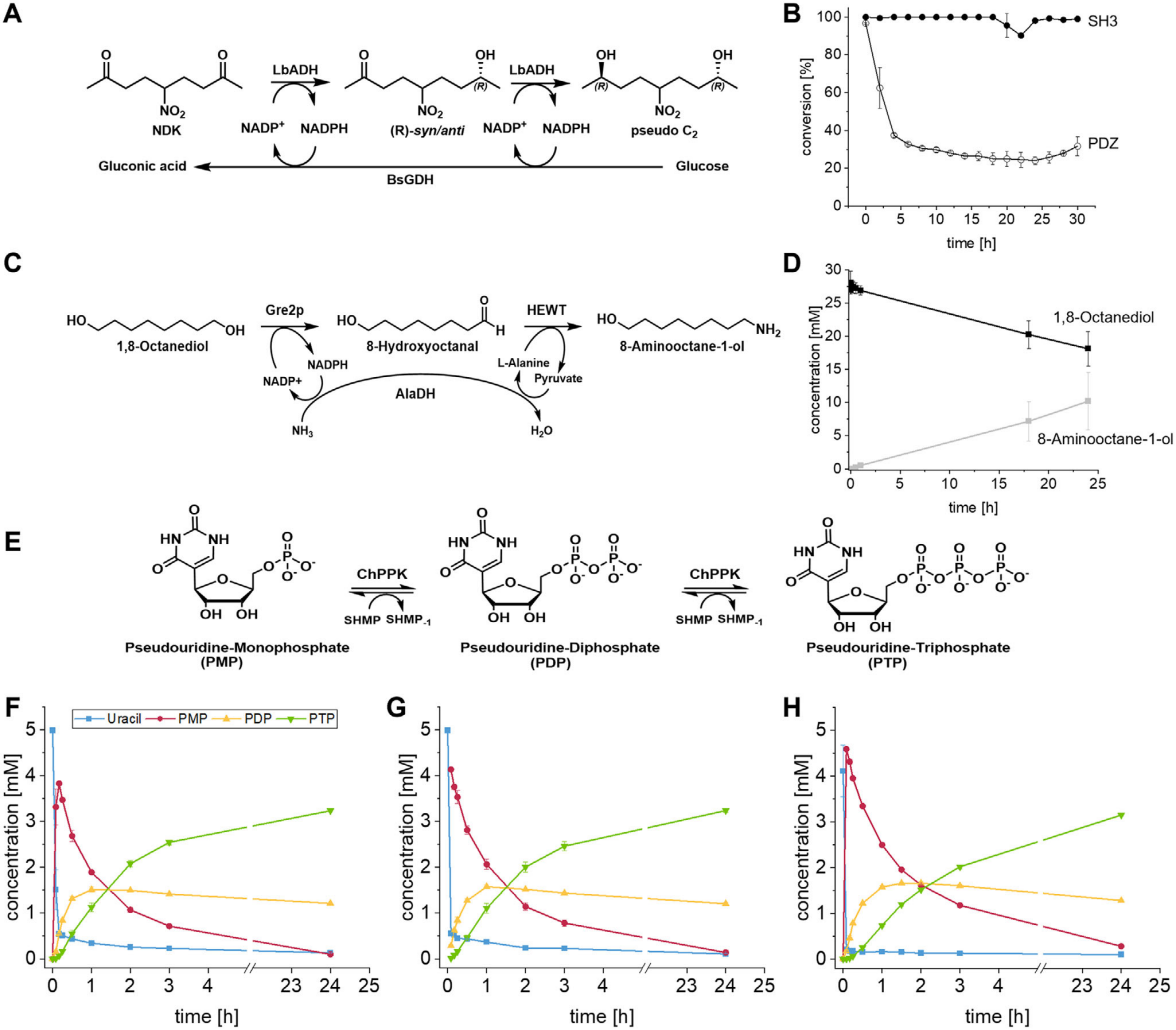
Increasing catalytic complexity and modularity of AEH bead systems. (A) NADP^+^‐dependent two‐step (R)‐selective reduction of 5‐nitrononane‐2,8‐dione (NDK) to the corresponding diol catalyzed by alcohol dehydrogenase (LbADH) with glucose dehydrogenase (BsGDH) for cofactor regeneration. (B) Continuous‐flow conversion of NDK over 30 h using LbADH/BsGDH AEH beads assembled via non‐covalent SH3 (black circles) or PDZ (white circles) interaction modules. (C) Three‐enzyme cascade for amino alcohol synthesis comprising the alcohol dehydrogenase Gre2p, the ω‐transaminase HEWT, and alanine dehydrogenase AlaDH for dual cofactor regeneration. (D) Batch conversion of 1,8‐octanediol (black) to 8‐amino‐1‐octanol (gray) using a single AEH bead containing Gre2p, HEWT, and AlaDH in a defined 1:1:1 ratio assembled via SC/ST interactions (24 h). (E) Enzymatic phosphorylation cascade converting pseudouridine‐5′‐monophosphate (PMP, red) to pseudouridine‐5′‐diphosphate (PDP, yellow) and pseudouridine‐5′‐triphosphate (PTP, green) using polyphosphate kinase (ChPPK) and sodium hexametaphosphate (SHMP) as phosphate donor; PMP is generated from uracil (blue) via EcYeiN (Figure 5). (F–H) Modular bead configurations for sequential nucleotide activation in 1 mL batch reactions: (F) EcYeiN formulated as AEH beads combined with SC‐ChPPK/ST‐EsPAD^d^‐ST beads; (G) free EcYeiN combined with SC‐ChPPK/ST‐EsPAD^d^‐ST beads; (H) free EcYeiN combined with SC‐ChPPK/ST‐ChPPK beads. All experiments were performed in triplicate (*n* = 3).

We first investigated whether the AEH bead format can co‐localize catalysis and cofactor regeneration within a single carrier‐free particle by employing the well‐established NADP^+^‐dependent model system consisting of *Lactobacillus brevis* alcohol dehydrogenase (LbADH) and *Bacillus subtilis* glucose dehydrogenase (BsGDH). This binary system catalyzes the (R)‐selective two‐step reduction of 5‐nitrononane‐2,8‐dione (NDK) to the corresponding diol (Figure 6A) and has previously served as a quantitative benchmark for heterogeneous continuous‐flow biocatalysis formats [43]. In earlier studies, monolithic LbADH/BsGDH all‐enzyme hydrogels assembled via covalent SpyCatcher/SpyTag interactions outperformed conventional immobilization strategies, including adsorption or crosslinking to polystyrene supports, immobilization on magnetic particles, and whole‐cell biofilm catalysts, by up to fivefold in terms of space–time yield (STY) [20, 43]. More recently, foam‐like enzyme materials assembled through either covalent or non‐covalent interaction modules achieved STYs of approximately 180 g L^−1 ^d^−1^ at a flow rate of 10 µL min^−1^ [24, 25].

Building on this benchmarking framework, LbADH/BsGDH AEH beads were formulated using the previously established non‐covalent SH3 and PDZ ligand–binding‐domain (L–B) systems [25]. In both systems, BsGDH was fused to the respective ligand (SH3^L^ or PDZ^L^), whereas LbADH carried the complementary ligand‐binding domain (SH3^B^ or PDZ^B^), enabling multivalent crosslinking of the oligomeric enzyme building blocks (Figure S1A, lanes 8–11, Figures S1C,D and S15). Under comparable flow conditions, the SH3‐based beads achieved STYs of approximately 100 g L^−1 ^d^−1^ and maintained stable NDK conversion over the entire 30 h runtime. In contrast, PDZ‐based beads exhibited an initial decline in conversion within the first 5 h, reaching approximately 30% of the initial activity, after which the system stabilized and continued to operate at a constant level (Figure 6B). Correspondingly, the STY of the PDZ beads was lower (∼2 g L^−1^ d^−1^). The substantially weaker affinity reported for the PDZ L–B interaction (*K*_d_ ≈ 8.0 µM) compared with the SH3 L–B interaction (*K*_d_ ≈ 0.1 µM) [25] may contribute to a lower effective network connectivity and facilitate network restructuring or enzyme loss under continuous‐flow conditions, thereby contributing to the lower catalytic productivity. However, enzyme leaching and changes in effective crosslinking density during operation were not directly quantified here, and the distinct stability profiles therefore cannot be attributed to interaction‐module affinity alone. More generally, these results highlight how the properties of the interaction modules contribute to the structural stability of the resulting protein network. Systematic optimization of assembly parameters, such as interaction‐module affinity, enzyme stoichiometry, and formulation conditions, may therefore provide additional opportunities to further enhance the stability and catalytic performance of AEH bead materials.

Although lower than the ∼180 g L^−1^ d^−1^ previously reported for monolithic foam materials [24, 25], the SH3‐based AEH beads achieved an STY of approximately 100 g L^−1^ d^−1^, corresponding to ∼4.2 g L^−1^ h^−1^. This productivity is below the >16 g L^−1^ h^−1^ benchmark goal illustrated by Wu et al. for an industrially oriented KRED/GDH‐catalyzed ketone reduction [5], but falls within the 1–10 g L^−1^ h^−1^ range proposed by Meissner and Woodley for economically feasible biocatalytic processes targeting high‐priced products [44]. At the same time, it remains below the 100 g L^−1^ h^−1^ boundary used by Bolivar and López‐Gallego to define the high‐activity regime in their benchmarking of heterogeneous continuous‐flow biocatalysts [45]. These values should therefore be regarded as contextual benchmarks rather than universal criteria for economic viability, as appropriate performance targets depend on the specific reaction, product value, catalyst loading, and process configuration. The reduced volumetric productivity relative to the previously reported foam architectures can largely be attributed to the discrete particle geometry, which introduces interparticle void volume and lowers the effective packing density within the reactor bed. The tunable bead dimensions demonstrated here (Figure S4) provide a straightforward parameter for future optimization of reactor packing, whereby bead diameter and size distribution could be adjusted to increase catalyst packing density while balancing mass transport, residence time, and pressure drop.

The distinct stability profiles observed for SH3‐ and PDZ‐based beads further illustrate how the choice of interaction module translates into differences in operational robustness under continuous flow. Complementary batch experiments (Figure S16) revealed coupling‐dependent activation and swelling behavior, with non‐covalent assemblies displaying immediate activity and pronounced hydrogel‐like expansion, whereas covalently crosslinked SC/ST beads exhibited a delayed onset of productivity but retained defined morphology. The SH3‐based beads maintained stable conversion throughout the investigated 30 h flow experiment, remaining operational well beyond the minimum stability period of 5–10 h suggested by Meissner and Woodley as a practical benchmark corresponding approximately to one industrial shift [44]. Since the catalyst was not operated to complete deactivation, however, this experiment defines neither its ultimate operational lifetime nor a total turnover number. Together, these results demonstrate sustained catalytic performance of the bead format while highlighting that productivity and operational stability remain strongly dependent on material architecture and the specific catalytic system.

Having established that binary cofactor‐dependent systems can be integrated and performance‐tuned within discrete AEH beads, we next increased the level of architectural complexity by incorporating a fully coupled three‐enzyme cascade into a single particulate format. This step moves beyond co‐localized cofactor regeneration toward spatially confined multi‐step catalysis with dual cofactor interdependence, thereby probing the capacity of the bead architecture to function as an integrated enzymatic compartment.

To further increase architectural complexity, we evaluated whether a previously reported three‐enzyme cascade for amino alcohol synthesis [46] could be integrated within a single AEH bead architecture. Amino alcohols are valuable chiral building blocks in pharmaceutical synthesis [47], and their biocatalytic production relies on tightly coupled multi‐enzyme networks with internal cofactor recycling. The selected cascade comprises the monomeric alcohol dehydrogenase Gre2p from *Saccharomyces cerevisiae* [48, 49], the dimeric PLP‐dependent ω‐transaminase HEWT from *Halomonas elongata* [50], and the hexameric alanine dehydrogenase AlaDH from *B. subtilis* (Figure 6C) [51]. In the presence of NADP^+^, Gre2p oxidizes 1,8‐octanediol to 8‐hydroxyoctanal, which is subsequently aminated by HEWT using L‐alanine as the amine donor to yield 8‐amino‐1‐octanol. Alanine dehydrogenase closes the internal cofactor cycle by regenerating both NADP^+^ and L‐alanine from NADPH, pyruvate, and ammonia, thereby establishing a self‐sufficient dual cofactor regeneration network within the cascade.

All three enzymes were fused to complementary SpyCatcher/SpyTag domains (Figure S1; lanes 12–15, Table S1) and assembled initially in a defined 1:1:1 ratio into monodisperse AEH beads (Figure 6D and Figure S15), yielding a structurally integrated, carrier‐free multi‐enzyme particle. DLS analysis confirmed nanoscale assembly of the three components and further showed that balancing the overall SC/ST interaction‐module stoichiometry from 2:3 to 3:3 increased the size of the resulting assemblies (Figure S17A), while SDS‐PAGE confirmed the formation of high‐molecular‐weight covalently crosslinked material in the ternary beads (Figure S18A). These results indicate that the differing oligomeric states, and consequently the effective valencies of the interaction modules, influence nanoscale assembly, while still permitting formation of a covalently interconnected ternary enzyme material. Despite the differing oligomeric states and catalytic requirements of the three components, the resulting beads enabled efficient conversion of 1,8‐octanediol to 8‐amino‐1‐octanol in batch operation over 24 h (10.2 mM, 36% yield).

In contrast, the ternary beads showed only limited catalytic performance under continuous‐flow conditions, but recovered substantial activity when subsequently transferred to batch operation (Figure S17B,C). Notably, for the interaction‐module‐balanced 1.5:1:1 formulation, the flow‐recovered beads showed higher subsequent batch conversion than freshly prepared beads, although the limited number of independently tested recovered beads precludes a quantitative assessment of this apparent increase. This behavior suggests that continuous flow may not necessarily favor a spatially confined multi‐enzyme cascade, in which efficient turnover depends on local transfer of intermediates and coupled cofactor recycling between three catalysts within the same bead. While convective flow facilitates external mass transport, it may simultaneously reduce the effective residence and exchange of soluble intermediates and cofactors within the porous enzyme network. The recovery of catalytic activity in batch is consistent with such a transport‐dependent limitation rather than complete irreversible enzyme inactivation. Consistently, high‐molecular‐weight crosslinked material persisted after catalytic application, while the recovered beads remained macroscopically intact despite hydration‐induced transparency and pronounced morphological restructuring during subsequent storage in the hydrated state (Figure S18). These results demonstrate that the AEH bead platform can accommodate heterogeneous enzyme stoichiometries and complex cofactor interdependencies within a single particulate architecture. Conceptually, each bead thus operates as a spatially confined and recoverable catalytic compartment, integrating sequential catalysis and internal cofactor recycling within a single macroscopic enzyme material, while the reduced performance under flow highlights the importance of intraparticle transport for such tightly coupled reaction networks.

Finally, we explored whether distinct catalytic bead populations can be combined in a modular fashion to construct higher‐order reaction sequences at the reactor level. To extend the EcYeiN‐based PMP synthesis toward enzymatic polyphosphorylation, a tetrameric polyphosphate kinase from *Cytohphaga hutchinsonii* (ChPPK) was employed to enable stepwise conversion of pseudouridine‐5′‐monophosphate (PMP) to the corresponding diphosphate (PDP) and triphosphate (PTP) derivatives (Figure 6E; for HPLC analytics, see Figure S19). The purified SC/ST‐ enzyme variants (Figure S1, lanes 16–18) enabled the formulation of monodisperse AEH beads for the different ChPPK‐containing systems (Figure S15), confirming the robustness of the droplet‐based assembly workflow across enzyme classes.

Prior to bead formulation, efficient SC/ST‐mediated crosslinking of the respective ChPPK constructs was verified by SDS–PAGE and densitometric analysis (Figures S20 and S21). In addition to direct SC‐ChPPK/ST‐ChPPK assemblies, we also examined combinations of SC‐ChPPK with ST‐EsPAD^d^‐ST, a catalytically inactive EsPAD variant introduced as an inert structural scaffold within the assembling protein network. This construct was included to assess whether incorporation of an additional scaffold protein can modulate network architecture and thereby influence catalytic performance. Since EsPAD is dimeric and each monomer carries two SpyTag domains, the ST‐EsPAD^d^‐ST construct provides increased valency and therefore requires only half the molar amount relative to ST‐ChPPK to achieve an equivalent degree of crosslinking with SC‐ChPPK. Time‐dependent formation of covalently linked products over a 3 h reaction period confirmed efficient network formation for both construct combinations (Figures S20 and S21).

Based on these validated coupling strategies, ChPPK‐containing AEH beads were formulated and combined with EcYeiN‐based systems in different configurations (Figure 6F–H). In one setup, EcYeiN was immobilized as AEH beads and used together with SC‐ChPPK/ST‐EsPAD^d^‐ST beads (Figure 6F). In alternative configurations, free EcYeiN was combined with either SC‐ChPPK/ST‐EsPADd‐ST beads (Figure 6G) or SC‐ChPPK/ST‐ChPPK beads (Figure 6H). Using 60 mM sodium hexametaphosphate (SHMP) as a phosphate donor together with 5 mM uracil and 7.5 mM ribose‐5‐phosphate for PMP formation by EcYeiN, all three designs yielded approximately 25% PDP and 65% PTP in 1 mL batch reactions after 24 h. Notably, these product distributions were obtained without any reaction optimization and already match the yields reported in recent dedicated studies of enzymatic pseudouridine phosphorylation [42, 52]. The robust formation of both PDP and PTP across all configurations demonstrates that sequential nucleotide activation can be effectively achieved using spatially separated yet functionally integrated bead populations. Importantly, incorporation of the catalytically inactive EsPAD^d^ scaffold did not significantly alter product distribution or overall phosphorylation efficiency, indicating that catalytic performance is primarily governed by substrate accessibility and inter‐bead mass transport rather than by the presence of an additional structural protein within the network.

Across the different enzyme systems investigated here, continuous‐flow applicability and operational stability were found to depend strongly on enzyme composition and network architecture. EcYeiN AEH beads maintained approximately 90% conversion for up to 78 h (Figure 5D), whereas SH3‐assembled LbADH/BsGDH beads retained stable activity over 30 h, while the corresponding PDZ‐based system stabilized only after an initial activity loss (Figure 6B and Figure S16). Extended EsPAD flow experiments at 5 and 10 µL min^−1^ further demonstrated flow‐rate‐dependent conversion, with higher conversion at the lower flow rate, and revealed an initial decline followed by sustained residual conversion over 42 h (Figure S22). ChPPK‐containing systems continuously produced the phosphorylated products PDP and PTP over 24 h across different combinations of free and immobilized EcYeiN and ChPPK‐containing AEH beads (Figure S23). In contrast, the three‐enzyme Gre2p/HEWT/AlaDH cascade beads showed only limited activity under continuous‐flow conditions, although substantial catalytic activity was recovered when the beads were subsequently transferred to batch operation (Figure S17B,C). Thus, while the AEH bead format is broadly compatible with continuous‐flow operation, successful flow performance cannot be inferred from bead formation or batch activity alone. In particular, tightly coupled intrabead cascades may not necessarily benefit from convective flow, as efficient overall turnover requires local transfer of intermediates and cofactors between multiple catalysts confined within the same bead.

These contrasting behaviors indicate that AEH bead performance is governed by several interconnected levels of stability, transport, and process conditions, including intrinsic enzyme stability, interaction‐module affinity, network swelling and restructuring, substrate and cofactor transport, flow rate and residence time, and the specific reaction environment. An additional factor may arise from the intrinsic dynamics of the oligomeric enzyme building blocks themselves. Homo‐oligomeric proteins can exist in dynamic association–dissociation equilibria, with their quaternary structure influenced by environmental conditions and ligand binding [53]. Since AEH network connectivity derives from the multivalency of these oligomeric building blocks, subunit dissociation or rearrangement may decrease effective network connectivity and thereby contribute to enzyme‐dependent differences in bead swelling, morphology, and operational stability. Conversely, stabilization of enzyme oligomerization and optimization of interaction‐module affinity, network architecture, and transport properties provide complementary molecular and process‐level strategies for improving AEH bead stability and continuous‐flow performance. Thus, AEH bead stability should be regarded as a system‐specific and tunable property rather than a universal characteristic of the material format.

This bead–bead coupling strategy establishes AEH particles as programmable catalytic building blocks that can be combined, exchanged, or spatially arranged to tailor reactor functionality. In contrast to monolithic enzyme materials, the discrete bead format enables modular process design, where reaction sequences can be reconfigured by altering bead composition rather than re‐engineering the entire catalytic matrix. Such architectural modularity extends the AEH platform from single‐particle microreactors toward adaptable multi‐compartment catalytic systems capable of sequential and orthogonal transformations.

## 3 Conclusion

In this work, we establish monodisperse all‐enzyme hydrogel (AEH) beads as a structurally programmable, carrier‐free biocatalyst format for continuous‐flow applications. By translating nanoscale self‐assembly of complementary enzyme building blocks into a droplet‐based formulation process, we generate mechanically robust, porous, and geometrically defined protein particles that retain full catalytic competence after cryogenic consolidation and drying. The resulting bead architecture combines the high catalytic density and cofactor retention characteristic of all‐enzyme materials with the handling advantages and reactor compatibility of discrete particulate systems.

Using mechanistically distinct model reactions, we demonstrate the transferability and scalability of this material concept. Cofactor‐independent decarboxylation (EsPAD) and metal‐dependent C–C bond formation (EcYeiN) both proceed efficiently under continuous‐flow conditions, including long‐term operation exceeding 80 h. In biphasic solvent systems, the beads tolerate elevated substrate concentrations, highlighting their structural resilience under intensified reaction conditions. Notably, flow‐induced swelling and particle fusion reveal an adaptive material response that minimizes interparticle voids and maximizes effective catalytic volume during operation, suggesting intrinsic process‐intensifying behavior.

Beyond single‐enzyme systems, the AEH bead platform accommodates increasing levels of catalytic complexity. Binary LbADH/BsGDH assemblies enable spatially organized cofactor regeneration within discrete particles and achieve industrially relevant STYs while maintaining operational stability. Integration of a ternary dehydrogenase/transaminase cascade with dual cofactor recycling demonstrates that heterogeneous enzyme stoichiometries and interconnected reaction networks can be confined within a single bead, effectively functioning as a self‐sufficient enzymatic microreactor. Finally, the modular combination of distinct EcYeiN‐ and ChPPK‐containing bead populations establishes a bead–bead coupling strategy that enables sequential nucleotide activation without re‐engineering the entire catalytic matrix. This architectural modularity elevates AEH particles from immobilized enzymes to programmable catalytic building blocks.

From a process perspective, the present study establishes technical feasibility rather than industrial readiness. Translation of AEH beads toward larger‐scale biocatalytic manufacturing will require scalable particle production and drying, optimization of enzyme production and catalyst loading, reactor packing and pressure drop, as well as application‐specific assessment of operational lifetime and catalyst reuse. The tunability of bead dimensions, composition, and network architecture demonstrated here provides parameters through which these aspects can be addressed, while their economic relevance will ultimately depend on the target reaction, required process performance, and product value.

Collectively, these results position monodisperse AEH beads as a versatile materials platform that bridges enzyme engineering and reactor design. By uniting structural programmability, catalytic density, adaptive behavior under flow, and modular configurability, the bead format expands the design space of carrier‐free biocatalysis and provides a foundation for scalable, reconfigurable continuous processes. Importantly, the genetically encoded and orthogonal assembly strategy enables controlled network formation without the use of external chemical crosslinking reagents and avoids dilution by inert carrier matrices. The resulting shape‐ and size‐defined enzyme particles allow straightforward dosing and integration into both batch and continuous‐flow reactors while maintaining the high catalytic density characteristic of carrier‐free systems. The ability to tailor network architecture, coupling chemistry, and particle geometry thus offers a pathway toward next‐generation biocatalytic materials that combine molecular precision with practical process compatibility.

## Author Contributions

**Jennifer Kühne**: investigation, writing – original draft, writing – review and editing, visualization, validation, methodology, formal analysis, data curation, conceptualization. **Julian S. Hertel**: investigation, writing – original draft, writing – review and editing, visualization, validation, methodology, formal analysis, data curation, conceptualization. **André Delavault**: investigation, writing – original draft, methodology, validation, visualization, writing – review and editing, formal analysis, data curation, conceptualization. **Judith Felk**: investigation, validation, visualization, formal analysis, data curation, methodology, writing – review and editing. **Martin Peng**: writing – review and editing, formal analysis, data curation, visualization, investigation. **Lara Reuber**: investigation, visualization, validation, formal analysis, data curation. **Felix Ott**: methodology, validation, writing – review and editing. **Kim Wanner**: investigation, validation, formal analysis, data curation. **Marc F. Münker**: methodology, writing – review and editing. **Adrian Neukirch**: investigation, visualization, formal analysis, data curation. **Alexei Kiselev**: investigation, methodology, validation, visualization, formal analysis, data curation, writing – review and editing. **Kersten S. Rabe**: conceptualization, writing – original draft, writing – review and editing, supervision, investigation. **Christof M. Niemeyer**: conceptualization, funding acquisition, writing – original draft, writing – review and editing, supervision, investigation.

## Conflicts of Interest

A related patent was filed including JSH, AD, KSR and CMN as inventors.

## Supporting information




**Supporting File**: adma75120‐sup‐0001‐SuppMat.pdf.

## Data Availability

The data that support the findings of this study are available from the corresponding author upon reasonable request.
