# Supplementary material for: Programmable Carrier‐Free All‐Enzyme Beads for Modular Continuous‐Flow Biocatalysis
Source: Adv Mater. 2026 Sep 24;38(62):e75120. doi: 10.1002/adma.75120 (PMC13741103; doi:10.1002/adma.75120)
Supplement: Supplementary file 1 [file ADMA-38-e75120-s001.pdf]

Supporting Information

Programmable Carrier-Free All-Enzyme Beads for Modular Continuous-Flow Biocatalysis

*Jennifer Kühne†, Julian S. Hertel†, André Delavault†, Judith Felk, Martin Peng, Lara Reuber, Felix Ott, Kim Wanner, Marc F. Münker, Adrian Neukirch, Alexei Kiselev, Kersten S. Rabe and Christof M. Niemeyer**

Materials and Methods

Genetic construction of plasmids: The *in vitro* recombination method described by Gibson *et al.* [54] was employed to codon-optimized genes of EcYeiN proteins with a N-terminal His_6-tag and 30 bp homologous overlaps into a pET-22b-based vector (Novagen, Darmstadt, Germany). After the assembly, DpnI digests were carried out, and the reaction mixtures were transformed into chemically competent NEB5-alpha cells (New England Biolabs). The ZR Plasmid Miniprep - Classic kit (Zymo Research) was used for plasmid isolation and purification according to the manufacturer's instructions. The correct assembly of the expression plasmids was verified by commercial sequencing (LGC Genomics, Germany). The used primers were ordered from Sigma Aldrich and inserts were designed and ordered by Invitrogens GeneArt Strings DNA Fragments service of ThermoFisher Scientific. All codon optimized genes of proteins used in this study and used primers are listed in Table S1 and S2.

Protein expression and purification. Protein production was typically carried out using 2 L overnight cultures prepared from individual *E. coli* BL21 (DE3) colonies carrying the respective plasmids. The cultures were harvested by centrifugation (10000 g, 10 min, 4 °C), resuspended in 20 mL NP_i-10 buffer (50 mM NaH_2PO_4, 500 mM NaCl, 10 mM Imidazole, pH 8.0). The cells were lysed by ultrasonication, the cell debris removed by centrifugation (45000 g, 1 h, 4 °C) and the supernatant was filtered through a 0.45 µm Durapore PVDF membrane (Steriflip, Millipore) and loaded on a HisTrap FF (5 mL) Ni-NTA column (GE Healthcare, Germany) mounted on an Äkta Pure liquid chromatography system (GE Healthcare, Germany). After washing with 100 mL NP_i-10 buffer, the His_6-tagged proteins were eluted using a gradient from 100% NP_i-10 buffer to 100% NP_i-500 buffer (50 mM NaH_2PO_4, 500 mM NaCl, 500 mM Imidazole, pH 8.0). Subsequently, the buffer was exchanged to KMB (100 mM KP_i pH 7.5, 1 mM $MgCl_2$) for LbADH and BsGDH variants, to KMB with additional 150 mM NaCl for EsPAD and TRIS buffer (50 mM TRIS-HCl, pH 7) for EcYein and ChPPK enzymes using Vivaspin centrifugal concentrator with a suitable MWCO (GE Healthcare). The purified proteins were analyzed by standard discontinuous SDS-polyacrylamide Laemmli-midi-gels with Coomassie staining and the molecular mass was compared to the PageRuler Prestained (10-180 kDa, Thermo Scientific). The concentrations were determined by UV-Vis spectroscopy, using the theoretical molar extinction coefficients at 280 nm, as calculated by the Geneious version 9.1.3 software. Note that the used pET-vectors contain an Ampicillin resistance and were induced using 0.1 mM IPTG.

AEH bead formulation. A fluidic setup (Figure S3) was employed for the preparation of AEH-monodisperse beads. For each enzyme, a minimum of 160 µL was required. The final bead formulation contained enzymes in a 1:1 ratio of the respecting interaction modules with the exception of the ternary cascade enzymes. Enzyme predilutions were adjusted accordingly; for example, to achieve a final concentration of 1 mM, 0.667 mM ST-EsPAD-ST and 1.333 mM SC-EsPAD were used. For the trienzymatic cascade all enzyme concentrations (SC-Gre2p-SC, ST-HEWT-ST and ST-AlaDH) were adjusted to 1 mM. The final AEH beads contained the three enzymes in a 1:1:1 ratio. For all other bead formulations 1 mM solutions of each enzyme and a 1:1 ratio were used resulting in final enzyme concentrations of 1 mM in the beads. Two 1 mL glass syringes (Hamilton) were filled with the respective enzyme solutions and installed in a Chemyx syringe pump. The flow rate was set to 10 µL min^{-1}. Each syringe was connected via Teflon tubing (inner diameter 0.5 mm) to a four-chambered mixing reactor, allowing simultaneous delivery and mixing of the enzyme solutions. The mixed solution exited the reactor through another Teflon tube (inner diameter 0.5 mm) positioned above a Dewar flask containing liquid nitrogen. A Hamilton capillary (RN NDL, ga26s/19 mm/Pst 3T) was attached to the tube to generate ~10 µL droplets, which were subsequently lyophilized for 72 h.

Dynamic light scattering. For DLS measurements 100 µL of purified protein solution (EsPAD-SC + ST-EsPAD-ST in a 2:1 ratio, 1 mM total protein concentration) were transferred into UV-cuvettes. For the measurements of the two cascade ratios 100 µL of either 1:1:1 SC-Gre2p-SC, ST-HEWT-ST and AlaDH-ST or 1.5:1:1 (corresponding to 1:1 SC:ST) in a total protein concentration of 400 µM were transferred into UV-cuvettes. Loaded cuvettes were placed in a Nano-Series ZetaSizer Nano ZSP (Malvern Instruments, UK) equipped with a He−Ne-Laser (633 nm). Before measurements proteins were centrifuged at 25 °C for 5 min at 10000 rpm. The concentration of the supernatant was measured and set to the needed concentration. Subsequently the average hydrodynamic radius of the protein particles, calculated from the auto-correlated light intensity data using the ZetaSizer software, was measured over a time course of 3 h with data acquisition every 100 sec.

Fracture point measurements. Comparative measurements of the fracture points of native AEH and formulated AEH-beads was performed at 25 °C using an in house developed indentation platform[28] with the following adaptations. A piston with a length of 18 mm and a diameter of 4.3 mm and a sample container with a diameter of 8 mm and a depth of 4 mm were used for the measurements and were performed at a speed of 1 mm·s^{-1}. 120 µL of the unformulated hydrogel was prepared from 1 mM of peptides A and B, which have complementary binding sites, mixed in a stoichiometric ratio (1:1) and gelled directly in the sample container for 30 minutes in a controlled atmosphere (ventilated incubator at 30 °C). The AEH-beads were prepared at similar concentrations and manually centered in the sample container. The measurements were performed until complete penetration or breakage of the respective materials in experimental triplicate determinations (n = 3) and presented as mean ± standard deviation.

Determination of specific surface area (SSA). The specific surface area (SSA) and porosity of beads was determined with the Brunauer–Emmett–Teller (BET) method[55] using the Anton Paar (Quantachrome) Autosorb iQ instrument. The analysis was performed with N2 and Ar gases at their respective boiling temperatures (77 K for N_2 and 87 K for Ar). Prior to measurements, the samples have been outgassed in vacuum for 48 hours at 40°C, followed by 48 h at room temperature.

Gravimetric determination of the porosity. The porosity of the monodisperse AEH-beads was determined gravimetrically using ethanol and water as probe liquids. Measurements were carried out in triplicate for both 1 mM and 2 mM AEH–monodisperse bead formulations. For each measurement, three individual beads were weighed together, corresponding to an average total dry mass of 0.6 ± 0.2 mg for the 1 mM AEH-beads and 1.63 ± 0.05 mg for the 2 mM AEH-beds. The dry mass (m_{dry}) was recorded after storing the beads under vacuum overnight. The beads were then immersed in either ethanol or deionized water and allowed to equilibrate for 5 min at room temperature to ensure complete solvent penetration into the pores. After equilibration, excess surface liquid was gently removed by blotting with lint-free tissue, and the wet mass (m_{wet}) was determined immediately.
The pore volume (V_{pore}) was calculated as:

$$V_{pore} = \frac{m_{wet} - m_{dry}}{\rho_{liquid}}$$

The relative porosity (ε) was then obtained by normalizing the pore volume to the wet bead mass and the density of the probe liquid:

$$\varepsilon\ (\%) = \frac{V_{pore}}{m_{wet}} \times \frac{1}{\rho_{liquid}} \times 100$$

where ρ_{liquid} is the density of the probe liquid (0.998 $g \cdot cm^{-3}$ for water and 0.789 $g \cdot cm^{-3}$ for ethanol at 25 °C).
All measurements were performed in triplicate, and results are reported as mean ± standard deviation for each condition.

Environmental Scanning Electron Microscopy (ESEM). Particle morphology and internal structure of beads was investigated with scanning electron microscopy (SEM, ThermoFisher Scientific, ESEM FEI Quattro S). Intact beads were cut by scalpel into multiple fragments to reveal their internal structure. All samples were prepared on a high-purity adhesive PELCO Image Tabs™ (TedPella). Imaging was performed in background water atmosphere (50 Pa) to avoid coating the samples with conductive material. The chemical composition has been measured with the energy dispersive X-Ray spectroscopy (EDAX Octane Elite EDS system, AMETEK).

Estimation of geometric porosity from ESEM image analysis. The determination of porosity derived from ESEM image analysis was done using the following equation:

$$porosity = \frac{V_{pores}}{Vtotal} = 1 - \frac{6\ x\ m}{\rho\ x\ \pi\ x\ d^3}$$

The mass of one bead m was calculated by weighing 10 beads together, resulting in a mass of 0.634 mg per bead. The diameter d was derived from ESEM image analysis and amounted to (0.205 cm). The density ρ was assumed to be 1 g cm^{-3}.

Characterization of lyophilization process. The temperature during freeze-thawing was monitored using a temperature probe (*Keyestudio DS18B20)* that was fixed vacuum-tight with a septum stopper in a two-necked flask. Data were acquired by a microcontroller with a sampling rate of one measurement per second and saved as CSV files.

Fluorescence Microscopy. Fluorescence microscopy images were taken either by motorized Axio Observer 7 inverted microscope (Zeiss) equipped with a Prime sCMOS camera (Teledyne Photometrics), a Colibri 7 solid-state light source (Zeiss), a VIS-LED illuminator (Zeiss), Definite Focus.2 (Zeiss), and an environmental control system consisting of an XLmulti S1 Dark LS incubator, XL S2 heating unit, and TempModule S (all PECON). The microscope stage was controlled using an SMC 2009 two-axis controller (Märzhäuser Wetzlar). High-resolution confocal images were acquired with an LSM 880 laser scanning microscope (Zeiss). Microscopic images of the beads were acquired using a portable USB digital microscope (*cainda*, 1920x1440P HD).

Enzymatic activity determination. The decarboxylase activity of the EsPAD variants was measured as previously described[25] with minor modifications for the determination of the enzyme activity in AEH beads. In brief, 1 mL of 5 mM p-coumaric acid dissolved in water was incubated at 30 °C with 600 rpm shaking. The reaction was initiated by addition of 5 µL free EsPAD enzyme (0.18 µM total enzyme concentration), 1 standard EsPAD bead (10 µL volume, ~4.5 nmol of enzyme) or 5 small AEH beads (2 µL volume, ~4.5 nmol of enzyme). At defined time points, 100 µL of the reaction were sampled and quenched by mixing with 300 µL of a 2:1 (v/v) mixture of acetonitrile and aqueous 0.1% trifluoroacetic acid. The samples were then centrifuged and the supernatant was transferred to HPLC vials for further analysis, as reported previously.[56] Measurements were run on one biological replicate with $n \geq 2$ technical replicates in parallel. The enzymatic activity of free EcYeiN variants (ST-, SC- and non-tagged EcYeiN) was determined in a batch reaction setup. To that end 6 µM of the respective EcYeiN variant were added to 1 mL of substrate solution (50 mM uracil, 10 mM $MnCl_2$, 75 mM ribose-5-phosphate in HEPES buffer (50 mM HEPES, pH 7)) and shaken at 1000 rpm and 37 °C. At various timepoints 50 µL were sampled and the reaction immediately stopped in 50 µL methanol. The samples were centrifuged at 18.000 g for 10 min, the supernatant was diluted in 200 µL HEPES buffer (50 mM HEPES, pH 7) and analyzed via RP-HPLC. Measurements were performed technical triplicates (n = 3). For the determination of NDK-converting activity of LbADH/BsGDH AEH beads with SH3-, PDZ- or SC/ST-interaction modules, one AEH bead was added to 1 mL of substrate solution (5 mM NDK, 100 mM D(+)-glucose, 1 mM $NADP^+$ in KMB (100 mM potassium phosphate, pH 7.5, 1 mM $MgCl_2$)) and shaken at 600 rpm and 30 °C for 24 h. 50 µL were sampled at

various time points and the reaction immediately stopped by addition of 150 µL ethyl acetate and the NDK conversion determined via NP-HPLC analysis as previously reported.[25]

The production of aminoalcohol with beads formulated from the three cascade enzymes Gre2p, HEWT and AlaDH (1:1:1 ratio or 1.5:1:1 ratio, corresponding to 1:1 ST.SC ratio) was determined by adding one bead to 1.5 mL substrate solution (25 mM 1,8-octanediol, 250 mM L-alanine, 275 mM ammonium acetate, 0.6 mM $NADP^+$ and 0.4 mM PLP in KPi (100 mM potassium phosphate, pH 7.5)) and shaking at 500 rpm for 24 h. At various time points 100 µL were sampled and the reaction immediately quenched in 30 µL 10 M NaOH. The samples were then extracted in MTBE and analyzed via gas chromatography (GC). The specific activity of free AlaDH variants (AlaDH-ST and untagged AlaDH) was measured spectrophotometrically by monitoring the decrease in NADPH absorbance at 340 nm using a Synergy H1 Hybrid Reader (BioTek). Measurements were performed in 96-well plates with a total reaction volume of 100 µl over a period of 2 h at 30 °C. The reaction mixture contained 5 mM pyruvate, 200 mM ammonium acetate, 0.3 mM NADPH and 0.005 µM enzyme in potassium phosphate (KPi) buffer. Prior to substrate addition, the enzyme was preincubated with NADPH for 5 min. The reaction was initiated by substrate addition. For the determination of the phosphorylation activity of SC-ChPPK/ST-ChPPK and SC-ChPPK/ST-PAD^d-ST AEH beads, two beads were added to 1 mL of substrate solution (40 mM $MgCl_2$, 60 mM SHMP, 5 mM uracil and 7.5 mM ribose-5-phosphate in HEPES (50 mM, pH 7)) containing either one SC-EcYeiN/ST-EcYeiN bead or 6 µM of free EcYeiN enzyme for PMP generation. At specific timepoints samples of 50 µL were taken and the reaction immediately stopped by adding 50 µL of methanol. The samples were analyzed via RP-HPLC. All experiments were performed in at least technical triplicates ($n \geq 3$).

Enzymatic activity of EsPAD AEH beads in recycling. The enzymatic activity of EsPAD AEH beads over 5 reaction cycles was determined using the batch assay setup described above. In brief, the reaction was initiated by the addition of 1 AEH bead to 1 mL aqueous p-coumaric acid (5 mM) and carried out at 30 °C and 600 rpm. After one reaction cycle of 30 minutes the reaction solution was taken out and replaced by 1 mL fresh substrate solution. 100 µL samples were taken at the beginning of each reaction cycle as well as after 30 minutes reaction time. These samples were quenched by mixing with 300 µL of a 2:1 (v/v) mixture of acetonitrile and aqueous 0.1% trifluoroacetic acid. The samples were then centrifuged, and the supernatant was transferred to HPLC vials for further analysis.

Flow experiments. After drying, the resulting EsPAD- or EcYeiN-AEH beads were loaded into 100 µL reactors previously developed by us,[36] and operated at room temperature. Each reactor contained four beads of 10 µL volume or 20 beads of 2 µL volume respectively, corresponding to a total enzyme amount of 18.2 nmol. With EsPAD-AEH beads the reactors were perfused with substrate solution containing 100 mM of *p*-coumaric acid in MTBE with 5% (v/v) of water at a constant flow rate of 15 µL min^{-1}. Samples were collected manually at regular intervals and prepared for

HPLC analysis by mixing 10 µL of sample with 990 µL of stop solution composed of acetonitrile:water (1:1) with 0.1% trifluoroacetic acid.
For the EsPAD AEH Beads in an aqueous flow reaction setup, the substrate solution contained 5 mM para-coumaric acid in H_2O and was pumped through the reactors with a flow rate of 5 and 10 µL min^{-1}. Samples were automatically collected at regular intervals using a customized rotAXYS 360 system (CETONI, Germany), which directed 50 µL of the reactor outflow directly into 150 µL of stopping solution (propylene glycol : H2O + 0.1% TFA in a 2:1 ratio) for immediate quenching. 150 µL of these quenched samples were mixed with 150 µL acetonitrile, centrifuged at 16.000 x g for 10 min and afterwards 200 µL were transferred into HPLC Vials for analysis.
For EcYeiN AEH beads, the reactors were perfused with a substrate solution (5 mM uracil, 7.5 mM ribose-5-phosphate in 50 mM HEPES pH 7, 10 mM $MnCl_2$) and operated under continuous-flow at constant flow rates of 2, 5, and 10 µL min^{-1}. Samples were automatically collected at regular intervals using a customized rotAXYS 360 system (CETONI, Germany), which directed 50 µL of the reactor outflow directly into 50 µL of methanol for immediate quenching. The samples were analyzed by HPLC to quantify the formation of pseudouridine-5′-monophosphate (PMP). Using a similar reaction setup, the NDK-conversion of LbADH/BsGDH beads modified with either SH3- or PDZ interaction modules was determined under continuous-flow conditions. Each reactor was filled with four beads (18.2 nmol of enzyme) and perfused with substrate solution (5 mM NDK, 100 mM D(+)-glucose and 1 mM $NADP^+$ in KMB (100 mM potassium phosphate, pH 7.5, 1 mM $MgCl_2$)) at a flow rate of 5 µL min^{-1} for a total of 30 h. Samples of 150 µL were fractioned automatically at set timepoints using a the same rotAXYS 360 system (CETONI, Germany) into a 96-nunc-well plate und the reaction immediately stopped in 50 µL 7 M CH_5N_3-HCl. 50 µL of these samples were extracted in 150 µL ethyl acetate, dried overnight in a 96-nunc-well plate and analyzed via NP HPLC.
In a similar reaction setup, the catalytic activity under continuous flow of beads made of the ternary enzyme cascade (Gre2p, AlaDH, HEWT) was determined. The substrate solution contained 25 mM of 1,8-octanediol and was pumped through the reactors with a flow rate of 5 µL min^{-1}. This experiment was setup in triplicate, using four AEH beads per reactor. After defined time points, 100 µL outflow were collected directly into 30 µL 10 M NaOH for quenching. 100 µL of the samples were extracted in 400 µL MTBE and 100 µL of the organic phase was analyzed via GC.
For the determination of phosphorylation activity of ChPPK AEH beads under continuous-flow conditions, reactors containing four AEH beads were perfused by substrate solution (40 mM $MgCl_2$, 60 mM SHMP, 5 mM uracil and 7.5 mM ribose-5-phosphate in HEPES (50 mM, pH 7)) using a flow rate of 5 µL min^{-1}. For PMP generation either 6 µM free EcYeiN was added in the substrate solution, or one EcYeiN AEH bead was added to the reactor, in which case only three ChPPK beads were used. At specific timepoints samples of 50 µL were taken automatically, using the setup described above and the reaction immediately stopped in 50 µL of methanol in a 96-well-plate. The methanol was evaporated and 100 µL of HEPES

buffer (50 mM, pH7) were added to the wells. 100 µL of the resuspended samples were transferred into HPLC vials and analyzed via RP-HPLC.

NMR characterization of pseudouridine-monophosphate. Pseudouridine-monophosphate was produced by EcYeiN reaction, as described above. After stopping the reaction, the quenched samples were centrifuged at 18,000 × g for 10 minutes to remove precipitated protein. The remaining organic solvent was reduced in volume using a vacuum concentrator, and Mn^{2+} ions were removed through ion exchange with Amberlite™ IRC120 in the H-form. To this end, the ion exchanger was added directly to the concentrated reaction mixture in a 5 mL microcentrifuge tube and mixed for 2 h. This crude product was then further purified via flash chromatography using a C18 column (FP SELECT C18, 20–35 µm, 4 g) with water as the mobile phase. Finally, the solvent was removed by lyophilization overnight. The isolated PMP appeared as a white, crystalline powder and dissolved in D_2O for ^1H and ^{13}C NMR (see Figure S6).

Detection of 4-vinylphenol by RP-HPLC. The formation of 4-vinylphenol by EsPAD was verified using RP-HPLC, as described previously.[56] In brief, 4-vinylphenol and p-coumaric acid were quantified by a reverse phase HPLC (Eclipse XDC-C18 column, 5 µm Agilent, with precolumn). The separation was realized using a isocratic flow of 1 mL min^{-1} using a mobile phase of 50% acetonitrile and 50 % aqueous 0.1% trifluoroacetic acid at 22 °C for 10 min. The analytes were detected at 285 nm and 260 nm with the following retention times: t_R (p-coumaric acid, A285) = 1.7-1.9 min and t_R (4-vinylphenol, A260) = 1.7-1.8 min. The conversion of p-coumaric acid was determined via the depletion of substrate.

Detection of pseudouridine derivatives via RP-HPLC. The analysis of the EcYein reaction a modified, previously described HPLC method to detect uracil and PMP was applied.[57] In short, the reversed-phase HPLC setup used a C18 column (ZORBAX Eclipse XDB, 5 µm, 150 × 4.6 mm) and 2-morpholin-4-ylethanesulfonic acid (MES) buffer (20 mM MES, 40 mM tetra-n-butylammonium bromide (TBAB), pH 5.9, 12.5% acetonitrile) as a mobile phase. The system was run isocratically at 35 °C, with a flow rate of 1 mL/min for 5 minutes. The quenched samples were evaporated overnight and resuspended in 150 µL of HEPES buffer (50 mM HEPES, pH 7), of which 45 µL were injected for analysis. The analysis of the phosphorylation products from the following ChPPK reaction (PDP, PTP) used the same HPLC setup, but KPi (100 mM potassium phosphate, 10 mM TBAB, pH 6.4 and 7.5% acetonitrile) as a mobile phase, an injection volume of 25 µL and the method was run isocratically at 22 °C with a flow rate of 1.3 mL min^{-1} for 10 min. The analytes were detected at 260 nm with the following retention times: t_R (uracil) = 1.3-1.5 min, t_R (PMP) = 1.7-1.9 min, t_R (PDP) = 2.6-2.9 min and t_R (PTP) = 3.9-4.4 min.

Detection of NDK and conversion products by NP-HPLC. The detection of LbADH/BsGDH reaction products was performed as described previously.[24, 25] A normal phase HPLC system (Agilent 1260 series HPLC) equipped with a Lux 3µm

Cellulose-1 (150x2.00 mm) chiral column (Phenomenex) and a Diode Array Detector was used. The HPLC analysis was done using 40 µL of the extracted samples from the batch reactions were transferred into a 96-nunc-well plate and evaporated overnight. The dried extractions were resuspended in 100 µL of mobile phase (90:10 n-heptane:2-propanol) and injected into the HPLC with a volume of 10 µL. The separation followed an isocratic flow of mobile phase at 10 °C and a flow rate of 0.5 mL min^{-1}. The sample preparation of the samples from the flow experiment was similar with small alterations. 50 µL of the quenched samples of the flow reactions were extracted using 150 µL ethyl acetate and 40 µL of the organic phase transferred into a 96-nunc-well plate and evaporated overnight. All other experimental steps were done as described above.

Detection of aminoalcohol and cascade byproducts via GC. After stopping the reaction with 10 M NaOH, the samples were extracted using 400 µL methyl-tert-butyether (MTBE). 100 µL of the organic phase were analyzed via gas chromotagraphy using a 8890 GC system (Agilent Technologies) equipped with a J&W HP-5 column (30m x 0.32 mm x 0.25 µm) and flame ionization detector. The following method parameters were used: injection volume 1 µL, inlet temperature 250 °C, hydrogen carrier-gas flow 2 mL min^{-1}, column oven temperature gradient 100 °C for 2 min then 7.5 °C min-1 until 132 °C, 40 °C min-1 until 300 °C, hold 1min, temperature of FID 350 °C.

Supplementary Figures

Figure S1: Coomassie-stained SDS-PAGE analysis of recombinantly expressed enzymes used in this study and schemes of interaction domains.
Figure S2: Verification of ST-EsPAD-ST and EsPAD-SC crosslinking.
Figure S3: Droplet-based bead-making platform .
Figure S4: Lyophilized EsPAD-SC and ST-EsPAD-ST AEH beads after formation and size distribution of beads, activity of smaller beads under batch and flow conditions.
Figure S5: Argon- and nitrogen-based adsorption-desorption isotherms of the AEH bead.
Figure S6: Temperature profiles during lyophilization of cryogenically frozen AEH beads.
Figure S7: SDS-PAGE analysis of EsPAD crosslinking during AEH bead fabrication and lyophilization.
Figure S8: Influence of lyophilization pressure on AEH bead morphology and catalytic activity.
Figure S9: Fluorescence microscopy analysis of the spatial protein distribution within EsPAD AEH beads.
Figure S10: Reusability and morphological changes of EsPAD AEH beads during repeated batch catalysis.
Figure S11: Nuclear magnetic resonance spectroscopic assessment of the PMP produced from uracil and ribose-5-phosphat by EcYeiN enzyme.
Figure S12: HPLC chromatograms of uracil and reaction products formed by EcYeiN.
Figure S13: Activity comparison of native and SpyTag/SpyCatcher-modified EcYeiN variants.
Figure S14: Verification of ST-EcYeiN and SC-EcYeiN crosslinking.
Figure S15: Representative AEH beads generated from different enzyme systems.
Figure S16: Stability and activity development of LbADH/BsGDH AEH beads assembled via different coupling systems under batch conditions.
Figure S17: Influence of interaction-module stoichiometry on assembly and catalytic performance of ternary cascade AEH beads.
Figure S18: Structural and morphological characterization of ternary cascade AEH beads before and after application.
Figure S19: Representative HPLC chromatogram of PMP, PDP, PTP separation.
Figure S20: Verification of ST-ChPPK and SC-ChPPK crosslinking.
Figure S21: Verification of ST-EsPAD^{d}-ST and SC-ChPPK crosslinking.
Figure S22: Long-term operational stability of EsPAD AEH beads under continuous-flow conditions.
Figure S23: Operational performance of different ChPPK-containing AEH bead configurations under continuous-flow conditions.

Supplementary Tables

Table S1:	Codon optimized genes of proteins used in this study.
Table S2:	List of used primers and their corresponding sequences.
Table S3:	Comparison of specific activities of native and genetically modified enzyme variants used for AEH bead formation.

Supplementary figures

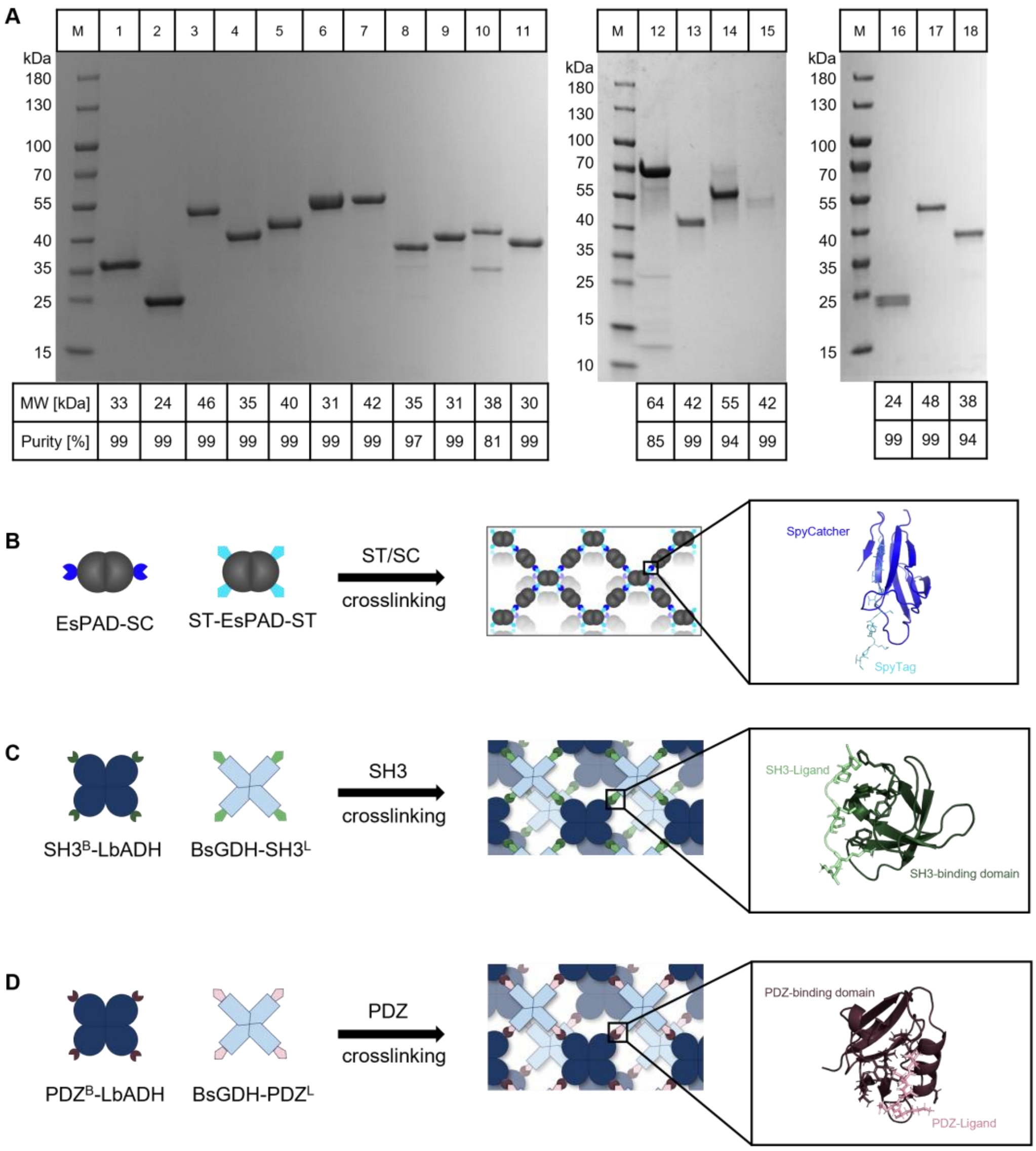

Figure S1. Protein constructs and interaction modules used for AEH material formation. (A) Coomassie-stained SDS-PAGE analysis of the recombinantly expressed enzyme constructs used in this study: EsPAD-SC (lane 1), ST-EsPAD-ST (lane 2), SC-EcYeiN (lane 3), ST-EcYeiN (lane 4), SC-LbADH (lane 5), ST-BsGDH (lane 6), SC-BsGDH (lane 7), $SH3^B$-LbADH (lane 8), BsGDH-$SH3^L$ (lane 9), PDZ^B-LbADH (lane 10), BsGDH-PDZ^L (lane 11), SC-Gre2p-SC (lane 12), ST-Gre2p-ST (lane 13), ST-HEWT-ST (lane 14), AlaDH-ST (lane 15), ST-EsPADd-ST (lane 16), SC-ChPPK (lane 17), and ST-ChPPK (lane 18). Calculated molecular weights and determined protein purities are indicated below the gels. M, prestained protein ladder (10–180 kDa). (B–D) Schematic representation of the interaction modules employed for AEH network formation. (B) Covalent crosslinking via spontaneous reaction between SpyCatcher (SC) and SpyTag (ST), illustrated for EsPAD-SC and ST-EsPAD-ST. (C) Non-covalent crosslinking through interaction of the SH3-binding domain ($SH3^B$) with its cognate SH3 ligand ($SH3^L$), illustrated for $SH3^B$-LbADH and BsGDH-$SH3^L$. (D) Non-covalent crosslinking through interaction of the PDZ-binding domain (PDZ^B) with its cognate PDZ ligand (PDZ^L), illustrated for PDZ^B-LbADH and BsGDH-PDZ^L. B denotes the respective binding domain and L the cognate peptide ligand. Structural representations of the respective interaction pairs are shown in the enlarged views.

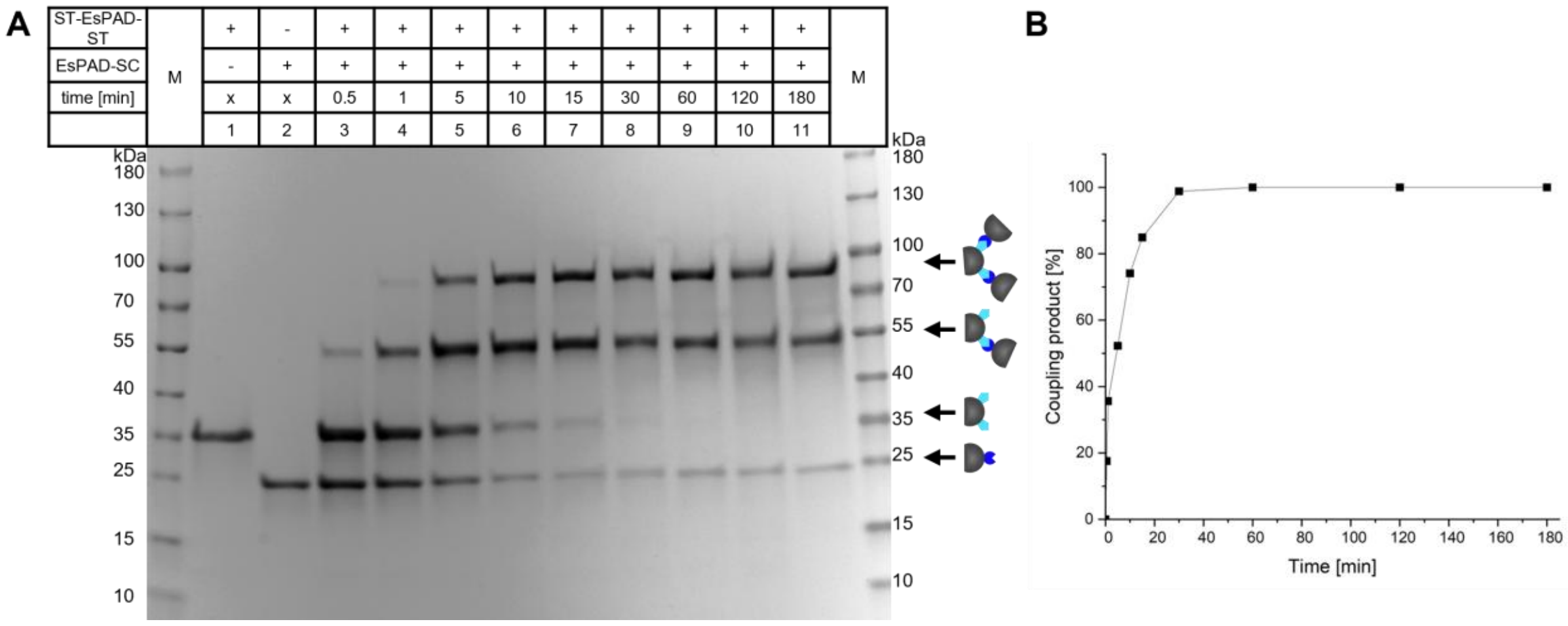

Figure S2. Verification of ST-EsPAD-ST and EsPAD-SC crosslinking. (A) Coomassie stained SDS-PAGE analysis of the purified enzyme variants of EsPAD fused to the covalent interaction systems SC/ST. Lane 1: EsPAD-SC (32.5 kDa); lane 2: ST-EsPAD-ST (23.6 kDa); and verification of ST-EsPAD-ST and EsPAD-SC coupling (lane 3 to 12). M: Prestained Protein ladder (10-180 kDa). (B) Densitometric quantification of cross-linked product formation over a 3-hour reaction period.

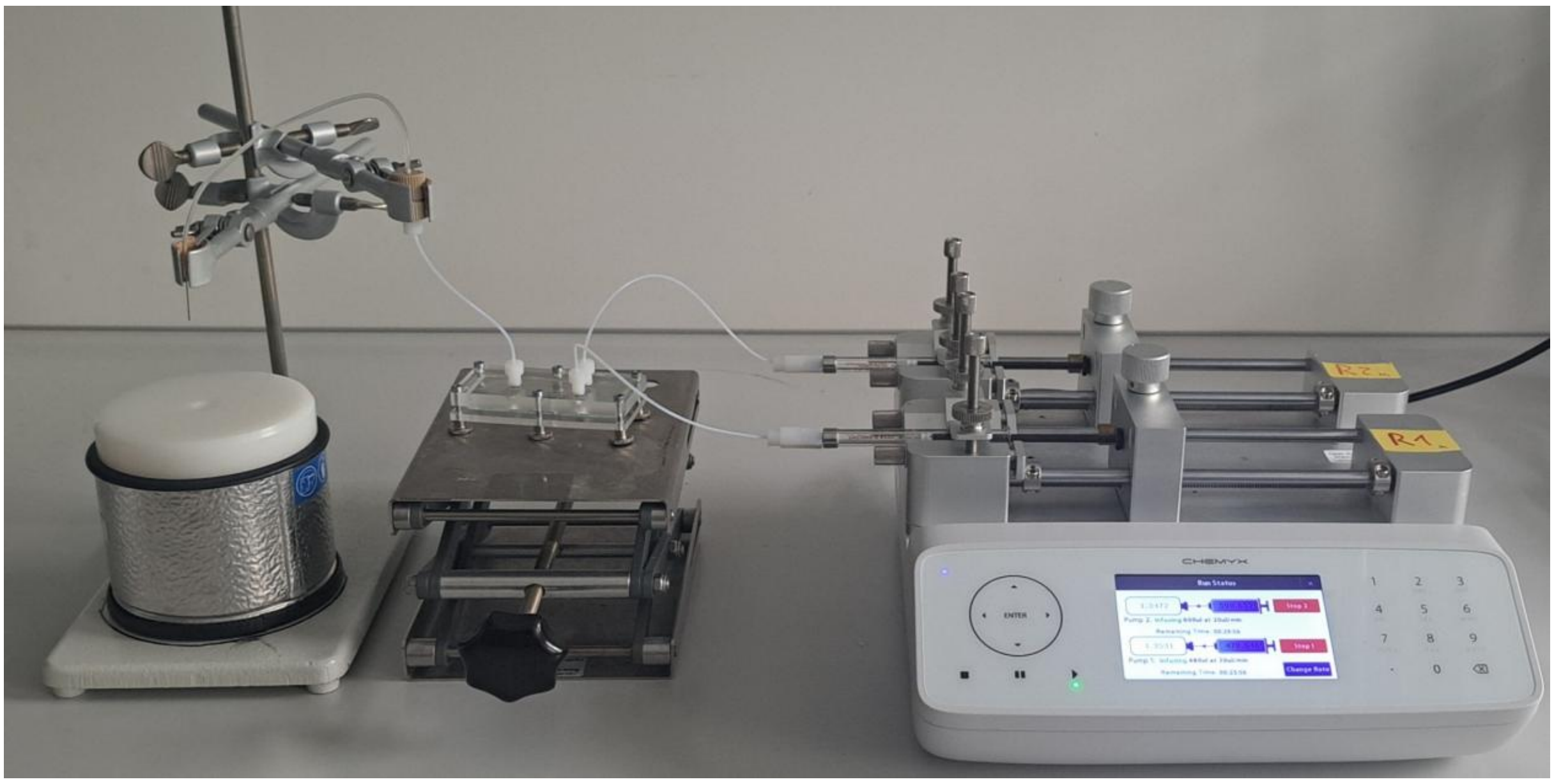

Figure S3. Photograph of the droplet-based bead-making platform, consisting of syringe pumps, a microfluidic four-chambered mixer and a dewar vessel for liquid nitrogen.

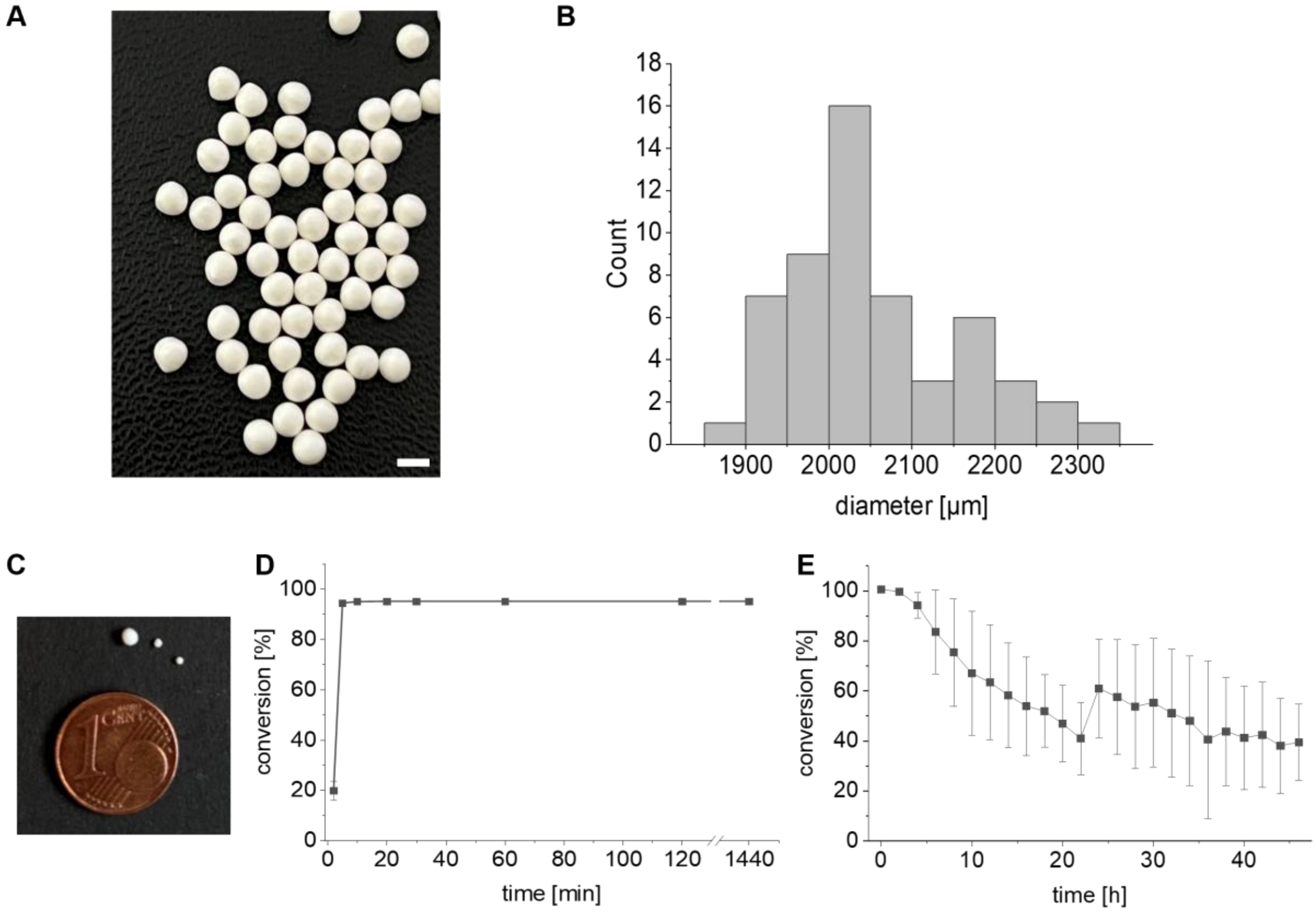

Figure S4. Morphology, size tunability, and catalytic performance of EsPAD AEH beads. (A) Photograph of spherical AEH beads obtained after droplet formation, cryogenic solidification, and lyophilization from a mixture of purified EsPAD-SC and ST-EsPAD-ST (1 mM total protein concentration, 2:1 molar ratio). (B) Size distribution of the resulting standard beads determined by image analysis using ImageJ. Scale bar: 2 mm. (C) Photograph comparing a standard AEH bead prepared from a 10 µL droplet with two size-reduced beads prepared from 2 µL droplets; a 1-cent coin is shown for size reference. (D) Batch conversion of p-coumaric acid (5 mM in H_2O) using five 2 µL EsPAD AEH beads in a total reaction volume of 1 mL over 24 h. (E) Long-term continuous-flow performance of 2 µL EsPAD AEH beads over 46 h. Twenty 2 µL beads were used per reactor, corresponding to the same total initial enzyme loading as four standard 10 µL beads used in the conventional reactor configuration (about 18.2 nmol EsPAD). Batch and continuous-flow experiments were performed in triplicate; data are presented as mean ± standard deviation.

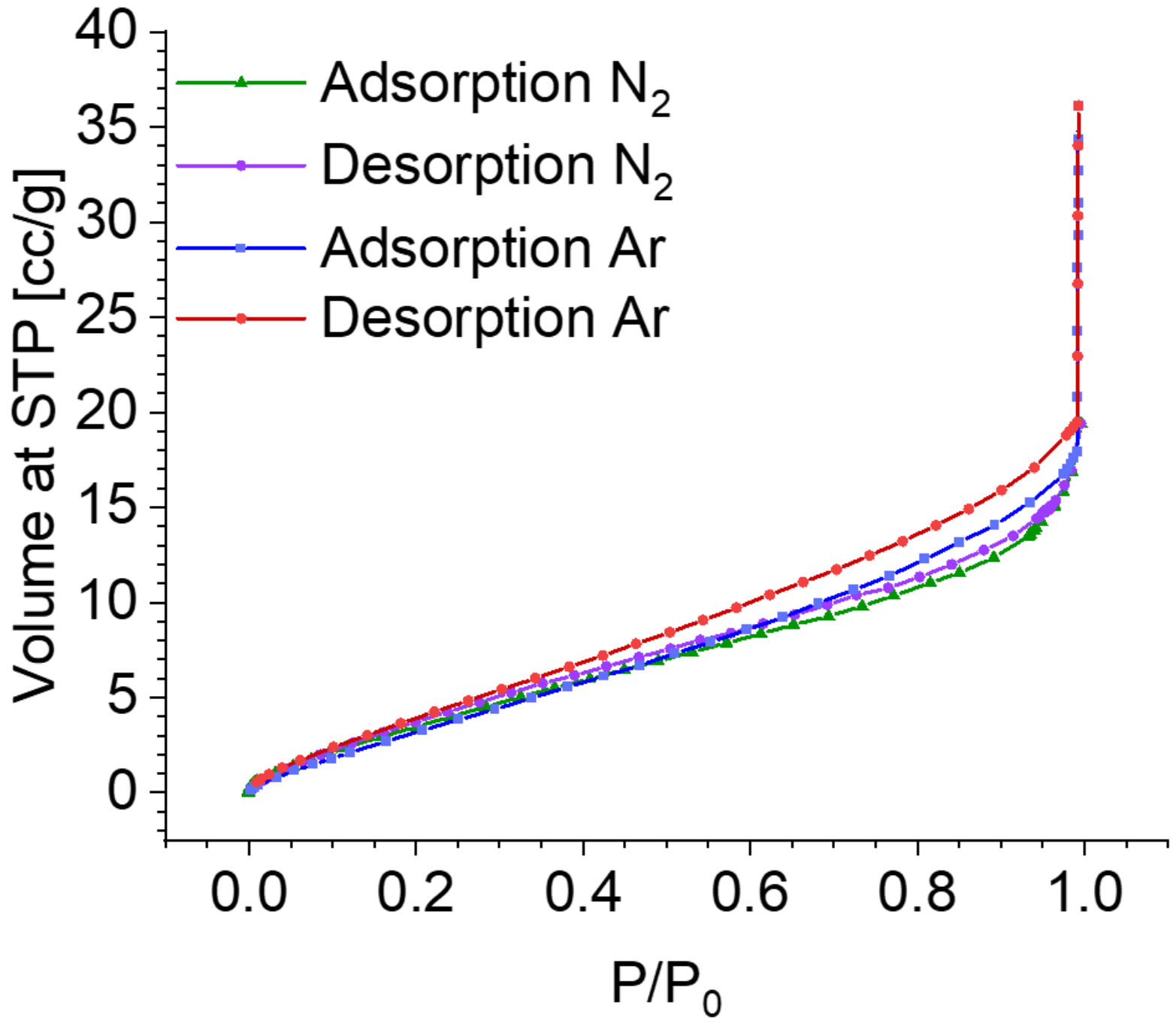

Figure S5. Adsorption–desorption isotherms of AEH beads measured with argon and nitrogen. Full argon (87 K) and nitrogen (77 K) isotherms of AEH-bead samples used to determine the specific surface area and assess porosity characteristics. The two gases yield nearly identical isotherms, confirming the reproducibility and reliability of the measurements. The absence of a pronounced uptake at low relative pressures (P/P_0) and the lack of hysteresis indicate that no nanoporous features are present in the material.

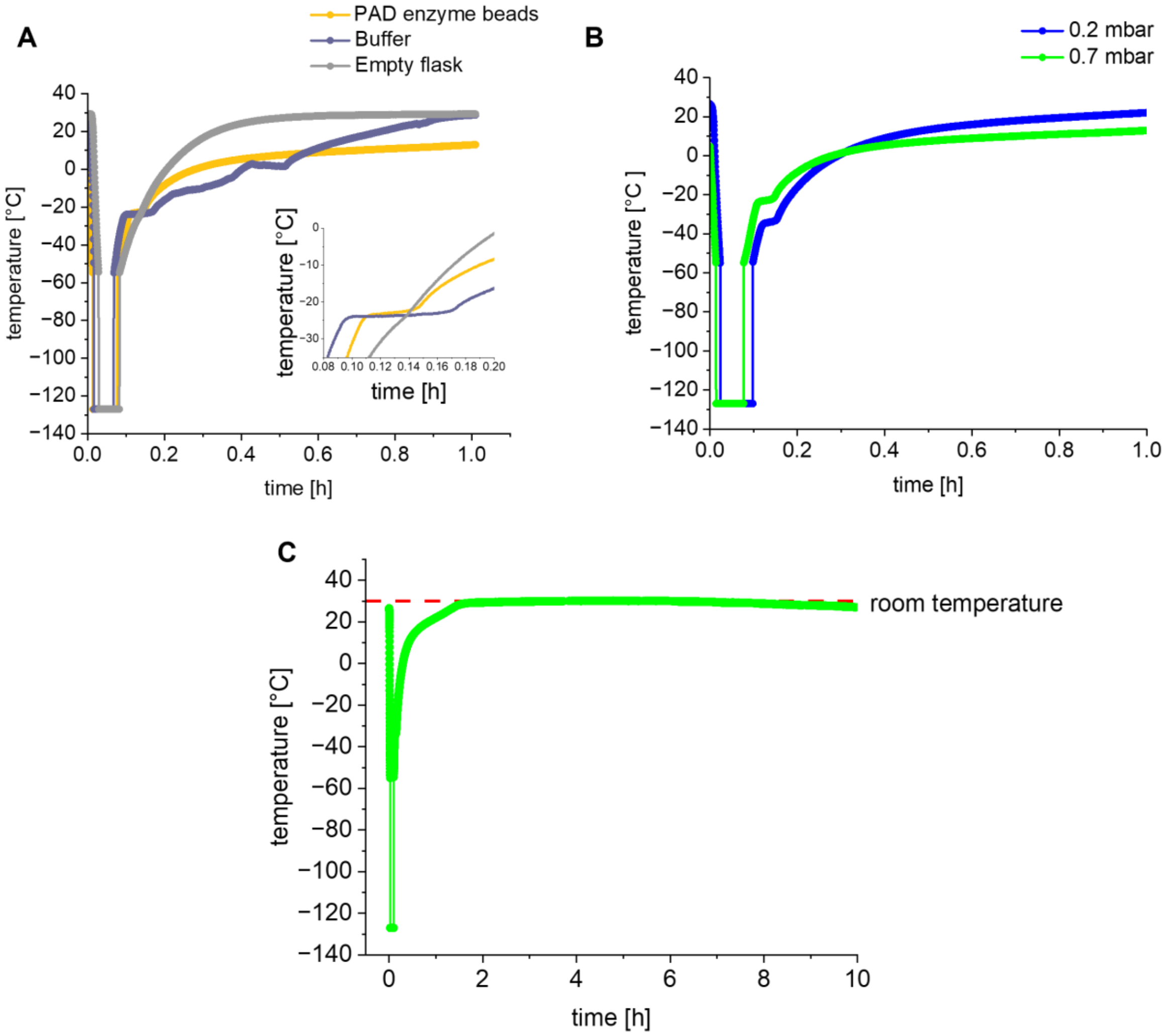

Figure S6. Temperature profiles during lyophilization of cryogenically frozen AEH beads. (A) Temperature profiles recorded during lyophilization of EsPAD AEH beads (yellow), potassium phosphate buffer (KPi, 10 mM, pH 7.6; blue), and an empty flask (grey). Samples were frozen in liquid nitrogen prior to lyophilization. Temperature was monitored using a probe introduced through a second neck of the flask and positioned in direct contact with the inner glass surface at the bottom of the flask. The inset shows an enlarged view of the temperature profiles during the initial phase of primary drying, including the plateau associated with ice sublimation. EsPAD AEH beads with a total initial sample volume of approximately 200 µL exhibited a sublimation plateau of approximately 1.8 min, whereas 500 µL of buffer showed a longer plateau of approximately 4.2 min. No distinct sublimation plateau was observed for the empty flask. (B) Temperature profiles recorded during lyophilization of EsPAD AEH beads at chamber pressures of 0.2 mbar (blue) and 0.7 mbar (green). The lower chamber pressure resulted in a lower temperature during the sublimation phase, demonstrating the pressure dependence of the thermal profile during primary drying. (C) Temperature profile recorded over the complete 10 h lyophilization process of EsPAD AEH beads at a chamber pressure of 0.7 mbar. Following the initial low-temperature phase, the measured temperature gradually increased and reached approximately room temperature after about 3 h, remaining close to this level for the remainder of the drying process. Minor subsequent temperature variations are consistent with fluctuations in ambient temperature.

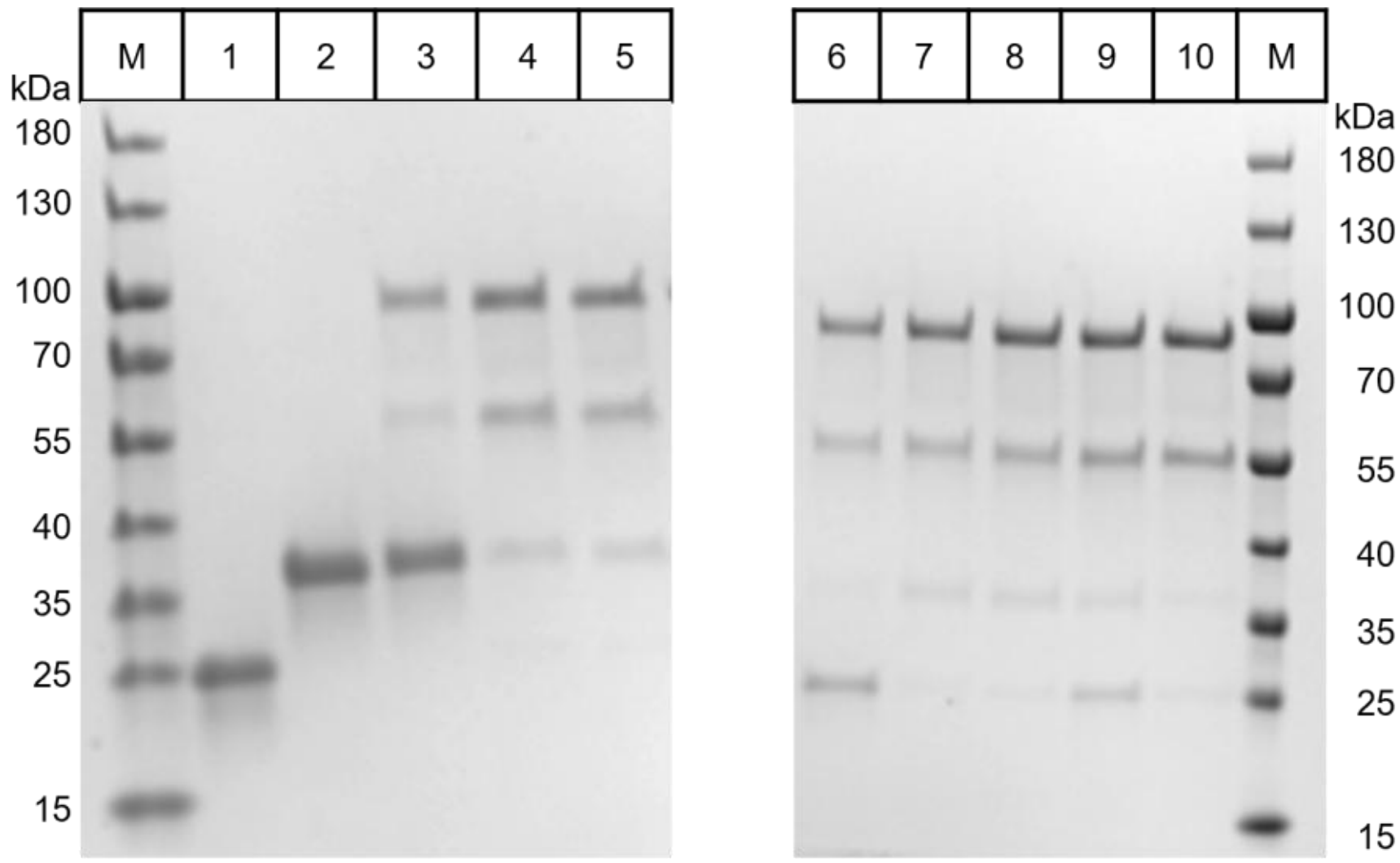

Figure S7. SDS-PAGE analysis of EsPAD crosslinking during AEH bead fabrication and lyophilization. Coomassie-stained SDS-PAGE showing ST-EsPAD-ST (lane 1) and EsPAD-SC (lane 2) individually, followed by mixtures of ST-EsPAD-ST and EsPAD-SC sampled at successive stages of AEH bead preparation. Samples were analyzed immediately after mixing (lane 3), after microfluidic mixing and prior to cryogenic solidification (lane 4), and directly after recovery from liquid nitrogen (lane 5). Samples collected during subsequent lyophilization were analyzed at the sublimation plateau (lane 6) and after the sample temperature reached 0 °C (lane 7), 10 °C (lane 8), and 20 °C (lane 9), as well as after completion of lyophilization for 3 days (lane 10). Progressive formation of higher-molecular-weight species is consistent with continued SC/ST-mediated covalent crosslinking during bead fabrication and lyophilization. Note that the residual uncrosslinked ST-EsPAD-ST band shows some variation in intensity between individual lyophilization time points, most prominently at 20 °C (lane 9). Such variation may arise from heterogeneity of the partially dried material and differences in sample recovery, solubilization, or loading during SDS-PAGE sample preparation and is therefore not interpreted as a change in the covalent SC/ST linkage. M, PageRuler Prestained Protein Ladder.

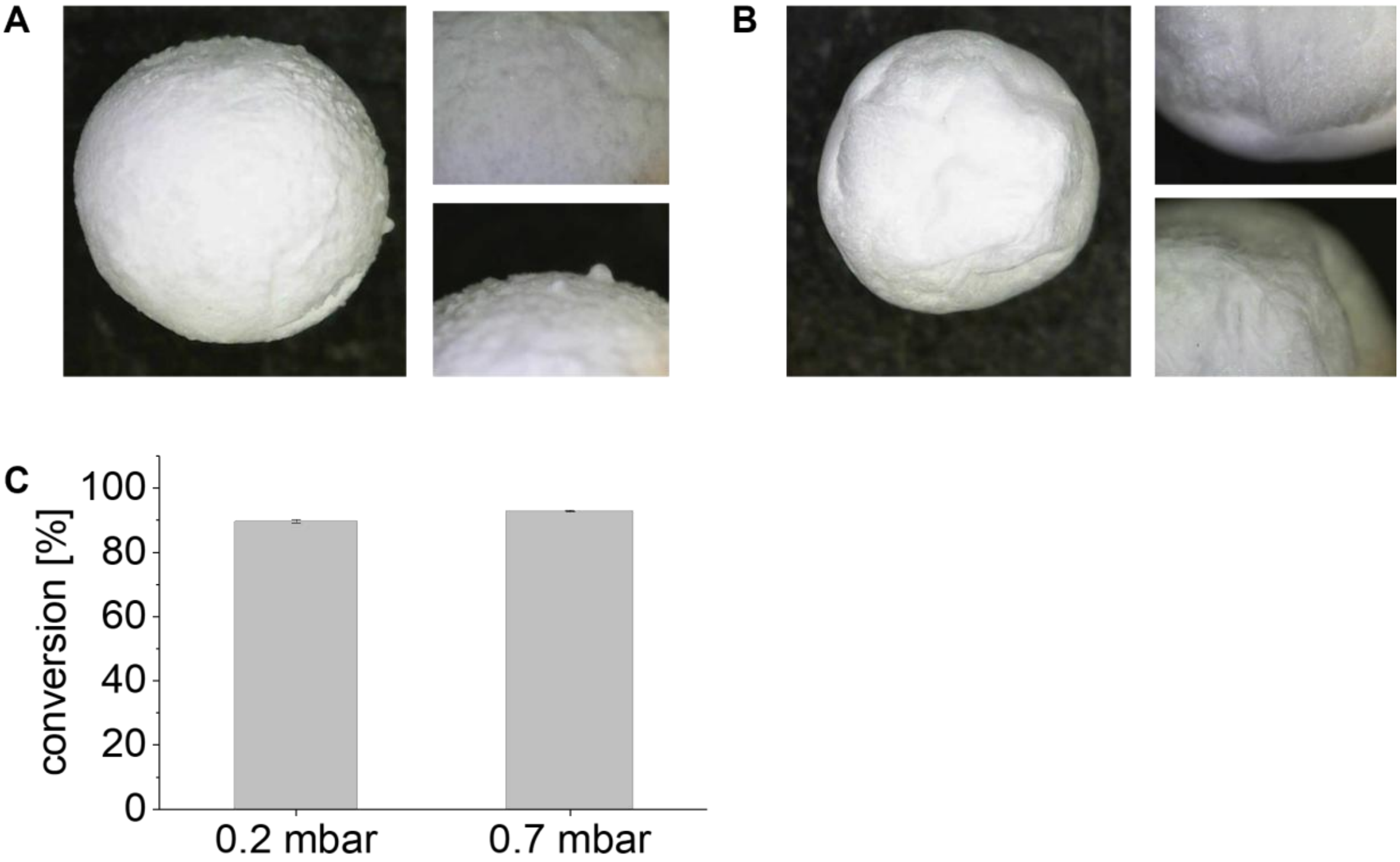

Figure S8. Influence of lyophilization pressure on AEH bead morphology and catalytic activity. Representative microscopy images of EsPAD AEH beads obtained after lyophilization at chamber pressures of (A) 0.2 mbar and (B) 0.7 mbar. Enlarged views show representative details of the bead surfaces. (C) Catalytic conversion of p-coumaric acid by EsPAD AEH beads lyophilised at 0.2 or 0.7 mbar. Despite the different thermal profiles observed during lyophilization at these pressures (Figure S6B), both conditions yielded intact AEH beads with comparable catalytic activity. Reactions were performed in 1 mL batch reactions using one AEH bead and 5 mM p-coumaric acid in H_2O. Conversion was determined by HPLC after 30 min. Data represent the mean ± SD from two independent bead preparations, each analyzed in triplicate.

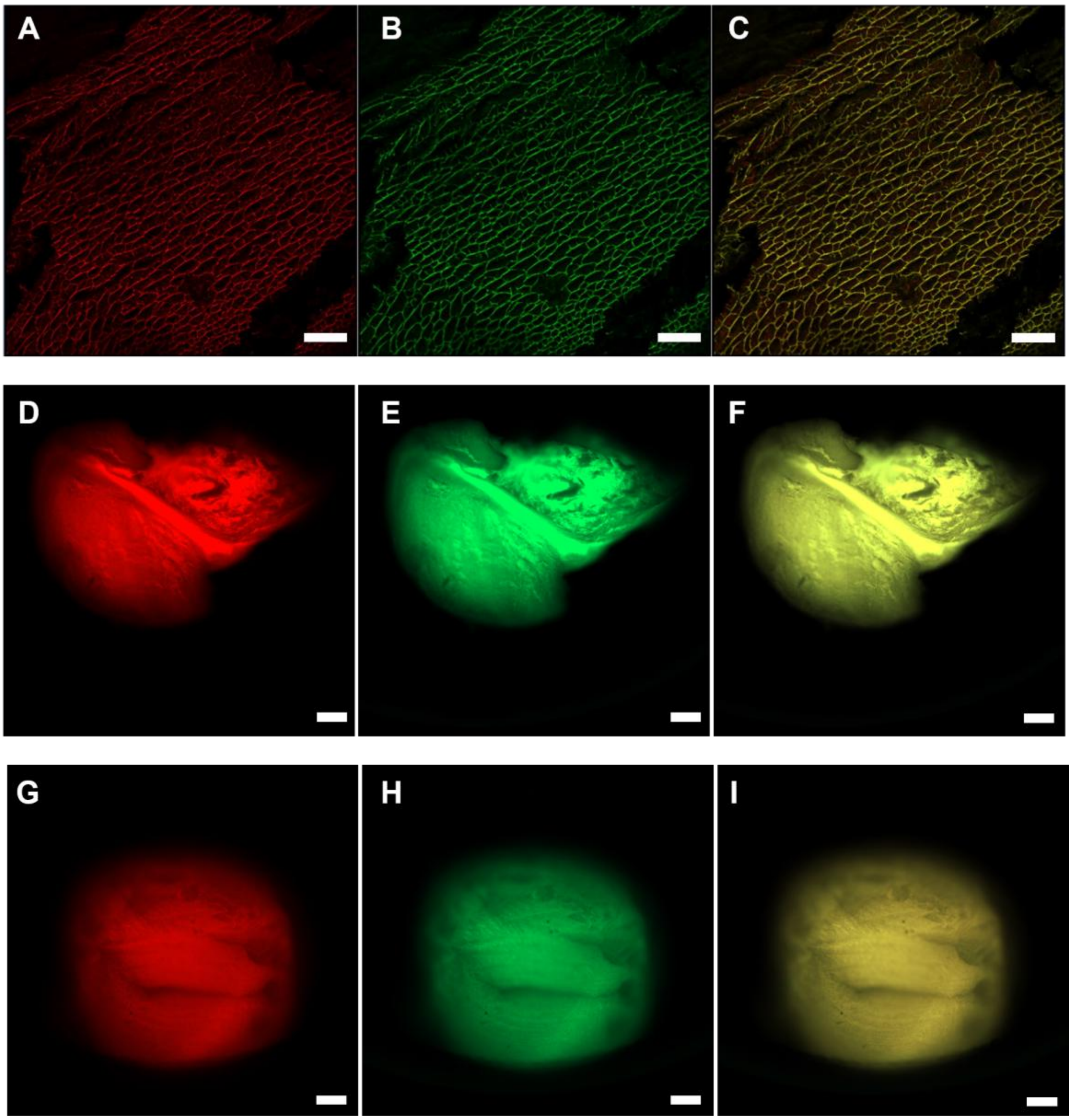

Figure S9. Fluorescence microscopy analysis of the spatial protein distribution within EsPAD AEH beads. EsPAD AEH beads were supplemented with fluorescent protein constructs at 0.1 mol% relative to a total protein concentration of 1 mM prior to bead formation. (A–F) AEH beads containing GFP-ST and RFP-ST. (A–C) Confocal laser scanning microscopy (CLSM) images showing the RFP channel (A), GFP channel (B), and corresponding merged image (C). (D–F) Wide-field fluorescence microscopy images of the same fluorescent protein combination showing the RFP channel (D), GFP channel (E), and corresponding merged image (F). (G–I) Wide-field fluorescence microscopy images of AEH beads containing GFP-SC and RFP-ST, showing the RFP channel (G), GFP channel (H), and corresponding merged image (I). The substantial spatial overlap of the red and green fluorescence signals in both fluorescent protein combinations indicates co-distribution of the respective constructs within the investigated AEH bead regions. Scale bars: A–C, 50 µm; D–I, 200 µm. Wide-field fluorescence microscopy was performed using a Zeiss Axio Observer 7.

Proposed mechanism of cryogenic AEH bead formation

AEH bead formation starts from individual enzyme building blocks equipped with complementary binding domains, exemplified here by ST-EsPAD-ST and EsPAD-SC, which undergo spontaneous covalent crosslinking upon encounter. During microfluidic bead fabrication, these proteins are combined immediately before the

resulting droplets are cryogenically fixed in liquid nitrogen. To characterize the physical conditions experienced by the assembling proteins, we first monitored the thermal history during subsequent lyophilization (Figure S6). The temperature recorded at the bottom of the flask containing the frozen EsPAD AEH beads (yellow trace, Figure S6A) showed a pronounced delay in warming compared with the empty flask (grey trace), similar to that observed for the frozen buffer control (blue trace). In particular, the water-containing samples exhibited a distinct plateau during primary drying, whereas no corresponding feature was observed for the empty flask. The plateau persisted for approximately 1.8 min for the bead preparation and 4.2 min for the larger-volume buffer control, demonstrating that removal of frozen water is gradual rather than instantaneous. Its assignment to ice sublimation is further supported by the pressure-dependent thermal profiles (Figure S6B): lowering the chamber pressure from 0.7 mbar (green trace) to 0.2 mbar (blue trace) shifted the observed sublimation regime to lower temperature, in close agreement with the calculated pressure dependence of the sublimation temperature of ice (about –23.6 and -35.7 °C for 0.7 and 0.2 mbar, respectively, using the equation of Murphy and Koop.[58]

Monitoring over the 10 h lyophilization process further showed that the measured temperature gradually approached room temperature within approximately 3 h and subsequently remained close to this level (Figure S6C). Thus, cryogenic fixation is followed by a distinct warming and drying trajectory rather than an instantaneous transition from the frozen to the dried state. This behavior is consistent with established principles of cryochemistry, according to which formation of ice excludes proteins, salts, and other solutes from the growing ice lattice and concentrates them within a progressively smaller unfrozen fraction.[29] Frozen aqueous systems are therefore heterogeneous and comprise ice-rich regions together with compositionally distinct freeze-concentrated domains whose physical state evolves during cooling and subsequent warming.[30]

The evolution of covalent protein assembly during this process was directly followed by SDS-PAGE (Figure S7). The individual ST-EsPAD-ST and EsPAD-SC building blocks are shown in lanes 1 and 2, respectively. Upon combining the complementary proteins, crosslinked species were already detectable immediately after mixing (lane 3). Importantly, crosslinked material was also clearly present directly after microfluidic mixing but before exposure to liquid nitrogen (lane 4). Molecular network formation therefore begins in solution before cryogenic fixation, consistent with the previously described solution-phase pathway of AEH formation, in which complementary SpyTag/SpyCatcher-functionalized oligomeric enzymes rapidly generate covalently crosslinked nanoscale assemblies that subsequently develop into extended protein networks.[33] Upon immersion of the droplets in liquid nitrogen, the partially assembled proteins are expected to be excluded from the growing ice phase and concentrated together within freeze-concentrated domains. This process increases their local concentration and encounter probability while simultaneously spatially confining the growing protein network. The crosslinked species remain evident directly after recovery from liquid nitrogen (lane 5), demonstrating that the covalent assemblies formed before freezing persist through cryogenic fixation. Importantly, covalently crosslinked material remains clearly detectable throughout the subsequent lyophilization process, from the sublimation plateau (lane 6) through 0 °C (lane 7), 10 °C (lane 8), and 20 °C (lane 9), with extensively crosslinked material retained after completion of lyophilization (lane 10; Figure S7).

The persistence of crosslinked material during lyophilization is particularly informative when considered together with the thermal profiles in Figure S6. The samples pass through a gradual warming trajectory during drying, such that the protein-rich phase is expected to remain at least partially hydrated during intermediate stages of the process. Molecular mobility in frozen aqueous systems is not determined solely by the nominal bulk temperature: translational motion becomes progressively restricted upon cooling, whereas water-network rearrangements and shorter-range molecular dynamics can persist over a broader temperature range.[31] Freeze-induced concentration can therefore establish close spatial proximity between complementary protein components, while increasing temperature in the presence of residual water may provide greater molecular mobility for local rearrangement and productive SC/ST encounters.

While the relative intensities of the residual uncrosslinked protein bands vary slightly between individual time points, the persistence of the high-molecular-weight fraction throughout lanes 6–9 is consistent with maintenance and potential further consolidation of the covalent network during this warming and drying phase. At the same time, freezing and ice–solution interfaces can perturb protein structure and induce conformational changes,[32] indicating that productive network formation may compete with freeze-induced structural stress. Nevertheless, changing the lyophilization pressure from 0.2 to 0.7 mbar produced intact EsPAD AEH beads with similar macroscopic morphology (Figure S8A,B) and comparable catalytic conversion of approximately 90% under the tested conditions (Figure S8C). Thus, functional bead formation is robust toward substantial changes in the thermal trajectory within the investigated processing window.

The final bead ultrastructure provides a morphological correlate of this process. ESEM analysis of bead cross-sections reveals the heterogeneous interconnected macroporous architecture, including large cavities, smaller connecting channels, corrugated internal surfaces, and locally angular or polygonal motifs (Figure 3E–H). These structures are consistent with spatial segregation into protein-rich and ice-rich domains during freezing, followed by sublimation of the latter while the increasingly crosslinked protein phase is retained. Accordingly, the macroporous structure is unlikely to result from SC/ST assembly alone, but instead reflects the combination of molecular protein assembly with freeze concentration, spatial confinement, and ice templating.

Fluorescence microscopy provides complementary evidence on the distribution of protein components within this architecture (Figure S9). Bead sections containing 0.1% GFP-ST and RFP-ST show broadly overlapping green and red fluorescence signals at both higher magnification (Figure S9A–C) and across larger bead regions (Figure S9D–F). Likewise, beads containing the complementary GFP-SC and RFP-ST constructs display overlapping distributions of both fluorescent components (Figure S9G–I). These experiments do not resolve individual SC/ST contacts at the molecular scale but demonstrate broad spatial co-distribution of protein components within the investigated regions of the bead matrix.

Taken together, the thermal profiles (Figure S6), stage-resolved crosslinking analysis (Figure S7), robustness toward altered drying conditions (Figure S8), and microscopic characterization (Figure 3E–H and Figure S9) support a multistage mechanism in which complementary protein building blocks initiate covalent assembly before freezing, become enriched and spatially confined upon ice formation, remain covalently interconnected with the potential for further network consolidation as the partially hydrated material warms during lyophilization, and are

ultimately consolidated by removal of the ice-rich phase into the characteristic macroporous AEH bead architecture.

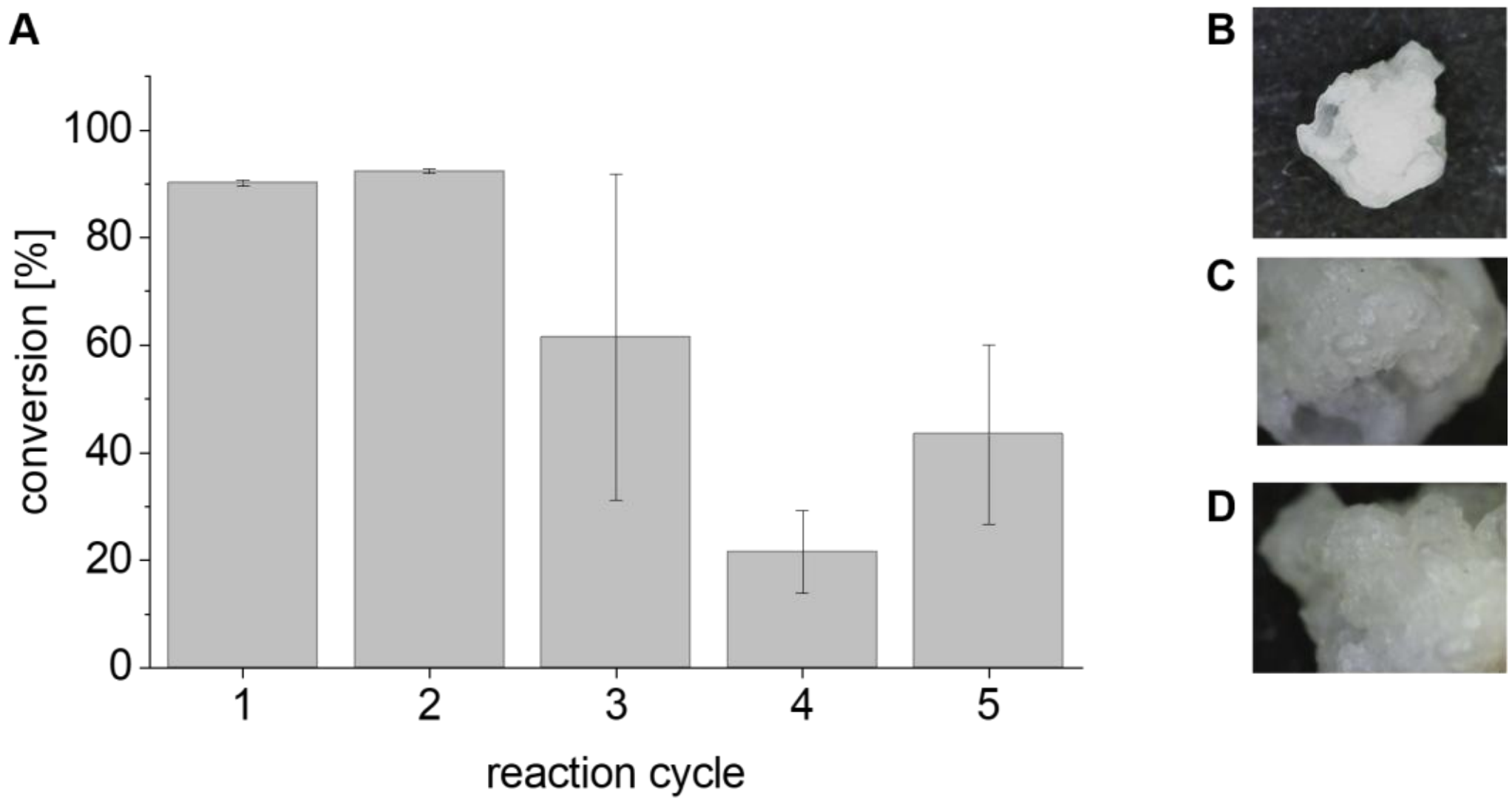

Figure S10. Reusability and morphological changes of EsPAD AEH beads during repeated batch catalysis. A) Conversion of p-coumaric acid by individual EsPAD AEH beads over five consecutive batch reaction cycles. Each cycle was performed for 30 min using one bead in a 1 mL reaction containing 5 mM p-coumaric acid in H_2O. After each cycle, the reaction solution was removed, fresh substrate solution was added, and a new zero-time sample was taken before starting the subsequent cycle. Near-quantitative conversion was retained during the first two cycles, followed by reduced and increasingly variable catalytic performance during subsequent reuse. Data are shown as mean ± SD for three independently cycled beads (n = 3). (B–D) Representative digital microscopy images of the same EsPAD AEH bead after completion of five reaction cycles, shown from different views. Pronounced morphological restructuring is evident compared with the initially spherical, lyophilized bead morphology.

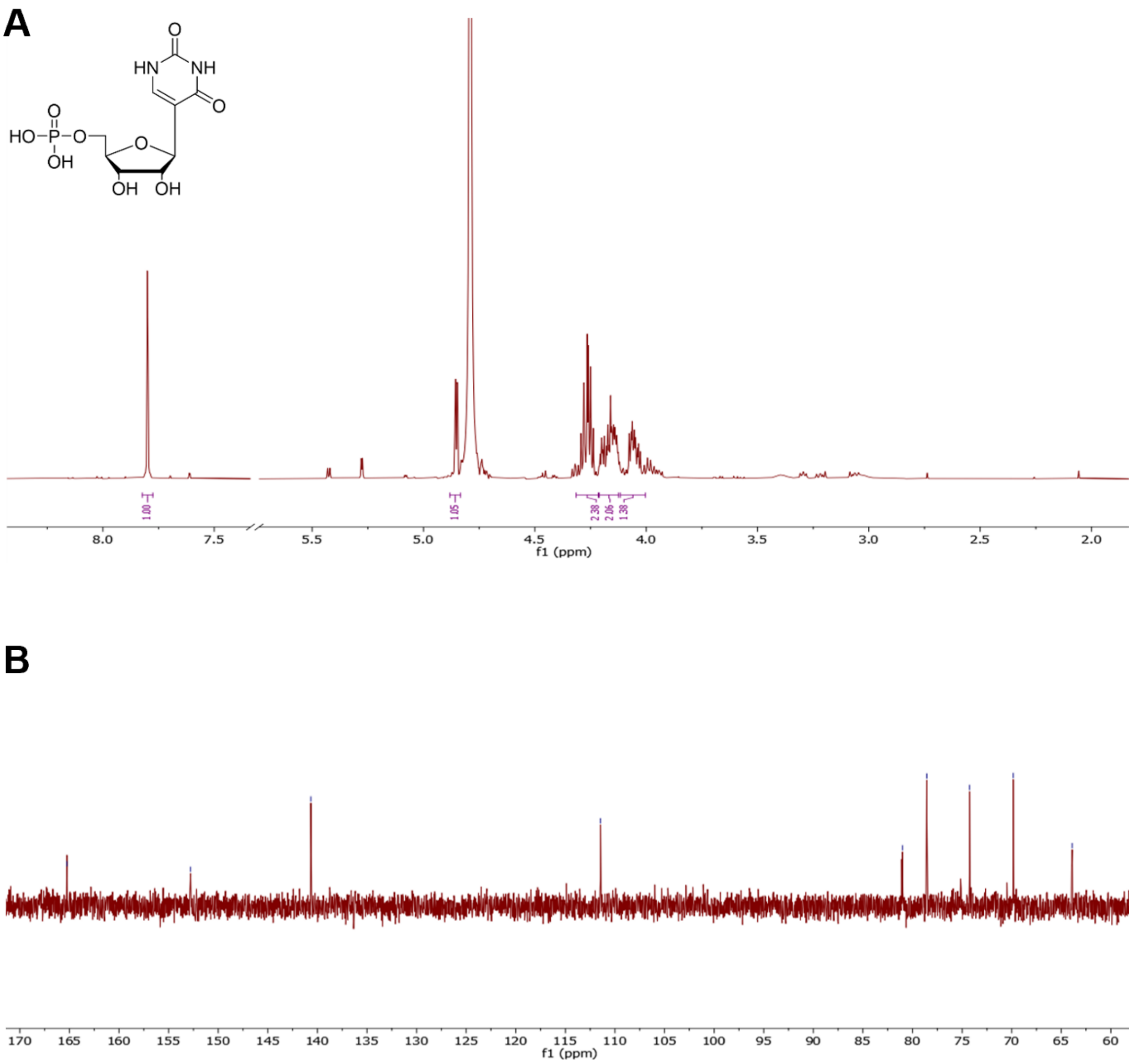

Figure S11: Nuclear magnetic resonance (NMR) spectroscopic assessment of the PMP produced from uracil and ribose-5-phosphat by EcYeiN enzyme. ^{1}H NMR (A) and ^{13}C NMR (B) spectrum of the isolated product. Inset shows the chemical structure of PMP.

^{1}H NMR (400 MHz, D_2O) δ 7.80 (d, *J* = 1.0 Hz, 1H), 4.85 (dd, *J* = 3.8, 1.0 Hz, 1H), 4.32 – 4.22 (m, 2H), 4.21 – 4.12 (m, 2H), 4.12 – 4.00 (m, 1H).

^{13}C NMR (101 MHz, D_2O) δ 165.25, 152.79, 140.66, 111.46, 81.01, 78.56, 74.24, 69.84, 63.90.

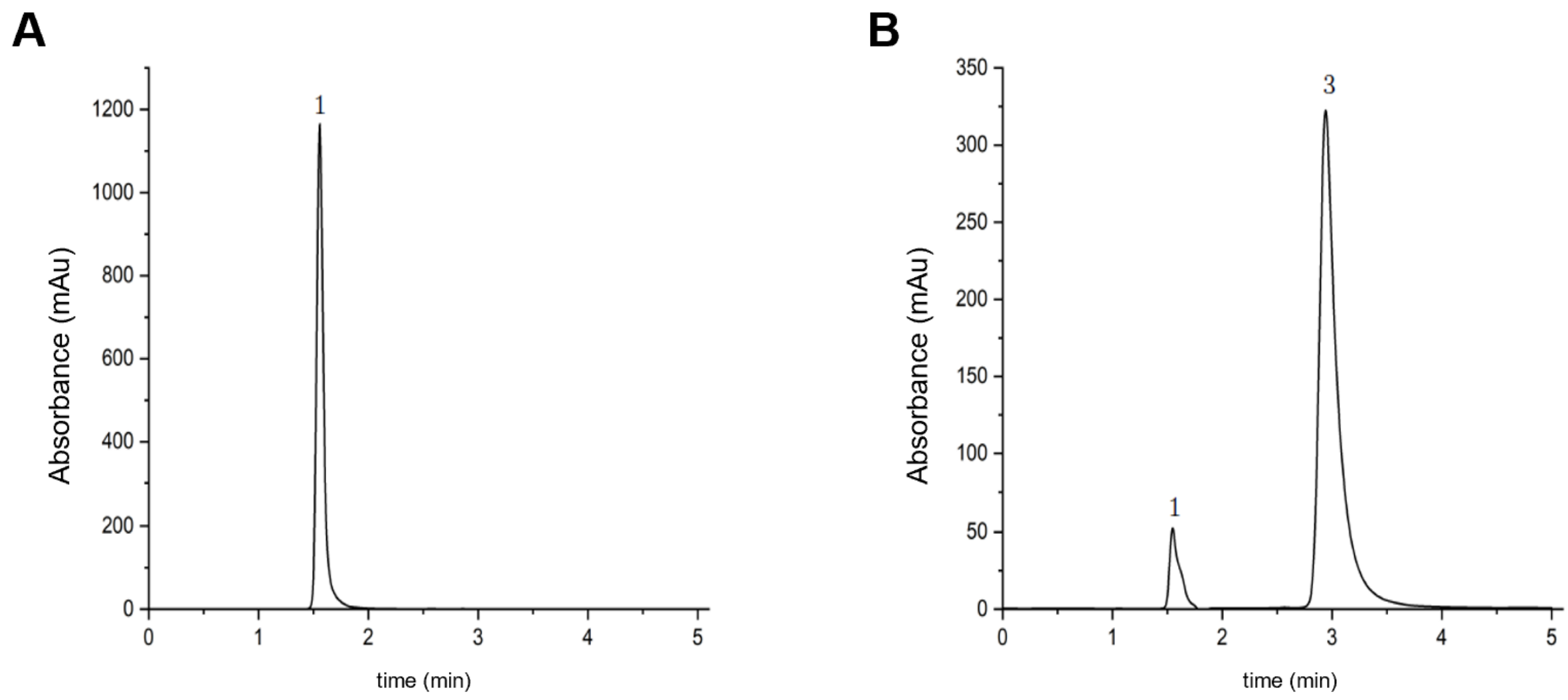

Figure S12. Representative HPLC chromatograms of uracil and reaction products formed by EcYeiN. (A) Chromatogram of pure 50 mM uracil. (B) Chromatogram of 50 mM uracil (1) partially converted to PMP (3) by 6 µM EcYeiN in reaction buffer (50 mM HEPES, 75 mM ribose-5-phosphate, 10 mM MnCl2, pH 7). Separation was performed on a C18 column (ZORBAX Eclipse XDB, 5 µm, 150 × 4.6 mm) using an isocratic mobile phase (20 mM MES buffer, 40 mM TBAB, pH 5.9) at 35 °C with a flowrate of 1 mL min^{-1}.

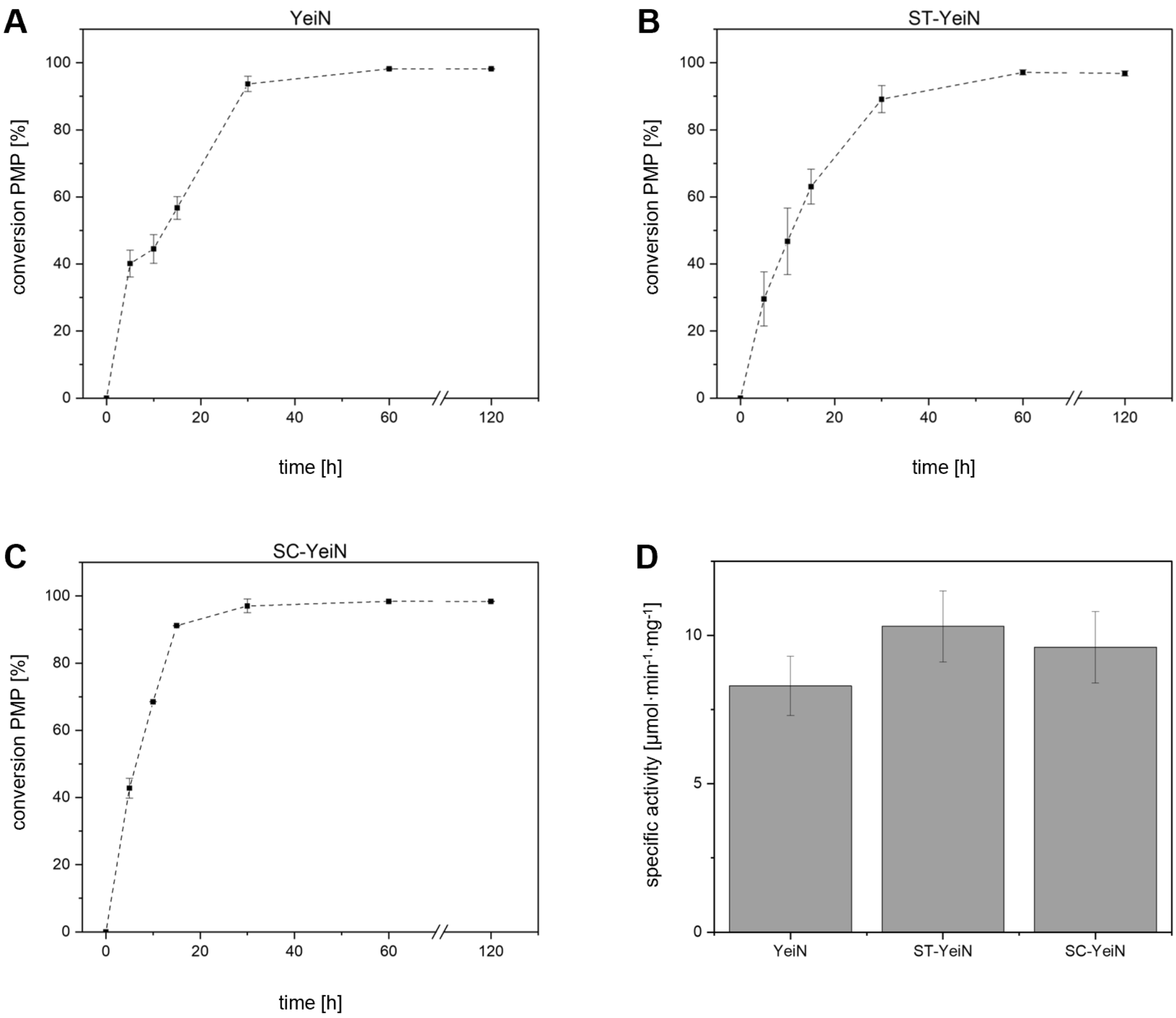

Figure S13. Activity comparison of native and SpyTag/SpyCatcher-modified EcYeiN variants. Time-dependent conversion of uracil to PMP catalyzed by His-EcYeiN (A), ST-EcYeiN (B), and SC-EcYeiN (C), quantified by RP-HPLC. (D) Comparison of specific activities calculated from the linear range of each reaction. All measurements were performed in triplicate (n = 3) and are shown as mean ± standard deviation. Reaction conditions: 6 µM enzyme, 50 mM uracil, and 75 mM ribose-5-phosphate in 50 mM HEPES buffer containing 10 mM $MnCl_2$ at pH 7, incubated at 37 °C for 2 h.

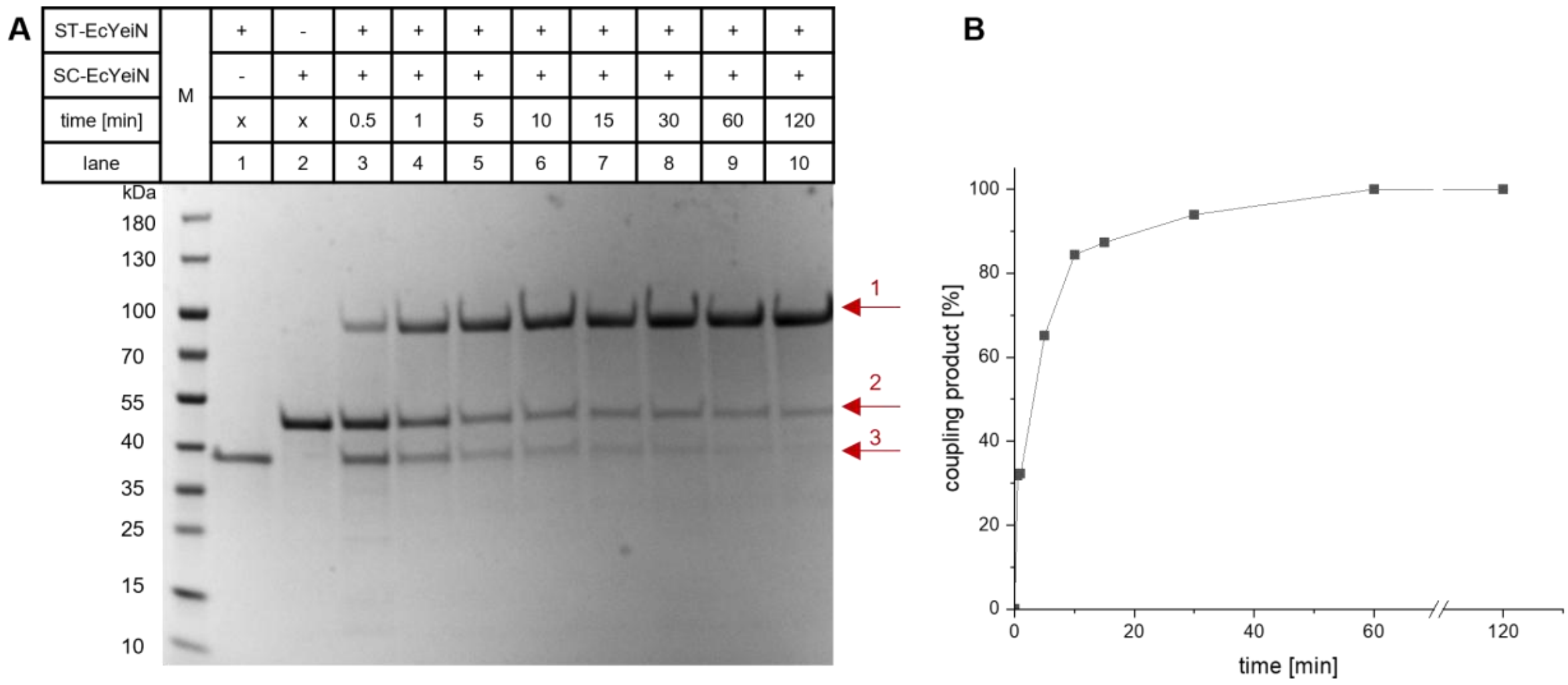

Figure S14. Verification of ST-EcYeiN and SC-EcYeiN crosslinking. (A) SDS–PAGE analysis, Coomassie stained, of the coupling reaction. Lanes 1–2 show 35 pmol of purified ST-EcYeiN and SC-EcYeiN, respectively. Reaction samples (70 pmol total protein) were collected at defined time points over 2 h. Red arrows indicate: 1 = crosslinked product, 2 = SC-EcYeiN, 3 = ST-EcYeiN. Molecular weight marker: 2 µL Prestained Protein ladder (10-180 kDa). (B) Densitometric quantification of crosslinked product formation over the 2-hour reaction period.

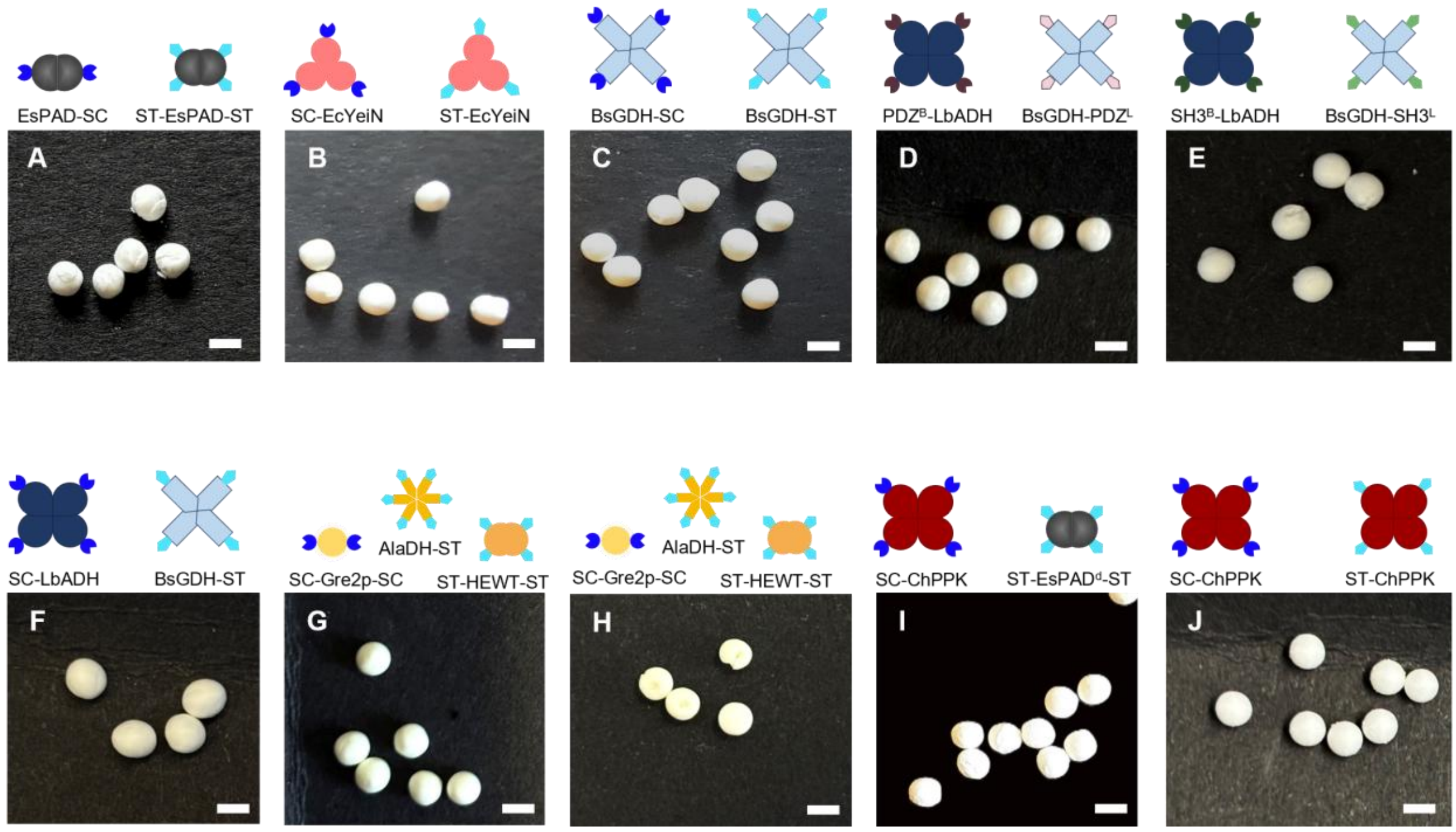

Figure S15. Representative AEH beads generated from different enzyme systems and interaction-module combinations. The protein building blocks and corresponding interaction modules used for bead formation are schematically shown above each panel; the respective recombinant enzyme constructs are characterized in Figure S1. (A) EsPAD beads assembled from EsPAD-SC and ST-EsPAD-ST. (B) EcYeiN beads assembled from SC-EcYeiN and ST-EcYeiN. (C) BsGDH beads assembled from BsGDH-SC and BsGDH-ST. (D–F) Binary LbADH/BsGDH beads assembled using PDZ (PDZ^{B}-LbADH/BsGDH-PDZ^{L}; D), SH3 (SH3^{B}-LbADH/BsGDH-SH3^{L}; E), or SpyCatcher/SpyTag (SC-LbADH/BsGDH-ST; F) interaction modules. Systems A–F were prepared at a 1:1 molar ratio of the respective interaction partners. (G,H) Ternary Gre2p/HEWT/AlaDH cascade beads assembled via SpyCatcher/SpyTag interactions from SC-Gre2p-SC, ST-HEWT-ST, and AlaDH-ST at enzyme ratios of 1:1:1 (G) or 1.5:1:1 (H); the latter composition corresponds to an overall 1:1 ratio of SpyCatcher and SpyTag interaction modules. (I) ChPPK-containing beads assembled from SC-ChPPK and the catalytically inactive EsPAD variant ST-EsPAD^{d}-ST at a 2:1 molar ratio. (J) ChPPK beads assembled from SC-ChPPK and ST-ChPPK at a 1:1 molar ratio. Scale bars, 2 mm.

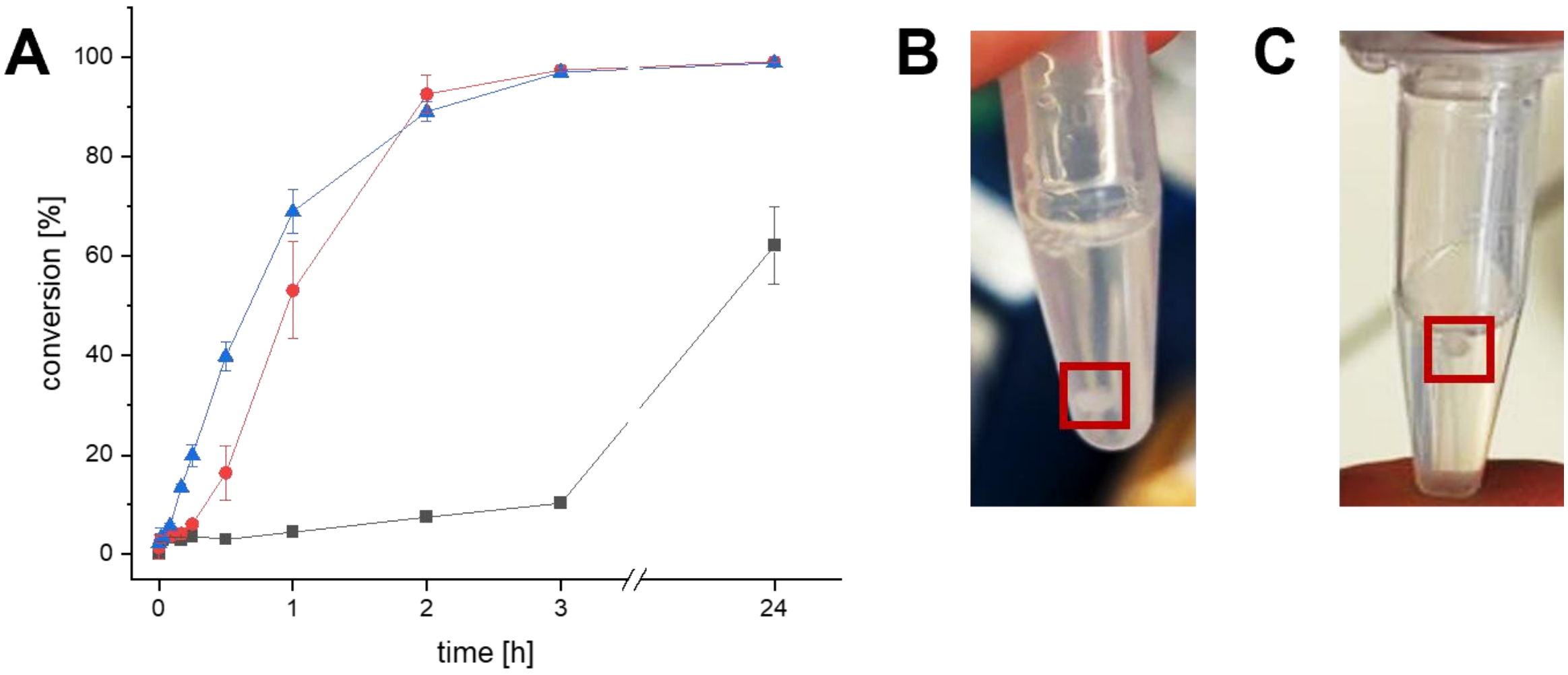

Figure S16. Stability and activity development of LbADH/BsGDH AEH beads assembled via different coupling systems under batch conditions. (A) Time-dependent NDK conversion using single LbADH/BsGDH beads assembled via PDZ (blue), SH3 (red), or SpyCatcher/SpyTag (SC/ST, black) interaction modules. The non-covalent SH3 and PDZ beads exhibit immediate catalytic activity, whereas the covalently cross-linked SC/ST bead displays a delayed onset of productivity, reaching substantial activity only after approximately 3 h. (B) Photograph of an SH3-assembled LbADH/BsGDH bead after 24 h in reaction buffer, showing pronounced swelling and transition toward a hydrogel-like morphology. (C) Photograph of an ST/SC-assembled LbADH/BsGDH bead after 24 h in reaction buffer, retaining defined spherical geometry and structural integrity. The distinct activity profiles are consistent with differences in network consolidation and swelling behavior between non-covalent and covalently cross-linked assemblies.

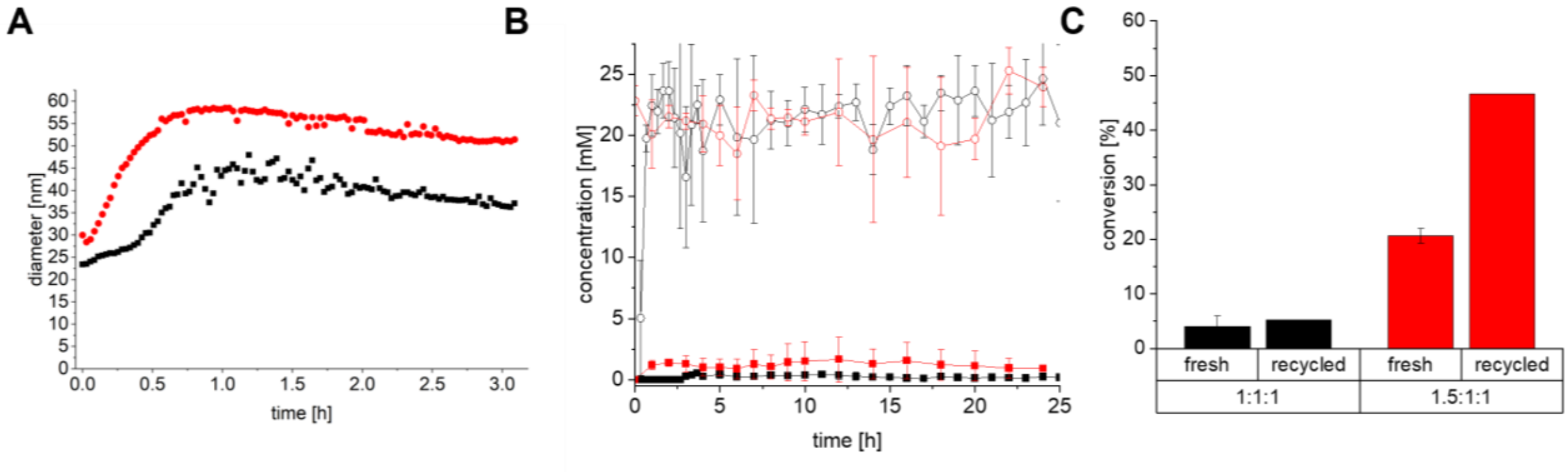

Figure S17. Influence of interaction-module stoichiometry on assembly and catalytic performance of ternary cascade AEH beads. (A) Time-resolved DLS analysis of assemblies formed from SC-Gre2p-SC, ST-HEWT-ST, and AlaDH-ST over 3 h. Proteins were combined either at an equimolar enzyme-subunit ratio of 1:1:1 (black squares) or at an adjusted ratio of 1.5:1:1 (red circles). Owing to the different numbers of interaction modules carried by the three fusion proteins, the 1:1:1 formulation corresponds to an overall SC:ST module ratio of 2:3, whereas increasing the relative amount of SC-Gre2p-SC to 1.5 yields an equimolar SC:ST module ratio of 3:3. Balancing the interaction-module stoichiometry resulted in more rapid particle growth and larger assemblies, reaching hydrodynamic diameters of approximately 55–58 nm compared with approximately 40–45 nm for the 1:1:1 formulation. (B,C) Catalytic performance of ternary AEH beads prepared at enzyme-subunit ratios of 1:1:1 (black) and 1.5:1:1 (red). (B) Concentrations of educt 1,8-octanediol (open circles) and product 8-amino-1-octanol (filled squares) during continuous-flow biocatalysis over 24 h using 25 mM 1,8-octanediol and a flow rate of 5 µL min^{-1}. (C) Substrate conversion after 24 h of batch catalysis using freshly prepared beads or beads recovered after the continuous-flow experiments shown in (B). Despite limited catalytic performance under continuous-flow conditions, the flow-recovered beads remained catalytically active in subsequent batch operation. Notably, particularly for the 1.5:1:1 formulation, the recovered beads showed higher batch conversion than freshly prepared beads. Note that this apparent increase should not necessarily be interpreted as enhanced intrinsic enzyme activity, as prolonged hydration and structural equilibration during flow operation, as well as retention or accumulation of reaction intermediates and cofactors within the bead network, may contribute to the higher subsequent batch conversion. Data in (B) are shown as mean ± SD (n = 2). For (C), fresh-bead data are shown as mean ± SD (n = 3), whereas flow-recovered data represent two independently tested beads (n = 2).

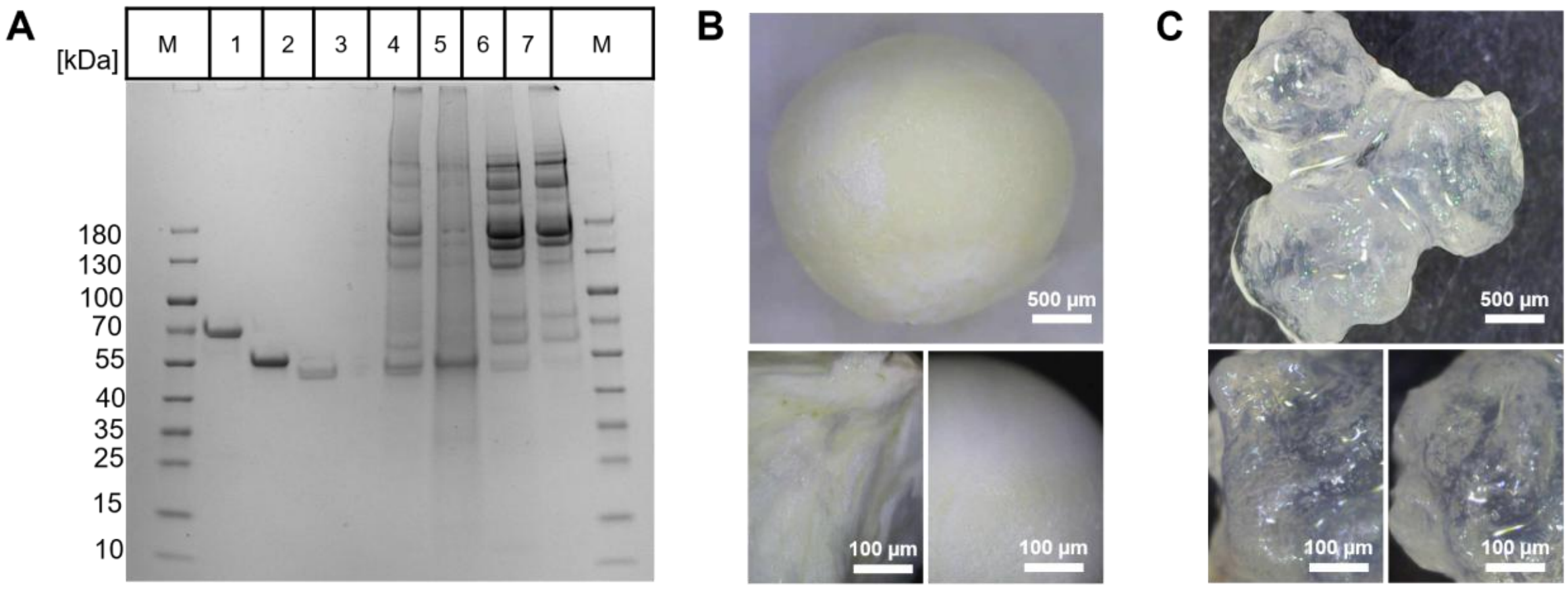

Figure S18. Structural and morphological characterization of ternary cascade AEH beads before and after catalytic application. (A) Coomassie-stained SDS-PAGE analysis of the individual, non-crosslinked cascade enzymes SC-Gre2p-SC (lane 1), ST-HEWT-ST (lane 2), and AlaDH-ST (lane 3), and of ternary AEH beads prepared at different enzyme ratios before and after catalytic application. Beads containing SC-Gre2p-SC, ST-HEWT-ST, and AlaDH-ST at an equimolar enzyme-subunit ratio of 1:1:1 were analyzed before (lane 4) and after (lane 5) application, whereas beads prepared at the interaction-module-balanced ratio of 1.5:1:1 were analyzed before (lane 6) and after (lane 7) application. The latter formulation provides an overall equimolar SC:ST interaction-module ratio, as described in Figure S17. In contrast to the individual enzyme preparations, the bead samples show pronounced high-molecular-weight material resulting from SC/ST-mediated covalent crosslinking. High-molecular-weight crosslinked material remained clearly detectable after catalytic application, demonstrating persistence of covalent protein assemblies during bead use. For analysis, bead samples were dispersed in SDS loading buffer, and an equivalent total protein amount of 2 µg was loaded per lane. M, prestained protein molecular-weight marker. (B) Representative digital microscopy images of freshly lyophilized ternary AEH beads prepared at an enzyme-subunit ratio of 1:1:1. Enlarged views show details of the bead surface. (C) Representative digital microscopy images of corresponding beads after catalytic application followed by storage in 100 mM KPi buffer (pH 7.5) for approximately two weeks. Upon hydration and catalytic application, the initially opaque, white lyophilized beads became transparent while retaining their macroscopic integrity. Prolonged storage in the hydrated state resulted in pronounced morphological restructuring compared with the freshly lyophilized material, without complete disintegration of the beads.

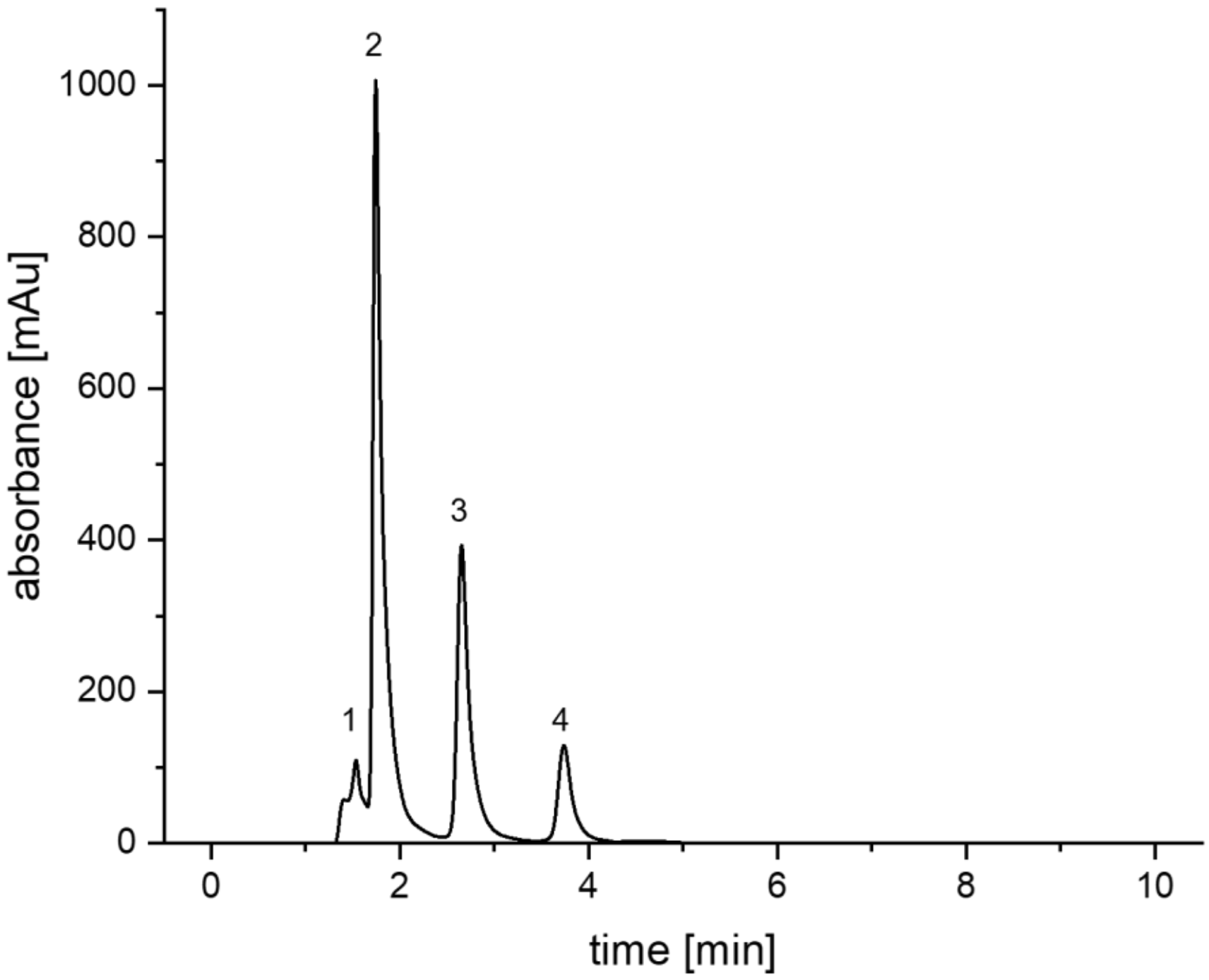

Figure S19. Representative HPLC chromatogram of PMP (2), PDP (3) and PTP (4) separation after ChPPK reaction. Separation was performed in a C18 column (ZORBAX Eclipse XDB, 5 µm, 150 × 4.6 mm) using an isocratic mobile phase (100 mM potassium phosphate, 10 mM TBAB, pH 6.4 and 7.5% acetonitrile) at 22 °C and a flow rate of 1.3 mL min-1. The analytes were detected at 260 nm with the following retention times: t_R (uracil, 1) = 1.3-1.5 min, t_R (PMP, 2) = 1.7-1.9 min, t_R (PDP, 3) = 2.6-2.9 min and t_R (PTP, 4) = 3.9-4.4 min.

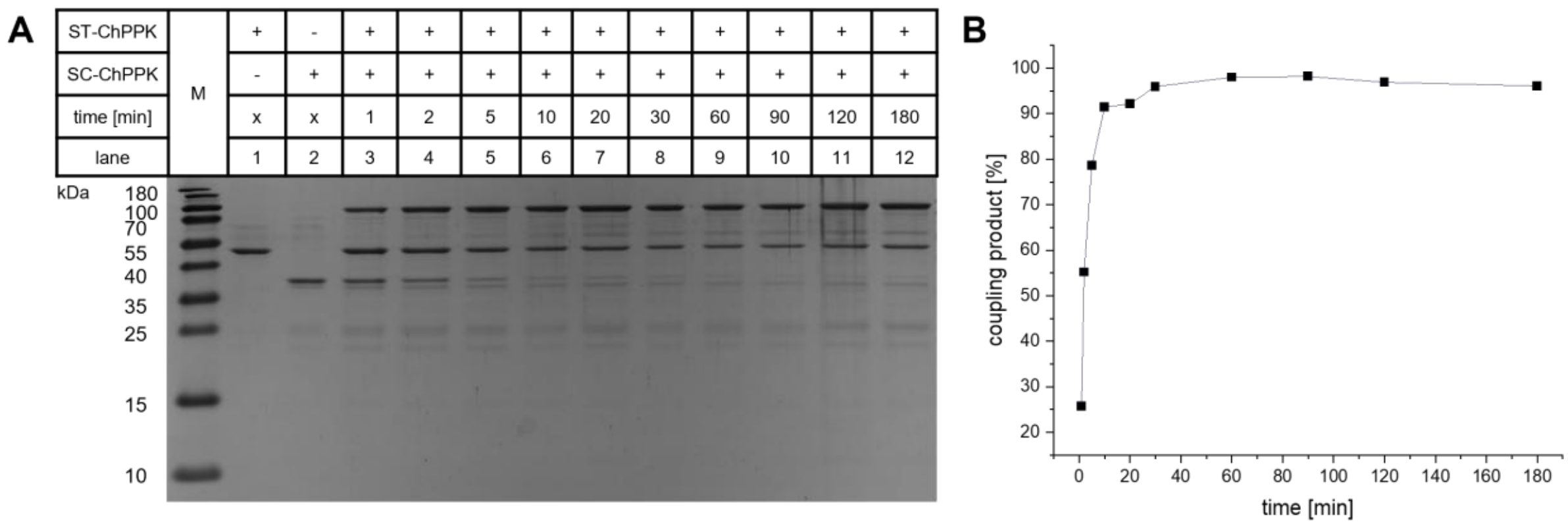

Figure S20. Verification of ST-ChPPK and SC-ChPPK crosslinking. (A) Coomassie stained SDS-PAGE analysis of the purified enzyme variants of ST-ChPPK (lane 1, 38 kDa) and SC-ChPPK (lane 2, 48 kDa) fused by the covalent interaction systems SC/ST. Verification of ST-ChPPK and SC-ChPPK coupling (lane 3 to 12). M: Prestained Protein ladder (10-180 kDa). (B) Densitometric quantification of cross-linked product formation over a 3-hour reaction period.

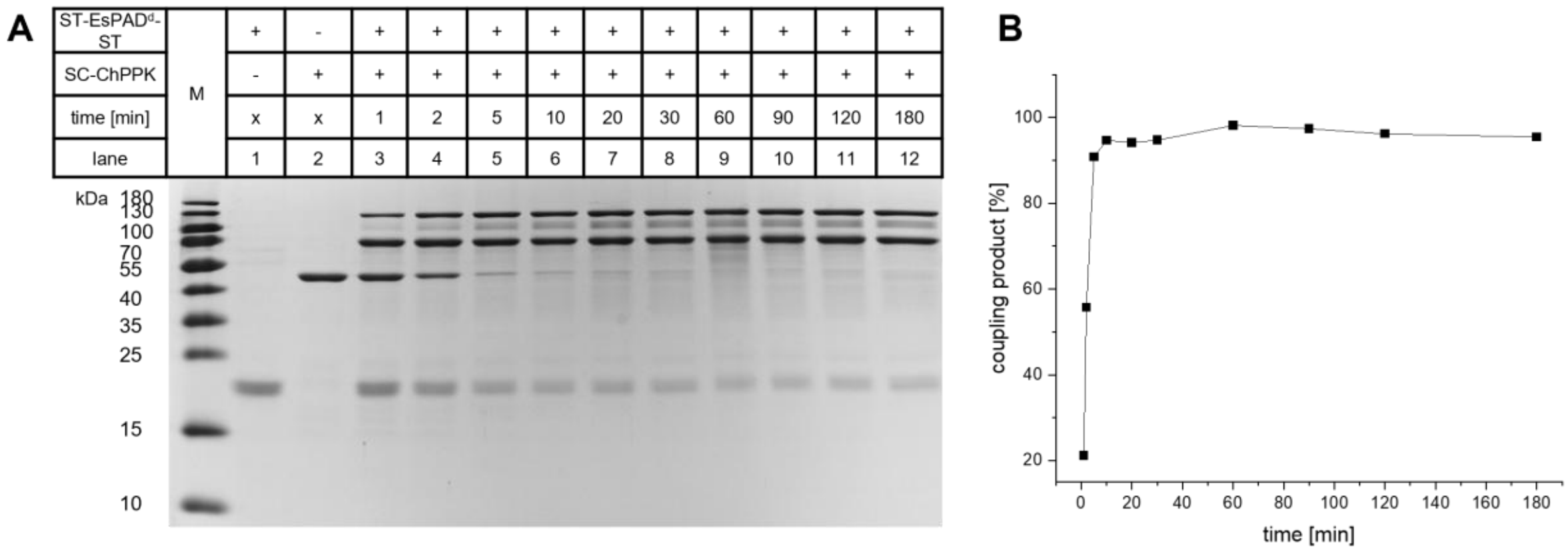

Figure S21. Verification of ST-EsPAD^d-ST and SC-ChPPK crosslinking. (A) Coomassie stained SDS-PAGE analysis of the purified enzyme variants of ST-EsPAD^d-ST (lane 1, 24 kDa) and SC-ChPPK (lane 2, 48 kDa) fused by the covalent interaction systems SC/ST. Verification of ST-EsPAD^d-ST and SC-ChPPK coupling (lane 3 to 12). M: Prestained Protein ladder (10-180 kDa). (B) Densitometric quantification of cross-linked product formation over a 3-hour reaction period.

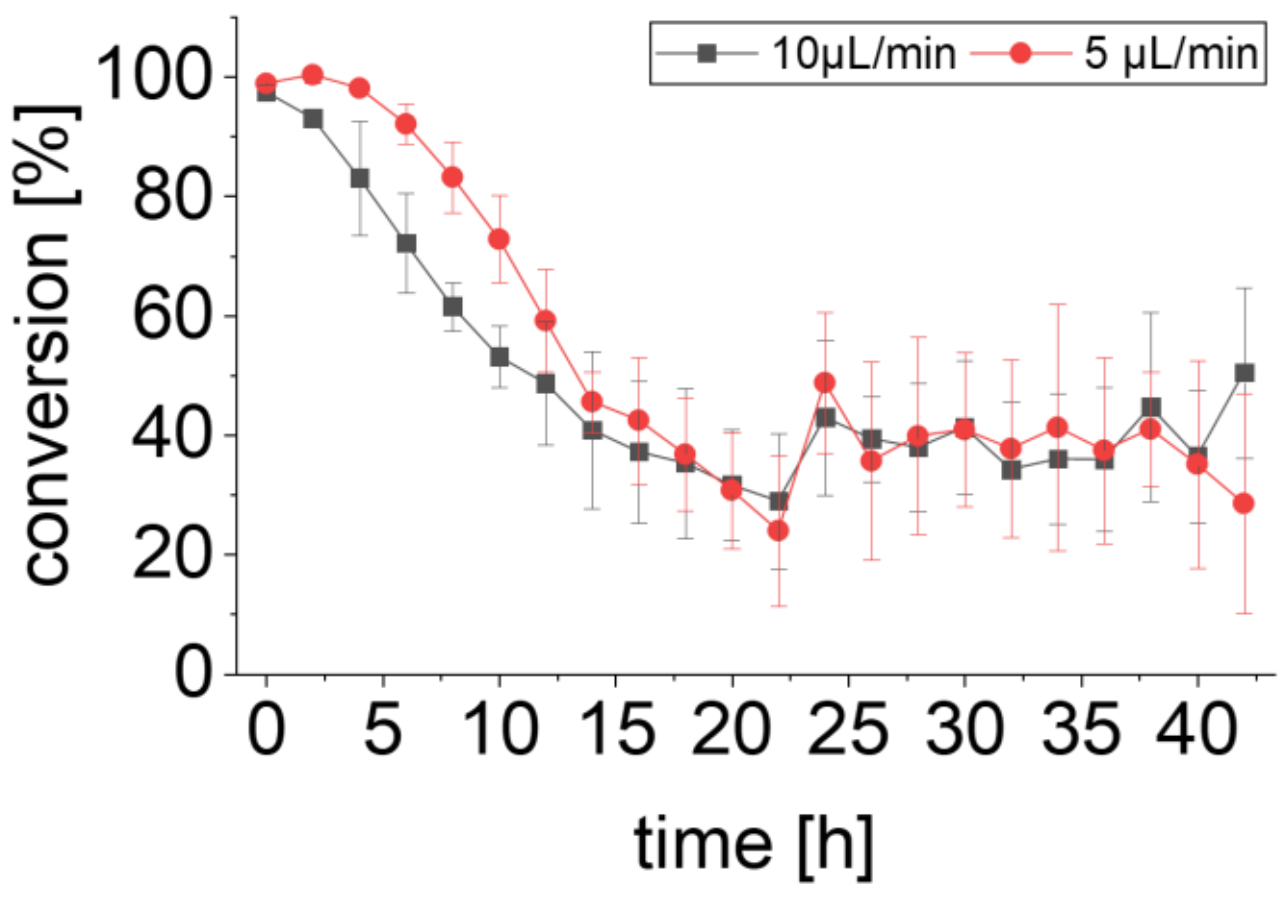

Figure S22. Long-term operational stability of EsPAD AEH beads under continuous-flow conditions. Conversion of p-coumaric acid by EsPAD AEH beads during continuous operation for 42 h at flow rates of 5 µL min^{-1} (red circles) and 10 µL min^{-1} (black squares). At both flow rates, initially near-quantitative conversion progressively decreased during the first approximately 20 h, followed by sustained conversion of approximately 30–45% for the remainder of the experiment. Measurements were performed in triplicate; data are presented as mean ± standard deviation.

Influence of flow rate and residence time on EsPAD AEH bead performance. Flow rate represents an important process parameter for continuously operated all-enzyme materials, as it determines substrate residence time and may additionally influence the operational stability of the material under flow conditions.[24, 25] For the 100 µL AEH bead reactor used here, the nominal liquid residence time was estimated based on the reactor volume, four beads per reactor, and an average bead diameter of approximately 2.0 mm (Figure S4). Depending on whether the highly porous intrabead volume is considered accessible to the flowing liquid phase, estimated residence times are approximately 17–19 min at 5 µL min^{-1} and 8–10 min at 10 µL min^{-1}**.** These values should be regarded as estimates rather than experimentally determined residence-time distributions. Consistent with the longer substrate–catalyst contact time, operation at 5 µL min^{-1} resulted in higher conversion than at 10 µL min^{-1} (Figure S22). At both flow rates, conversion initially decreased before reaching sustained residual levels during prolonged operation, indicating that instantaneous reactor performance and long-term operational stability represent related but distinct aspects of AEH bead performance.

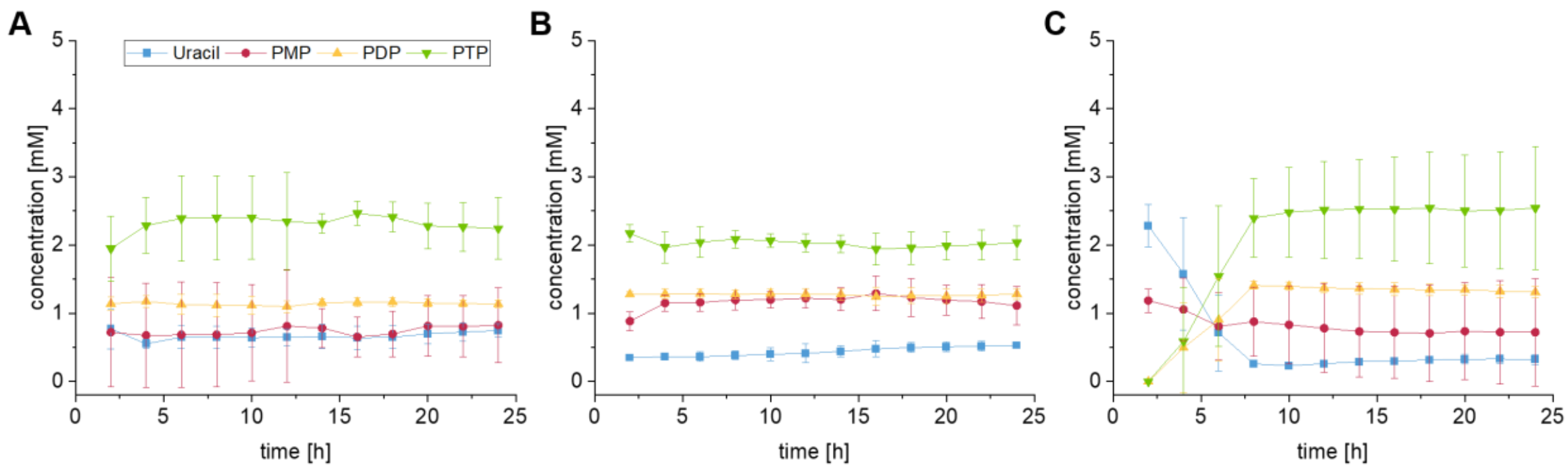

Figure S23. Operational performance of different ChPPK-containing AEH bead configurations under continuous-flow conditions. Concentration profiles of uracil (blue squares), PMP (red circles), PDP (orange triangles), and PTP (green inverted triangles) during 24 h of continuous-flow operation using different combinations of EcYeiN and ChPPK-containing AEH beads. (A) EcYeiN AEH beads combined with SC-ChPPK/ST-EsPADd-ST beads; the reactor contained one EcYeiN bead and three SC-ChPPK/ST-EsPADd-ST beads. (B) Free EcYeiN combined with four SC-ChPPK/ST-EsPADd-ST beads. (C) Free EcYeiN combined with four SC-ChPPK/ST-ChPPK beads. The substrate solution contained 5 mM uracil, 60 mM sodium hexametaphosphate (SHMP), 40 mM $MgCl_2$, and 7.5 mM ribose-5-phosphate in 50 mM HEPES buffer (pH 7). For configurations (B) and (C), 6 µM EcYeiN was additionally supplied in the substrate solution. PDP and PTP were detected throughout the investigated 24 h period for all three reactor configurations. Note that differences in the early concentration profiles may additionally reflect reactor equilibration and the timing of initial sampling and were therefore not used to compare start-up kinetics between the individual configurations. Data are shown as mean ± SD [n = 3]

Supplementary tables

Table S1: Codon optimized genes of proteins used in this study.

EsPAD-SC-His (Mittmann et al. 2019)[22]

ATGAACACCTTCGACAAACATGATCTGAGCGGTTTTGTTGGTAAACATCTGGTGTATAC
CTATGATAATGGCTGGGAGTATGAGATCTATGTGAAAAATGAAAACACCCTGGATTATC
GCATTCATAGCGGTCTGGTTGGTAATCGTTGGGTTAAAGATCAGCAGGCATATATTGT
TCGTGTGGGTGAAAGCATCTATAAAATCAGCTGGACCGAACCGACCGGCACCGATGT
TAGCCTGATTGTTAATCTGGGTGATAGCCTGTTTCATGGCACCATCTTTTTTCCGCGTT
GGGTGATGAATAATCCGGAAAAAACCGTTTGCTTTCAGAACGATCATATTCCGCTGAT
GAATAGCTATCGTGATGCAGGTCCGGCATATCCGACCGAAGTTATTGATGAATTTGCC
ACCATTACCTTTGTTCGTGATTGTGGTGCAAATAACGAAAGCGTTATTGCATGTGCAGC
AAGCGAACTGCCGAAAAACTTTCCGGATAATCTGAAAGGTGGTGGTGGTAGCGTTGAT
ACCCTGAGCGGTCTGAGCAGCGAACAGGGTCAGAGCGGTGATATGACCATTGAAGAA
GATAGCGCAACCCACATCAAATTCAGCAAACGTGATGAAGATGGTAAAGAACTGGCAG
GCGCAACCATGGAACTGCGTGATAGCAGCGGTAAAACCATTAGCACCTGGATTAGTG
ATGGTCAGGTGAAAGATTTTTATCTGTACCCTGGCAAATACACCTTTGTTGAAACCGCA
GCACCGGATGGTTATGAAGTTGCAACCGCAATTACCTTTACCGTTAATGAACAGGGCC
AGGTTACCGTGAATGGTAAAGCAACCAAAGGTGATGCACATATTGGTCATCATCACCA
TCATCATTAA

ST-EsPAD-ST-His (Mittmann et al. 2019)[22]

ATGGCTCACATCGTTATGGTGGACGCTTACAAACCGACCAAAGGTGGTGGTGGTAGC
AACACCTTCGACAAACATGATCTGAGCGGTTTTGTTGGTAAACATCTGGTGTATACCTA
TGATAATGGCTGGGAGTATGAGATCTATGTGAAAAATGAAAACACCCTGGATTATCGC
ATTCATAGCGGTCTGGTTGGTAATCGTTGGGTTAAAGATCAGCAGGCATATATTGTTC
GTGTGGGTGAAAGCATCTATAAAATCAGCTGGACCGAACCGACCGGCACCGATGTTA
GCCTGATTGTTAATCTGGGTGATAGCCTGTTTCATGGCACCATCTTTTTTCCGCGTTGG
GTGATGAATAATCCGGAAAAAACCGTTTGCTTTCAGAACGATCATATTCCGCTGATGAA
TAGCTATCGTGATGCAGGTCCGGCATATCCGACCGAAGTTATTGATGAATTTGCCACC
ATTACCTTTGTTCGTGATTGTGGTGCAAATAACGAAAGCGTTATTGCATGTGCAGCAAG
CGAACTGCCGAAAAACTTTCCGGATAATCTGAAAGGTGGTGGTGGTAGCGCACATATT
GTTATGGTTGATGCATATAAACCGACCAAAGGTCATCATCACCATCATCATTAA

His-EcYeiN (this work)

ATGCATCATCACCATCATCATGGTGGTGGCGGTTCAAGCGAACTGAAAATTAGCCCGG
AACTGCTGCAGATTAGCCCGGAAGTGCAGGATGCGCTGAAAAACAAAAAACCGGTGG
TGGCGCTGGAAAGCACCATTATTAGCCATGGCATGCCGTTTCCGCAGAACGCGCAGA
CCGCGATTGAAGTGGAAGAAACCATTCGCAAACAGGGCGCGGTGCCGGCGACCATT
GCGATTATTGGCGGCGTGATGAAAGTGGGCCTGAGCAAAGAAGAAATTGAACTGCTG
GGCCGCGAAGGCCATAACGTGACCAAAGTGAGCCGCCGCGATCTGCCGTTTGTGGT
GGCGGCGGGCAAAAACGGCGCGACCACCGTGGCGAGCACCATGATTATTGCGGCGC
TGGCGGGCATTAAAGTGTTTGCGACCGGCGGCATTGGCGGCGTGCATCGCGGCGCG
GAACATACCTTTGATATTAGCGCGGATCTGCAGGAACTGGCGAACACCAACGTGACC
GTGGTGTGCGCGGGCGCGAAAAGCATTCTGGATCTGGGCCTGACCACCGAATATCTG
GAAACCTTTGGCGTGCCGCTGATTGGCTATCAGACCAAAGCGCTGCCGGCGTTTTTTT
GCCGCACCAGCCCGTTTGATGTGAGCATTCGCCTGGATAGCGCGAGCGAAATTGCGC
GCGCGATGGTGGTGAAATGGCAGAGCGGCCTGAACGGCGGCCTGGTGGTGGCGAAC

CCGATTCCGGAACAGTTTGCGATGCCGGAACATACCATTAACGCGGCGATTGATCAG
GCGGTGGCGGAAGCGGAAGCGCAGGGCGTGATTGGCAAAGAAAGCACCCCGTTTCT
GCTGGCGCGCGTGGCGGAACTGACCGGCGGCGATAGCCTGAAAAGCAACATTCAGC
TGGTGTTTAACAACGCGATTCTGGCGAGCGAAATTGCGAAAGAATATCAGCGCCTGG
CGGGCTAATGA

His-ST-EcYeiN (this work)

ATGCATCACCACCATCATCATGGTGCACACATAGTAATGGTAGACGCCTACAAGCCGAC
GAAGGGCGGTGGTGGTAGCAGCGAACTGAAAATTAGTCCGGAACTGCTGCAGATTTCA
CCGGAAGTTCAGGATGCACTGAAAAATAAGAAACCGGTTGTTGCACTGGAAAGCACCAT
TATTAGCCATGGTATGCCGTTTCCGCAGAATGCACAGACCGCAATTGAAGTTGAAGAAA
CCATTCGTAAACAGGGTGCCGTTCCGGCAACCATTGCAATTATTGGTGGTGTTATGAAA
GTGGGCCTGAGCAAAGAAGAAATTGAACTGCTGGGTCGTGAAGGTCATAATGTTACCAA
AGTTAGCCGTCGTGATCTGCCGTTTGTTGTTGCAGCAGGTAAAAATGGTGCAACCACCG
TTGCAAGTACCATGATTATTGCAGCACTGGCAGGTATTAAAGTTTTTGCGACCGGTGGT
ATTGGTGGCGTTCATCGTGGTGCAGAACATACCTTTGATATTAGTGCCGATCTGCAAGA
ACTGGCCAATACCAATGTTACCGTTGTTTGTGCCGGTGCAAAAAGCATTCTGGATCTGG
GTCTGACCACCGAATATCTGGAAACCTTTGGTGTTCCGCTGATTGGTTATCAGACCAAA
GCACTGCCTGCATTTTTCTGTCGTACCAGTCCGTTTGATGTTAGCATTCGTCTGGATAGC
GCAAGCGAAATTGCACGTGCCATGGTTGTTAAATGGCAGAGCGGTCTGAATGGTGGTC
TGGTTGTTGCCAATCCGATTCCGGAACAGTTTGCAATGCCGGAACATACCATTAATGCA
GCAATTGATCAGGCAGTTGCCGAAGCCGAAGCACAGGGTGTTATTGGTAAAGAAAGCA
CCCCGTTTCTGCTGGCACGTGTTGCAGAACTGACCGGTGGCGATAGCCTGAAAAGCAA
TATTCAGCTGGTTTTTAACAATGCCATTCTGGCATCAGAAATCGCCAAAGAATATCAGCG
TCTGGCAGGTTAATGA

His-SC-EcYeiN (this work)

ATGCATCACCACCATCATCATGGTGTGGATACCCTGAGCGGTCTGAGCAGCGAACAGG
GTCAGAGCGGTGATATGACCATTGAAGAAGATAGCGCAACCCACATCAAATTCAGCAAA
CGTGATGAAGATGGCAAAGAACTGGCAGGCGCAACCATGGAACTGCGTGATAGCAGCG
GTAAAACCATTAGCACCTGGATTAGTGATGGTCAGGTGAAAGATTTTTATCTGTATCCGG
GTAAATATACCTTCGTTGAAACCGCAGCACCGGATGGTTATGAAGTTGCAACCGCAATT
ACCTTTACCGTGAATGAACAAGGTCAGGTTACCGTTAATGGTAAAGCAACCAAAGGTGA
TGCACATATTGGCGGTGGTGGTAGCAGCGAACTGAAAATTAGTCCGGAACTGCTGCAG
ATTTCACCGGAAGTTCAGGATGCACTGAAAAATAAGAAACCGGTTGTTGCACTGGAAAG
CACCATTATTAGCCATGGTATGCCGTTTCCGCAGAATGCACAGACCGCAATTGAAGTTG
AAGAAACCATTCGTAAACAGGGTGCCGTTCCGGCAACCATTGCAATTATTGGTGGTGTT
ATGAAAGTGGGCCTGAGCAAAGAAGAAATTGAACTGCTGGGTCGTGAAGGTCATAATGT
TACCAAAGTTAGCCGTCGTGATCTGCCGTTTGTTGTTGCAGCAGGTAAAAATGGTGCAA
CCACCGTTGCAAGTACCATGATTATTGCAGCACTGGCAGGTATTAAAGTTTTTGCGACC
GGTGGTATTGGTGGCGTTCATCGTGGTGCAGAACATACCTTTGATATTAGTGCCGATCT
GCAAGAACTGGCCAATACCAATGTTACCGTTGTTTGTGCCGGTGCAAAAAGCATTCTGG
ATCTGGGTCTGACCACCGAATATCTGGAAACCTTTGGTGTTCCGCTGATTGGTTATCAG
ACCAAAGCACTGCCTGCATTTTTCTGTCGTACCAGTCCGTTTGATGTTAGCATTCGTCTG
GATAGCGCAAGCGAAATTGCACGTGCCATGGTTGTTAAATGGCAGAGCGGTCTGAATG
GTGGTCTGGTTGTTGCCAATCCGATTCCGGAACAGTTTGCAATGCCGGAACATACCATT
AATGCAGCAATTGATCAGGCAGTTGCCGAAGCCGAAGCACAGGGTGTTATTGGTAAAGA
AAGCACCCCGTTTCTGCTGGCACGTGTTGCAGAACTGACCGGTGGCGATAGCCTGAAA
AGCAATATTCAGCTGGTTTTTAACAATGCCATTCTGGCATCAGAAATCGCCAAAGAATAT
CAGCGTCTGGCAGGTTAATGA

His-ST-HEWT-ST (this work)

ATGCATCACCACCATCATCATGGTGCCCATATTGTTATGGTTGATGCCTATAAACCGACC
AAAGGTGGTGGTGGTAGTCAGACCCAGGATTATCAGGCACTGGATCGTGCACATCATCT
GCATCCGTTTACCGATTTTAAAGCACTGGGTGAAGAAGGTAGTCGCGTTGTTACCCATG
CCGAAGGTGTTTATATCCATGATAGCGAAGGTAATCGTATTCTGGATGGTATGGCAGGT
CTGTGGTGTGTTAATTTAGGTTATGGTCGTCGTGAACTGGTTGAAGCAGCAACCGCACA
GCTGGAACAGCTGCCGTATTATAACACCTTTTTCAAAACCACACATCCGCCTGCAGTTC
GTCTGGCAGAAAAACTGTGTGATCTGGCACCGGCACATATTAATCGTGTGTTTTTTACC
GGTAGCGGTAGCGAAGCAAATGATACCGTTCTGCGTATGGTTCGTCGTTATTGGGCACT
GAAAGGTCAGCCGGATAAACAGTGGATTATTGGTCGTGAAAATGCCTATCATGGTAGCA
CCCTGGCAGGTATGAGCTTAGGTGGCATGGCACCGATGCATGCACAAGGTGGTCCGTG
TGTTCCGGGTATTGCACATATTCGTCAGCCGTATTGGTTTGGTGAAGGTCGTGATATGA
GTCCGGAAGCATTTGGTCAGACCTGTGCAGAAGCACTGGAAGAAAAAATTCTGGAACTG
GGCGAAGAAAAAGTGGCAGCATTTATTGCAGAACCGGTGCAAGGTGCCGGTGGTGCAA
TTATGCCTCCGGAAAGCTATTGGCCTGCCGTTAAAAAAGTTCTGGCCAAATATGATATTC
TGCTGGTTGCCGATGAAGTGATTTGCGGTTTTGGTCGTTTAGGTGAATGGTTTGGTAGC
CAGCATTATGGTCTGGAACCGGATCTGATGCCGATTGCAAAAGGTCTGAGCAGCGGTTA
TCTGCCGATTGGTGGTGTTCTGGTTGGTGATCGTGTTGCAGAAACCCTGATCGAAGAAG
GTGGCGAATTTTTCCATGGTTTTACCTATAGCGGTCATCCGACCTGTGCCGCAGTTGCA
CTGAAAAACCTGGAACTGCTGGAAGCGGAAGGTGTTGTGGATCGTGTTCGTGATGATCT
GGGTCCGTATCTGGCCGAACGTTGGGCAAGCCTGGTTGATCATCCGATTGTTGGTGAA
GCACGTAGCCTGGGCCTGATGGGTGCACTGGAATTAGTTGCAGATAAAACCACCGGTC
AGCGTTTTGATAAAAGTTTAGGTGCAGGTAATCTGTGTCGTGATCTGTGTTTTGCCAATG
GTCTGGTTATGCGTAGCGTTGGCGATACCATGATTATTAGCCCTCCGCTGGTTATTCGT
CGCGAAGAAATTGATGAACTGGTGGAACTGGCACGTCGTGCCCTGGATGAAACCGCAC
GCCAGCTGACCCAGGTTCCGCATACACAAGAAGAACCGACCGCAGGTGGTGGTGGTAG
TGCCCATATTGTTATGGTTGATGCCTATA

SC-Gre2p-SC-His (this work)

TATGGTGGATACCCTGAGCGGTCTGAGCAGCGAACAGGGTCAGAGCGGTGATATGACC
ATTGAAGAAGATAGCGCAACCCACATCAAATTCAGCAAACGTGATGAAGATGGTAAAGA
ACTGGCAGGCGCAACCATGGAACTGCGTGATAGCAGCGGTAAAACCATTAGCACCTGG
ATTAGTGATGGTCAGGTGAAAGATTTTTATCTGTACCCTGGCAAATACACCTTTGTTGAA
ACCGCAGCACCGGATGGTTATGAAGTTGCAACCGCAATTACCTTTACCGTTAATGAACA
GGGCCAGGTTACCGTGAATGGTAAAGCAACCAAAGGTGATGCACATATTGGTGGTGGT
GGCAGCTCAGTTTTTGTTTCAGGTGCTAACGGGTTCATTGCCCAACACATTGTCGATCT
CCTATTGAAGGAAGACTATAAGGTCATCGGTTCTGCTAGAAGTCAAGAAAAGGCCGAGA
ATTTAACGGAGGCCTTTGGTAACAATCCAAACTTCTCCATGGAAATTGTCCCAGACATAT
CTAAGCTGGACGCATTTGACCATGTTTTCCAAAAGCACGGCAAGGAGATCAAGATAGTT
CTACATACGGCCTCTCCATTCTGCTTTGATATCACTGACAGTGAACGCGATTTATTAATT
CCTGCTGTGAACGGTGTTAAGGGAATTCTCCACTCAATTAAAAAATACGCCGCTGATTCT
GTAGAACGTGTAGTTCTCACCTCTTCTTATGCAGCTGTGTTCGACATGGCAAAAGAAAAC
GATAAATCTTTAACATTTAACGAAGAATCCTGGAACCCAGCAACCTGGGAGAGTTGCCA
AAGTGACCCGGTTAGCGCCTACTGTGGTTCTAAGAAGTTTGCTGAAAAAGCAGCTTGGG
AATTTCTAGAGGAGAATAGAGACGCTGTAAAATTCGAATTAACTGCCGTTAACCCAGTTT
ACGTTTTTGGTCCGCAAATGTTTGACAAAGATGTGAAAAAACACTTGAACACATCTTGCG
AACTCGTCAACAGCTTGATGCATTTATCACCAGAGGACAAGATACCGGAACTATTTGGT
GGATACATTGATGTTCGTGATGTTGCAAAGGCTCATTTAGTTGCCTTCCAAAAGAGGGA
AACAATTGGTCAAAGACTAATCGTATCAGAGGCCAGATTTACTATGCAGGATGTTCTCGA
TATCCTTAACGAAGACTTCCCTATCCTAAAAGGCAATATTCCAGTGGGGAAACCAGGTTC
TGGTGCTACCCATAACACCCTTGGTGCTACTCTTGATAATAAAAAGAGTAAGAAATTGTT
AGGTTTCAAGTTCAGGAACTTGAAAGAGACCATTGACGACACTGCCTCCCAAATTTTAAA
ATTTGAGGGCAGAATAGGTGGTGGTGGTAGCGTTGATACCCTGAGCGGTCTGAGCAGC
GAACAGGGTCAGAGCGGTGATATGACCATTGAAGAAGATAGCGCAACCCACATCAAATT

CAGCAAACGTGATGAAGATGGTAAAGAACTGGCAGGCGCAACCATGGAACTGCGTGAT
AGCAGCGGTAAAACCATTAGCACCTGGATTAGTGATGGTCAGGTGAAAGATTTTTATCT
GTACCCTGGCAAATACACCTTTGTTGAAACCGCAGCACCGGATGGTTATGAAGTTGCAA
CCGCAATTACCTTTACCGTTAATGAACAGGGCCAGGTTACCGTGAATGGTAAAGCAACC
AAAGGTGATGCACATAT

ST-His-AlaDH (this work)

GCCCATATTGTTATGGTGGATGCATATAAACCGACCAAAGGTGGTGGTGGTAGCCACCA
TCATCATCACCATGGTATTATTGGTGTGCCGAAAGAGATCAAAAATAACGAAAATCGTGT
TGCACTGACCCCTGGTGGTGTGAGCCAGCTGATTAGCAATGGTCATCGTGTTCTGGTTG
AAACCGGTGCAGGTTTAGGTAGCGGTTTTGAAAATGAAGCATATGAAAGTGCCGGTGCC
GAAATTATTGCAGATCCGAAACAGGTTTGGGATGCAGAAATGGTCATGAAAGTTAAAGA
ACCGCTGCCGGAAGAATATGTGTATTTTCGTAAAGGTCTGGTGCTGTTTACCTATCTGCA
TCTGGCAGCAGAACCGGAACTGGCACAGGCACTGAAAGATAAAGGTGTTACCGCAATT
GCCTATGAAACCGTTAGCGAAGGTCGTACCCTGCCGCTGCTGACCCCGATGAGCGAAG
TTGCAGGTCGTATGGCAGCACAGATTGGTGCACAGTTTCTGGAAAAACCGAAAGGTGGT
AAAGGTATTCTGCTGGCAGGCGTTCCGGGTGTTAGCCGTGGTAAAGTTACCATTATTGG
CGGTGGTGTTGTTGGCACCAATGCAGCAAAAATGGCAGTTGGTCTGGGTGCAGATGTT
ACAATTATTGCCCGTAATGCAGATCGTCTGCGTCAGCTGGATGATATTTTTGGTCATCAG
ATTAAAACCCTGATCAGCAATCCGGTTAATATTGCCGATGCAGTTGCAGAAGCCGATCT
GCTGATTTGTGCAGTTCTGATTCCGGGTGCAAAAGCACCGACACTGGTTACCGAAGAAA
TGGTGAAACAAATGAAACCGGGTAGCGTTATTGTTGATGTTGCAATTGATCAAGGTGGC
ATTGTGGAAACCGTTGATCATATTACCACACATGATCAGCCGACCTATGAAAAACATGGT
GTTGTGCATTATGCCGTTGCAAATATGCCAGGTGCAGTTCCGCGTACCAGCACCATTGC
GCTGACCAATGTTACCGTTCCGTATGCACTGCAGATTGCAAATAAAGGTGCCGTTAAAG
CACTGGCAGATAATACCGCACTGCGTGCAGGTCTGAATACCGCAAATGGCCATGTTACC
TATGAAGCAGTTGCACGTGATCTGGGTTATGAATATGTTCCGGCAGAAAAAGCACTGCA
GGATGAAAGCAGCGTTGCCGGTGCA

His-ST-ChPPK (this work)

ATGGGCAGCAGCCATCACCATCATCATCATGGTGCACACATAGTAATGGTAGACGCCTA
CAAGCCGACGAAGGGTGGTGGTGGCAGCGCAACCGATTTTAGCAAACTGAGCAAATAT
GTTGAAACCCTGCGTGTTAAACCGAAACAGAGCATTGATCTGAAAAAGGATTTCGATAC
CGACTACGATCATAAAATGCTGACCAAAGAAGAAGGCGAAGAACTGCTGAATCTGGGTA
TTTCAAAACTGAGCGAGATCCAAGAAAAACTGTATGCAAGCGGCACCAAAAGCGTTCTG
ATTGTTTTTCAGGCAATGGATGCAGCAGGTAAAGATGGCACCGTTAAACATATTATGACC
GGTCTGAATCCGCAGGGTGTTAAAGTTACCAGCTTTAAAGTTCCGAGCAAAATCGAACT
GAGCCATGATTATCTGTGGCGTCATTATGTTGCACTGCCTGCAACCGGTGAAATTGGTA
TCTTTAATCGTAGCCACTATGAAAATGTTCTGGTTACCCGTGTTCATCCGGAATATCTGC
TGAGCGAACAGACCAGCGGTGTTACCGCAATTGAACAGGTTAATCAGAAATTCTGGGAT
AAACGCTTTCAGCAGATCAACAACTTTGAACAGCATATTAGCGAAAACGGCACCATTGT
GCTGAAATTCTTTCTGCATGTTAGCAAGAAAGAGCAGAAAAAGCGTTTTATCGAACGCAT
TGAACTGGACACCAAAAACTGGAAATTTAGCACAGGCGATCTGAAAGAACGTGCACATT
GGAAAGATTATCGCAACGCCTATGAAGATATGCTGGCAAATACCAGCACCAAACAGGCA
CCGTGGTTTGTTATTCCGGCAGATGATAAATGGTTTACCCGTCTGCTGATTGCCGAAATT
ATCTGTACCGAACTGGAAAAACTGAATCTGACCTTTCCGACCGTTAGCCTGGAACAGAA
AGCAGAGCTGGAAAAAGCAAAAGCAGAACTGGTTGCAGAAAAGTCCAGCGATTAATGA

His-SC-ChPPK (this work)

ATGCATCATCACCATCATCATGGTGTGGATACCCTGAGCGGTCTGAGCAGCGAACAGG
GTCAGAGCGGTGATATGACCATTGAAGAAGATAGCGCAACCCACATCAAATTCAGCAAA
CGTGATGAAGATGGCAAAGAACTGGCAGGCGCAACCATGGAACTGCGTGATAGCAGCG
GTAAAACCATTAGCACCTGGATTAGTGATGGTCAGGTGAAAGATTTTTATCTGTATCCGG
GTAAATATACCTTCGTTGAAACCGCAGCACCGGATGGTTATGAAGTTGCAACCGCAATT

ACCTTTACCGTGAATGAACAAGGTCAGGTTACCGTTAATGGTAAAGCAACCAAAGGTGA
TGCACATATTGGTGGTGGTGGCAGCGCAACCGATTTTAGCAAACTGAGCAAATATGTTG
AAACCCTGCGTGTTAAACCGAAACAGAGCATTGATCTGAAAAAGGATTTCGATACCGAC
TACGATCATAAAATGCTGACCAAAGAAGAAGGCGAAGAACTGCTGAATCTGGGTATTTC
AAAACTGAGCGAGATCCAAGAAAAACTGTATGCAAGCGGCACCAAAAGCGTTCTGATTG
TTTTTCAGGCAATGGATGCAGCAGGTAAAGATGGCACCGTTAAACATATTATGACCGGT
CTGAATCCGCAGGGTGTTAAAGTTACCAGCTTTAAAGTTCCGAGCAAAATCGAACTGAG
CCATGATTATCTGTGGCGTCATTATGTTGCACTGCCTGCAACCGGTGAAATTGGTATCTT
TAATCGTAGCCACTATGAAAATGTTCTGGTTACCCGTGTTCATCCGGAATATCTGCTGAG
CGAACAGACCAGCGGTGTTACCGCAATTGAACAGGTTAATCAGAAATTCTGGGATAAAC
GCTTTCAGCAGATCAACAACTTTGAACAGCATATTAGCGAAAACGGCACCATTGTGCTG
AAATTCTTTCTGCATGTTAGCAAGAAAGAGCAGAAAAAGCGTTTTATCGAACGCATTGAA
CTGGACACCAAAAACTGGAAATTTAGCACAGGCGATCTGAAAGAACGTGCACATTGGAA
AGATTATCGCAACGCCTATGAAGATATGCTGGCAAATACCAGCACCAAACAGGCACCGT
GGTTTGTTATTCCGGCAGATGATAAATGGTTTACCCGTCTGCTGATTGCCGAAATTATCT
GTACCGAACTGGAAAAACTGAATCTGACCTTTCCGACCGTTAGCCTGGAACAGAAAGCA
GAGCTGGAAAAAGCAAAAGCAGAACTGGTTGCAGAAAAGTCCAGCGATTAATGA

ST-EsPAD^{d}-ST-His (Y19F, Y21F, E72S) (this work)

ATGGCTCACATCGTTATGGTGGACGCTTACAAACCGACCAAAGGTGGTGGTGGTAGCA
ACACCTTCGACAAACATGATCTGAGCGGTTTTGTTGGTAAACATCTGGTGTATACCTATG
ATAATGGCTGGGAGTATGAGATCTATGTGAAAAATGAAAACACCCTGGATTATCGCATTC
ATAGCGGTCTGGTTGGTAATCGTTGGGTTAAAGATCAGCAGGCATATATTGTTCGTGTG
GGTGAAAGCATCTATAAAATCAGCTGGACCGAACCGACCGGCACCGATGTTAGCCTGAT
TGTTAATCTGGGTGATAGCCTGTTTCATGGCACCATCTTTTTTCCGCGTTGGGTGATGAA
TAATCCGGAAAAAACCGTTTGCTTTCAGAACGATCATATTCCGCTGATGAATAGCTATCG
TGATGCAGGTCCGGCATATCCGACCGAAGTTATTGATGAATTTGCCACCATTACCTTTGT
TCGTGATTGTGGTGCAAATAACGAAAGCGTTATTGCATGTGCAGCAAGCGAACTGCCGA
AAAACTTTCCGGATAATCTGAAAGGTGGTGGTGGTAGCGCACATATTGTTATGGTTGAT
GCATATAAACCGACCAAAGGTCATCATCACCATCATCATTAA

BsGDH-ST-His (Peschke et al. 2018)[20]

ATGTATCCGGATTTAAAAGGAAAAGTCGTCGCTATTACAGGAGCTGCTTCAGGGCTCGG
AAAGGCAATGGCCATTCGCTTCGGCAAGGAGCAGGCAAAAGTGGTTATCAACTATTATA
GTAATAAACAAGATCCGAACGAGGTAAAAGAAGAGGTCATCAAGGCGGGCGGTGAAGC
TGTTGTCGTCCAAGGAGATGTCACGAAAGAGGAAGATGTAAAAAATATCGTGCAAACGG
CAATTAAAGAGTTCGGCACACTCGATATTATGATTAATAATGCCGGTCTTGAAAATCCTG
TGCCATCTCACGAAATGCCGCTTAAGGATTGGGATAAAGTCATCGGCACAAACTTAACA
GGTGCCTTTTTAGGAAGCCGTGAAGCGATTAAATATTTCGTAGAAAACGATATCAAGGG
AAATGTCATCAACATGTCCAGTGTGCACGAAGTGATTCCTTGGCCGTTATTTGTTCACTA
CGCGGCAAGTAAAGGCGGGATAAAGCTGATGACAGAAACATTGGCGTTGGAATACGCG
CCGAAGGGCATTCGCGTGAACAATATCGGGCCAGGTGCGATCAATACGCCAATCAATG
CTGAAAAATTTGCTGACCCTAAACAGAAAGCAGATGTAGAAAGCATGATTCCGATGGGG
TATATCGGCGAACCGGAGGAGATCGCCGCAGTGGCAGTGTGGCTTGCTTCGAAGGAAT
CCAGCTATGTTACAGGCATCACATTGTTTGCGGACGGCGGAATGACGAAATATCCTTCT
TTCCAGGCAGGACGCGGTGGTGGTGGTGGTAGCGGTGGTGGTGGTAGCGCCCATATT
GTTATGGTGGATGCATATAAACCGACCAAAGGTCATCATCACCATCACCATTGA

BsGDH-SC-His (Peschke et al. 2018)[20]

ATGTATCCGGATTTAAAAGGAAAAGTCGTCGCTATTACAGGAGCTGCTTCAGGGCTCGG
AAAGGCAATGGCCATTCGCTTCGGCAAGGAGCAGGCAAAAGTGGTTATCAACTATTATA
GTAATAAACAAGATCCGAACGAGGTAAAAGAAGAGGTCATCAAGGCGGGCGGTGAAGC
TGTTGTCGTCCAAGGAGATGTCACGAAAGAGGAAGATGTAAAAAATATCGTGCAAACGG
CAATTAAAGAGTTCGGCACACTCGATATTATGATTAATAATGCCGGTCTTGAAAATCCTG

TGCCATCTCACGAAATGCCGCTTAAGGATTGGGATAAAGTCATCGGCACAAACTTAACA
GGTGCCTTTTTAGGAAGCCGTGAAGCGATTAAATATTTCGTAGAAAACGATATCAAGGG
AAATGTCATCAACATGTCCAGTGTGCACGAAGTGATTCCTTGGCCGTTATTTGTTCACTA
CGCGGCAAGTAAAGGCGGGATAAAGCTGATGACAGAAACATTGGCGTTGGAATACGCG
CCGAAGGGCATTCGCGTGAACAATATCGGGCCAGGTGCGATCAATACGCCAATCAATG
CTGAAAAATTTGCTGACCCTAAACAGAAAGCAGATGTAGAAAGCATGATTCCGATGGGG
TATATCGGCGAACCGGAGGAGATCGCCGCAGTGGCAGTGTGGCTTGCTTCGAAGGAAT
CCAGCTATGTTACAGGCATCACATTGTTTGCGGACGGCGGAATGACGAAATATCCTTCT
TTCCAGGCAGGACGCGGTGGTGGTGGTGGTAGCGGTGGTGGTGGTAGCGTTGATACC
CTGAGCGGTCTGAGCAGCGAACAGGGTCAGAGCGGTGATATGACCATTGAAGAAGATA
GCGCAACCCACATCAAATTCAGCAAACGTGATGAAGATGGTAAAGAACTGGCAGGCGC
AACCATGGAACTGCGTGATAGCAGCGGTAAAACCATTAGCACCTGGATTAGTGATGGTC
AGGTGAAAGATTTTTATCTGTACCCTGGCAAATACACCTTTGTTGAAACCGCAGCACCG
GATGGTTATGAAGTTGCAACCGCAATTACCTTTACCGTTAATGAACAGGGCCAGGTTAC
CGTGAATGGTAAAGCAACCAAAGGTGATGCACATATTGGTCATCATCACCATCACCATT
GA

His-PDZ-Binding-Domain-LbADH (Hertel et al. 2025)[25]

ATGCATCATCACCACCATCATGGTCTGCAGCGTCGTCGTGTTACCGTTCGTAAAGCAGA
TGCCGGTGGTCTGGGTATTAGCATTAAAGGTGGTCGTGAAAACAAAATGCCGATCCTGA
TTAGCAAAATCTTTAAAGGTCTGGCAGCAGATCAGACCGAAGCACTGTTTGTTGGTGAT
GCAATTCTGAGCGTTAATGGTGAAGATCTGAGCAGCGCAACCCATGATGAAGCAGTTCA
GGCACTGAAAAAAACCGGTAAAGAAGTTGTTCTGGAAGTCAAATACATGAAAGAAGTGA
GCCCGTACTTTAAAGGTGGTGGTGGTAGCAGCAATCGCCTGGATGGTAAAGTTGCAATT
ATTACCGGTGGCACCCTGGGTATTGGTCTGGCAATTGCAACCAAATTTGTTGAAGAGGG
TGCCAAAGTTATGATTACCGGTCGTCATAGTGATGTTGGTGAAAAAGCAGCAAAAAGCG
TTGGTACACCGGATCAGATTCAGTTTTTTCAGCATGATAGCAGTGATGAAGATGGTTGG
ACCAAACTGTTTGATGCAACCGAAAAAGCATTTGGTCCGGTTAGCACCCTGGTTAATAAT
GCAGGTATTGCAGTGAATAAAAGCGTGGAAGAAACCACCACCGCAGAATGGCGTAAAC
TGCTGGCAGTTAATCTGGATGGTGTTTTTTTTGGTACACGTCTGGGTATTCAGCGCATGA
AAAACAAAGGTCTGGGTGCAAGCATTATCAACATGAGCAGCATTGAAGGTTTTGTTGGT
GATCCGAGCCTGGGTGCATATAATGCAAGCAAAGGTGCAGTTCGTATTATGAGCAAAAG
CGCAGCACTGGATTGTGCACTGAAAGATTATGATGTTCGTGTGAATACCGTTCATCCGG
GTTATATCAAAACACCGCTGGTTGATGATCTGCCTGGTGCCGAAGAAGCAATGAGCCAG
CGTACCAAAACCCCGATGGGTCATATTGGTGAACCGAATGATATTGCCTATATCTGTGTT
TATCTGGCCAGCAACGAAAGTAAATTTGCAACCGGTAGCGAATTTGTTGTGGATGGTGG
TTATACCGCACAGTAATAA

BsGDH-PDZ-Ligand-His (Hertel et al. 2025)[25]

ATGTATCCGGATTTAAAAGGAAAAGTCGTCGCTATTACAGGAGCTGCTTCAGGGCTCGG
AAAGGCAATGGCCATTCGCTTCGGCAAGGAGCAGGCAAAAGTGGTTATCAACTATTATA
GTAATAAACAAGATCCGAACGAGGTAAAAGAAGAGGTCATCAAGGCGGGCGGTGAAGC
TGTTGTCGTCCAAGGAGATGTCACGAAAGAGGAAGATGTAAAAAATATCGTGCAAACGG
CAATTAAAGAGTTCGGCACACTCGATATTATGATTAATAATGCCGGTCTTGAAAATCCTG
TGCCATCTCACGAAATGCCGCTTAAGGATTGGGATAAAGTCATCGGCACAAACTTAACA
GGTGCCTTTTTAGGAAGCCGTGAAGCGATTAAATATTTCGTAGAAAACGATATCAAGGG
AAATGTCATCAACATGTCCAGTGTGCACGAAGTGATTCCTTGGCCGTTATTTGTTCACTA
CGCGGCAAGTAAAGGCGGGATAAAGCTGATGACAGAAACATTGGCGTTGGAATACGCG
CCGAAGGGCATTCGCGTGAACAATATCGGGCCAGGTGCGATCAATACGCCAATCAATG
CTGAAAAATTTGCTGACCCTAAACAGAAAGCAGATGTAGAAAGCATGATTCCGATGGGG
TATATCGGCGAACCGGAGGAGATCGCCGCAGTGGCAGTGTGGCTTGCTTCGAAGGAAT
CCAGCTATGTTACAGGCATCACATTGTTTGCGGACGGCGGAATGACGAAATATCCTTCT
TTCCAGGCAGGACGCGGTGGTGGTGGTGGTAGCGGTGGTGGTGGTAGCGGTGTTAAA

GAATCTCTGGTTGGTCATCATCACCATCACCATTGA

His-SH3-Binding-Domain-LbADH (Hertel et al. 2025)[25]

ATGCATCATCACCACCATCATGGTGCAGAATATGTTCGTGCCCTGTTTGATTTTAATGGC
AACGATGAAGAAGATCTGCCGTTCAAAAAAGGTGATATTCTGCGTATTCGTGACAAACC
GGAAGAACAGTGGTGGAATGCAGAAGATAGCGAAGGTAAACGTGGTATGATTCCGGTT
CCGTATGTGGAAAAATATGGTGGTGGTGGTAGCAGCAATCGCCTGGATGGTAAAGTTG
CAATTATTACCGGTGGCACCCTGGGTATTGGTCTGGCAATTGCAACCAAATTTGTTGAA
GAGGGTGCCAAAGTTATGATTACCGGTCGTCATAGTGATGTTGGTGAAAAAGCAGCAAA
AAGCGTTGGTACACCGGATCAGATTCAGTTTTTTCAGCATGATAGCAGTGATGAAGATG
GTTGGACCAAACTGTTTGATGCAACCGAAAAAGCATTTGGTCCGGTTAGCACCCTGGTT
AATAATGCAGGTATTGCAGTGAATAAAAGCGTGGAAGAAACCACCACCGCAGAATGGCG
TAAACTGCTGGCAGTTAATCTGGATGGTGTTTTTTTTGGTACACGTCTGGGTATTCAGCG
CATGAAAAACAAAGGTCTGGGTGCAAGCATTATCAACATGAGCAGCATTGAAGGTTTTG
TTGGTGATCCGAGCCTGGGTGCATATAATGCAAGCAAAGGTGCAGTTCGTATTATGAGC
AAAAGCGCAGCACTGGATTGTGCACTGAAAGATTATGATGTTCGTGTGAATACCGTTCA
TCCGGGTTATATCAAAACACCGCTGGTTGATGATCTGCCTGGTGCCGAAGAAGCAATGA
GCCAGCGTACCAAAACCCCGATGGGTCATATTGGTGAACCGAATGATATTGCCTATATC
TGTGTTTATCTGGCCAGCAACGAAAGTAAATTTGCAACCGGTAGCGAATTTGTTGTGGAT
GGTGGTTATACCGCACAGTAA

BsGDH-SH3-Ligand-His (Hertel et al. 2025)[25]

ATGTATCCGGATTTAAAAGGAAAAGTCGTCGCTATTACAGGAGCTGCTTCAGGGCTCGG
AAAGGCAATGGCCATTCGCTTCGGCAAGGAGCAGGCAAAAGTGGTTATCAACTATTATA
GTAATAAACAAGATCCGAACGAGGTAAAAGAAGAGGTCATCAAGGCGGGCGGTGAAGC
TGTTGTCGTCCAAGGAGATGTCACGAAAGAGGAAGATGTAAAAAATATCGTGCAAACGG
CAATTAAAGAGTTCGGCACACTCGATATTATGATTAATAATGCCGGTCTTGAAAATCCTG
TGCCATCTCACGAAATGCCGCTTAAGGATTGGGATAAAGTCATCGGCACAAACTTAACA
GGTGCCTTTTTAGGAAGCCGTGAAGCGATTAAATATTTCGTAGAAAACGATATCAAGGG
AAATGTCATCAACATGTCCAGTGTGCACGAAGTGATTCCTTGGCCGTTATTTGTTCACTA
CGCGGCAAGTAAAGGCGGGATAAAGCTGATGACAGAAACATTGGCGTTGGAATACGCG
CCGAAGGGCATTCGCGTGAACAATATCGGGCCAGGTGCGATCAATACGCCAATCAATG
CTGAAAAATTTGCTGACCCTAAACAGAAAGCAGATGTAGAAAGCATGATTCCGATGGGG
TATATCGGCGAACCGGAGGAGATCGCCGCAGTGGCAGTGTGGCTTGCTTCGAAGGAAT
CCAGCTATGTTACAGGCATCACATTGTTTGCGGACGGCGGAATGACGAAATATCCTTCT
TTCCAGGCAGGACGCGGTGGTGGTGGTGGTAGCGGTGGTGGTGGTAGCCCGCCGCCG
GCGCTGCCGCCGAAACGTCGTCGTGGTCATCATCACCATCACCATTG

His-SC-LbADH (Peschke et al. 2018)[20]

ATGCATCATCACCACCATCATGGTGTTGATACCCTGAGCGGTCTGAGCAGCGAACAGG
GTCAGAGCGGTGATATGACCATTGAAGAAGATAGCGCAACCCACATCAAATTCAGCAAA
CGTGATGAAGATGGTAAAGAACTGGCAGGCGCAACCATGGAACTGCGTGATAGCAGCG
GTAAAACCATTAGCACCTGGATTAGTGATGGTCAGGTGAAAGATTTTTATCTGTACCCTG
GCAAATACACCTTTGTTGAAACCGCAGCACCGGATGGTTATGAAGTTGCAACCGCAATT
ACCTTTACCGTTAATGAACAGGGCCAGGTTACCGTGAATGGTAAAGCAACCAAAGGTGA
TGCACATATTGGTGGTGGTGGTAGCAGCAATCGCCTGGATGGTAAAGTTGCAATTATTA
CCGGTGGCACCCTGGGTATTGGTCTGGCAATTGCAACCAAATTTGTTGAAGAGGGTGC
CAAAGTTATGATTACCGGTCGTCATAGTGATGTTGGTGAAAAAGCAGCAAAAAGCGTTG
GTACACCGGATCAGATTCAGTTTTTTCAGCATGATAGCAGTGATGAAGATGGTTGGACC
AAACTGTTTGATGCAACCGAAAAAGCATTTGGTCCGGTTAGCACCCTGGTTAATAATGCA
GGTATTGCAGTGAATAAAAGCGTGGAAGAAACCACCACCGCAGAATGGCGTAAACTGCT
GGCAGTTAATCTGGATGGTGTTTTTTTTGGTACACGTCTGGGTATTCAGCGCATGAAAAA
CAAAGGTCTGGGTGCAAGCATTATCAACATGAGCAGCATTGAAGGTTTTGTTGGTGATC
CGAGCCTGGGTGCATATAATGCAAGCAAAGGTGCAGTTCGTATTATGAGCAAAAGCGCA

GCACTGGATTGTGCACTGAAAGATTATGATGTTCGTGTGAATACCGTTCATCCGGGTTAT
ATCAAAACACCGCTGGTTGATGATCTGCCTGGTGCCGAAGAAGCAATGAGCCAGCGTA
CCAAAACCCCGATGGGTCATATTGGTGAACCGAATGATATTGCCTATATCTGTGTTTATC
TGGCCAGCAACGAAAGTAAATTTGCAACCGGTAGCGAATTTGTTGTGGATGGTGGTTAT
ACCGCACAGTAA

Table S2. List of used primers and their corresponding sequences.

Primer	DNA sequence	Used for
AD002	GATCCGGCTGCTAACAAAGCCCG	Linearization for insertion of EcYeiN
AD003	ATGTATATCTCCTTCTTAAAGTTAAAC AAAATTATTTCTAGAGG	Linearization for insertion of EcYeiN
AD007	TAATGGTAGACGCCTACAAGCCGACG AAGGGCGGTGGTGGTAGCAGCG	Insertion of N-term. ST
AD008	GCGTCTACCATTACTATGTGTGCACC ATGATGATGGTGGTGATGCATATG	Insertion of N-term. ST
AD010	GGTAAAGCAACCAAAGGTGATGCACA TATTGGCGGTGGTGGTAGCAGCG	Linearization for insertion of SC
AD011	TCGCTGCTCAGACCGCTCAGGGTATC CACACCATGATGATGGTGGTGATGCA TATG	Linearization for insertion of SC
AD012	AATATGTGCATCACCTTTGGTTGCTTT ACC	Amplification of SC
AD013	GTGGATACCCTGAGCGGTCTGAG	Amplification of SC

Table S3: Specific activities of native and genetically modified enzyme variants used for AEH bead formation.

**Enzyme**	**Specific activity [μmol$_{substrate}$ min^{-1} mg$_{protein}$$^{-1}$]**	Relative activity [%]	Reference
EsPAD	30.95 ± 9.6	100	This work
ST-EsPAD-ST	40.40 ± 2.3	131	This work
EsPAD-SC	19.20 ± 6.0	62	This work
EsPAD AEH bead[1)]	0.73 ± 0.2	2.8	This work
EcYeiN	8.3 ± 1.0	100	This work
EcYeiN-ST	10.3 ± 1.2	124	This work

EcYeiN-SC	9.6 ± 1.2	116	This work
LbADH	13.7 ± 0.8	100	[20], [24]
SC-LbADH	6.6 ± 0.6	48	[20], [24]
$SH3^B$-LbADH	2.79 ± 0.16	20	[25]
PDZ^B-LbADH	13.95 ± 0.13	101	[25]
BsGDH	3.6 ± 0.4	100	[20], [24]
BsGDH-ST	4.3 ± 0.4	119	[20], [24]
BsGDH-$SH3^L$	5.75 ± 0.21	159	[25]
BsGDH-PDZ^L	5.36 ± 0.66	149	[25]
HEWT	n.d.[2)]	n.d.[2)]	This work
ST-HEWT-ST	n.d.[2)]	n.d.[2)]	This work
Gre2p	5.9 ± 0.17	100	[21]
SC-Gre2p-SC	3.8 ± 0.03	64.4	[21]
AlaDH	46.18 ± 3.5	100	This work
AlaDH-ST	25.37 ± 1.8	54.9	This work
ChPPK	0.0025	100	This work
ST-ChPPK	0.0019	76	This work
SC-ChPPK	0.0015	60	This work

1) The value determined for AEH beads represents an apparent mass-normalized heterogeneous catalyst activity and is therefore not directly equivalent to the intrinsic specific activities determined for soluble enzymes.

2) Specific activities of HEWT and ST-HEWT-ST were not determined because the aldehyde intermediate generated within the cascade is unstable, precluding a reliable activity comparison under the reaction conditions relevant to this study

References

[54] D. G. Gibson, L. Young, R. Y. Chuang, J. C. Venter, C. A. Hutchison, 3rd, H. O. Smith, Nat Methods 2009, 6, 343.
[55] S. Brunauer, P. H. Emmett, E. Teller, J. Am. Chem. Soc. 1938, 60, 309.
[56] M. Peng, M. Franzreb, A. J. Weber, P. Lemke, C. M. Niemeyer, K. S. Rabe, ChemCatChem 2024, 16, e202400092.
[57] P. Gaspers, P. Lemke, A. Delavault, C. M. Domínguez, K. S. Rabe, C. M. Niemeyer, Chem. Eur. J. 2024, e202403047.
[58] D. M. Murphy, T. Koop, Q. J. R. Meteorol. Soc. 2005, 131, 1539.
